# Rapamycin Increases the Development Competence of Yak (*Bos grunniens*) Oocytes by Promoting Autophagy via Upregulating 17β-Estradiol and HIF-1α During In Vitro Maturation

**DOI:** 10.3390/ani15030365

**Published:** 2025-01-27

**Authors:** Meng Wang, Xin Ma, Qian Zhang, Hui Zhang, Shantong Qiu, Ruihua Xu, Yangyang Pan

**Affiliations:** 1 College of Veterinary Medicine, Gansu Agricultural University, Lanzhou 730070, China; wangmeng@gsau.edu.cn (M.W.); mxinmaxin@163.com (X.M.); zq880204@126.com (Q.Z.); zhui199527@163.com (H.Z.); qiushantong1998@163.com (S.Q.); xu_rh1@163.com (R.X.); 2 Gansu Province Livestock Embryo Engineering Research Center, Lanzhou 730070, China

**Keywords:** rapamycin, autophagy, oocyte maturation, HIF-1α, E2 synthesis, yak

## Abstract

High-quality oocyte production strategies play an important role in animal-assisted reproductive biotechnologies, and rapamycin (Rap) has been commonly used to increase the development potential of mammalian oocytes. The purpose of this study is to evaluate the effects and possible molecular mechanisms of rap on the maturation of yak oocytes. Different concentrations of Rap were supplemented during in vitro maturation (IVM) of yak oocytes. The maturation rates of oocytes and development rates of parthenogenetically activated embryos were assessed. The levels of 17β-estradiol (E2) were detected via ELISA, and the expression of autophagy-related factors, steroidogenic enzymes, and HIF-1α was detected via qRT-PCR, western blotting, and fluorescence microscopy, respectively. In addition, the impacts of E2 and HIF-1α on Rap-mediated oocyte autophagy were investigated by investigating the activities of E2 and HIF-1α. Our results showed that 0.1 nM Rap substantially enhanced the developmental ability of yak oocytes. In this group, the levels of E2, CYP19A1, CYP17A1, and autophagy-related factors were also significantly increased, and the expression of ATG5 and BECN1 in subsequent embryos was also increased. Further analysis revealed that Rap tends to enhance the development competence of yak oocytes and that the levels of autophagy-related factors are reduced when the activity of E2 or HIF-1α is inhibited. Furthermore, the levels of E2, CYP19A1, and CYP17A1 were downregulated when the activity of HIF-1α was inhibited, and the levels of HIF-1α were also significantly reduced by the estrogen receptor antagonist G15. Nevertheless, the levels of CYP11A1 mRNA in mature yak COCs were not significantly different among these groups, a phenomenon which implies that the levels of E2 were not correlated with the CYP11A1 content in yak COCs. There was an increasing tendency for the development competence of yak oocytes at the optimum concentration of Rap during IVM. The potential underlying mechanism is that Rap can activate autophagy and upregulate the levels of E2 and HIF-1α in mature oocytes. Additionally, the levels of both E2 and HIF-1α are regulated by each other and involve Rap-regulated autophagy in oocytes.

## Simple Summary

The purpose of this study is to investigate the effect of Rap on yak oocyte maturation and its underlying molecular mechanisms. During in vitro maturation (IVM), different concentrations of Rap were administered to evaluate both the maturation rate and embryo development rate. The levels of E2 were measured using ELISA, while the expression of autophagy factors, steroidogenic enzymes, and HIF-1α was analyzed through qRT-PCR, western blot, and immunofluorescence techniques. It was found that 0.1 nM Rap significantly enhanced the developmental capacity of yak oocytes and increased the levels of E2, aromatase, CYP17A1, and various autophagy factors. Inhibition of E2 or HIF-1α diminished the effects of Rap. Furthermore, the inhibition of HIF-1α resulted in decreased levels of E2, aromatase, and CYP17A1, while estrogen receptor antagonists were observed to reduce HIF-1α levels. These results indicate that appropriate concentrations of Rap can promote autophagy by upregulating 17β-estradiol and HIF-1α, thereby improving the developmental potential of yak oocytes. Additionally, E2 and HIF-1α appear to mutually regulate each other in the context of Rap-induced autophagy.

## 1. Introduction

Mammalian oocyte in vitro maturation (IVM) is a valuable method for investigating the molecular mechanisms that regulate reproduction in females, and a high-quality oocyte production strategy can be used to improve animal reproductive technologies. Recently, a growing body of research has attempted to increase the development competence of oocytes in several mammals through the supplementation of reproductive hormones [1,2], growth factors [3], and small molecule drugs [4,5], all of which regulate oocyte quality via multiple signaling pathways, such as apoptosis, reactive oxygen species (ROS) [5], mitochondrial function, cumulus expansion, and autophagy [1]. Autophagy is one of the basic physiological processes in eukaryotic cells and is involved in degrading and recycling damaged cell components to maintain cellular homeostasis [6,7]. In addition, autophagy promotes the adaptation of cells to stress, such as hypoxia and starvation [8]. Hypoxia-inducible factors (HIFs) induce autophagy by enhancing N6-methyladenosine (m6A) epigenetic remodeling under hypoxia [9]. Autophagy plays a key role in mammalian oocyte maturation and embryo development and can be regulated by rapamycin (Rap) [10] and 17β-estradiol (E2) [1,11] during IVM. Aberrant mitochondria in oocytes can be degraded by autophagy [12], and the induction of autophagy can activate DNA demethylation in mouse-cloned embryos [10]; more studies in different animals are needed to elucidate the regulatory mechanisms involved.

Rap is an immunosuppressive drug produced by Streptomyces hygroscopicus [13] that has been widely used to improve the development potential of mammalian oocytes and embryos [5,14,15]. Rap is usually used as a specific inhibitor of the mechanistic target of rapamycin (mTOR) and helps increase tolerance to environmental stress factors in mammals [16], such as starvation, refrigeration, reactive oxygen species [5], and DNA damage [15], these effects being related to the levels of E2 in cardiomyocytes [17], and the levels of reproductive hormones can be elevated by Rap in mice [18]. It has been reported that Rap inhibits the physiological effect of mTOR through specific pharmacological activation of autophagy [19,20]. In addition, Rap has been confirmed to have various biological functions that regulate aging, metabolism, cell viability, and differentiation [19]. Research in recent years has revealed the important roles of Rap during mammalian oocyte maturation, as confirmed in bovine [5], porcine [21], and mouse [15], which can increase the levels of melatonin [14], telomere maintenance, and DNA damage repair. Similarly, several investigations have reported a dose-dependent effect on oocytes during IVM in various species [5,15,21]. However, the effects of the molecular mechanisms of Rap during yak (*Bos grunniens*) IVM have yet to be studied.

Yaks are seasonally polyestrous mammals with relatively low reproductive efficiency due to the cold and low-oxygen environment in which they live [22,23]. The development potential of yak oocytes in vitro is also lower than that of other animals of the same species [24]. Hence, the development of yak reproductive and breeding biotechnologies, such as in vitro fertilization (IVF) and somatic cell nuclear transfer (SCNT), has been strongly hindered by these physiological features. Our previous research revealed that E2 increases yak oocyte development by regulating cytokines that are involved in cumulus expansion and that the levels of oocyte-secreted factors (OSFs) are also increased by exogenous E2 [25]. The effect of E2 on HIF-1α is affected by the oxygen concentration and may be relevant to cell type [26]. In addition, HIF-1α inhibits the apoptosis of yak oocytes via the upregulation of the vascular endothelial growth factor (VEGF) under hypoxic conditions [24]. Recent studies have demonstrated that HIF-1α is involved in E2 synthesis in granulosa cells during hypoxia via elevated levels of steroidogenic enzymes, such as cytochrome P450 family 11 subfamily A member 1 (CYP11A1) [27], cytochrome P450 17alpha-hydroxylase/17,20-lyase (CYP17A1), and aromatase (CYP19A1) [28,29]. The estrogen receptor β can be upregulated by HIF-1α in pulmonary artery endothelial cells [30]. Collectively, these findings emphasize that the correlation between HIF-1α and E2 during mammalian oocyte maturation remains largely elusive.

Inspired by these previous studies, one objective of the current study is to assess the effects of Rap on the developmental competence of yak oocytes to verify the optimal concentration of Rap. Additionally, we analyze whether the inhibition of E2 and HIF-1α affects the autophagy level of yak oocytes. These findings provide a promising method for increasing the quality of mature yak oocytes in vitro, which may help promote the development of yak assisted reproductive and breeding biotechnology.

## 2. Materials and Methods

### 2.1. Chemicals and Antibodies

Unless otherwise mentioned, all the chemicals and reagents used in the present study were obtained from Sigma Chemical (St. Louis, MO, USA). An inhibitor of HIF-α activity (PX-478) was purchased from Selleck Chemical (Houston, TX, USA), and a selective estrogen receptor antagonist (G15) was acquired from Cayman Chemical (Ann Arbor, MI, USA).

### 2.2. Collection of Yak COCs

**Figure 1:**
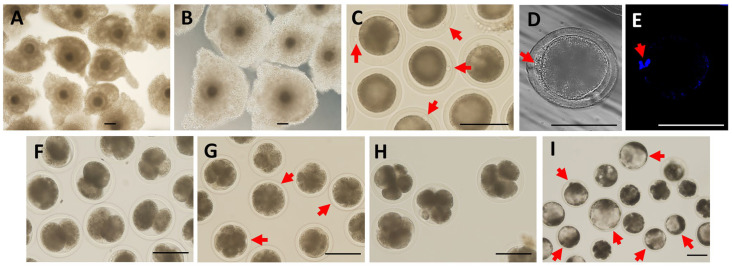
Observation of the maturation of yak COCs and the development of arthenogenetically activated embryos. (**A**) The immature COCs were selected on the basis of the characteristics of intact cumulus cells and homogeneous cytoplasm for maturation culture. (**B**) Mature COCs after IVM for 24 h. (**C**,**D**) Mature oocytes with an extruded first polar body were defined as mature at the MII stage, and the red arrows indicate the first polar body. (**E**) Two bright spots of chromatin were observed on mature oocytes stained with DAPI. (**F**–**I**) represent the development of parthenogenetically activated yak embryos at the two-cell, four-cell, morula, and blastocyst stages, all of which are indicated by red arrows. Bar = 200 μm.

Ovaries from postpubertal yaks were collected in a local slaughterhouse and were kept in thermally insulated bottles containing physiological saline with penicillin (100 IU·mL^−1^) and streptomycin (10 mg·mL^−1^) at a temperature of 28–30 °C and were subsequently transported to the laboratory within 2 h of slaughtering. After washing, the samples were washed with warm Dulbecco’s phosphate-buffered saline (DPBS) three times at 28–30 °C. Follicular fluid from normal follicles with a diameter of 2–10 mm was aspirated via an 18-gauge needle in a 10 mL syringe. Immature cumulus-oocyte complexes (COCs) were selected for subsequent study on the basis of their characteristics of intact cumulus cells and homogeneous cytoplasm (Figure 1A). The selected COCs were rinsed more than two times in the tissue culture medium 199 (TCM-199), which was supplemented with 5% fetal bovine serum (FBS; Medicorp, Montreal, QC, Canada), and kept in humidified air (5% CO_2_) at 37 °C for 2 h to equilibrate the pH before use. All procedures for the collection of yak COCs in the laboratory were completed in less than 1 h.

### 2.3. Yak COCs In Vitro Maturation and Rap, G15, and PX-478 Treatment

The in vitro maturation medium for yak COCs was optimized in TCM-199 plus 10% FBS, 10% porcine follicle fluid, 50 mg·mL^−1^ gentamicin, 200 mM pyruvate, 50 mg·mL^−1^ follicle-stimulating hormone, and 1.0 μg·mL^−1^ luteinizing hormone (Ningbo Second Hormone, Ningbo, China). In accordance with the experimental design, different concentrations of Rap (0, 0.1, 1, and 1.0 nM) were supplemented with reference to the concentrations used for bovine and mouse oocyte maturation [5,15]. The optimum concentrations of Rap plus G15 (G15 is a high-affinity and selective antagonist that can effectively inhibit endogenous estrogen) (1 μM) [31] or PX-478 (PX-478 is an effective HIF-1α inhibitor which can interfere with its binding to the promoter region of the target gene by inhibiting the activity of HIF-1α) (25 µM) [32] were also designed to evaluate the effects of E2 and HIF-1α on the autophagy of Rap during yak oocyte IVM. A total of 40–50 yak COCs were randomly selected and cultured in each well of four-well dishes (Nunc, Roskilde, Denmark) with 400 µL of culture medium from different experimental design groups, and COCs from all the wells in one of the four-well dishes were used as a group. For IVM, all dishes containing yak COCs were kept under 5% CO_2_ in humidified air at 38 °C for 24 h.

### 2.4. Assessment of the Maturation Rate of Oocytes

Yak maturation oocytes were assessed following the methods described by Duan et al. [1]. Briefly, after IVM for 24 h, mature COCs were collected and washed with DPBS three times (Figure 1B). Mature COCs were divided into two sets in each group: the first set was used to measure the levels of steroidogenic enzymes, autophagy, and HIF-1α, and the remaining COCs were collected to assess the maturation rate of oocytes and parthenogenetic activation. The COCs were washed three times and incubated with 1 mg·mL^−1^ hyaluronidase at 37 °C for 3–5 min to remove cumulus cells by gentle pipetting, and the denuded oocytes were collected for subsequent studies. Oocytes under a stereomicroscope (ZX100; Olympus, Tokyo, Japan) with an extruded first polar body were defined as mature at the MII stage (Figure 1C,D), in which they were stained with 5 mg·mL^−1^ 40-6-diamidino-2-phenylindole (DAPI) for 5 min in the dark. Two bright spots of chromatin were observed under an inverted fluorescence microscope (Leica, Solms, Germany) (Figure 1E).

### 2.5. Parthenogenetic Activation and Embryo Culture

The matured oocytes were rinsed in a modified synthetic oviductal fluid medium (mSOF) three times, activated with 5 µM ionomycin at 38 °C for 3–5 min, and then cultured for 4–6 h in mSOF containing 2 mM 6-dimethylaminopurine in an incubator at 38 °C under 5% CO_2_ in humidified air. Subsequently, 20 activated oocytes were transferred into 100 µL droplets of mSOF with 5 mg·mL^−1^ bovine serum albumin (BSA) and covered with 2.5 mL of mineral oil in 100 µM dishes for embryo culture in humidified air with 5% CO_2_ at 38 °C. Yak oocytes were washed three times between each step in the medium that the next step used and each time for 5 min. The development rates of yak embryos at the two-cell (Figure 1F), four-cell (Figure 1G), morula (Figure 1H), and blastocyst (Figure 1I) stages in the control and optimum concentrations of Rap were investigated after 36, 48, 96, and 192 h, respectively. In each repetition, 20 embryos at each stage were collected from the two groups for autophagy assessments.

### 2.6. Determination of Estradiol by ELISA

The culture medium was collected after the mature COCs were removed from the control and all treatment groups and used to determine the concentrations of E2. The levels of E2 were detected via a commercially available estrogen enzyme-linked immunosorbent assay (ELISA) (REN JIRBIO, Shanghai, China) following the manufacturer’s procedures.

### 2.7. Quantitative Real-Time Polymerase Chain Reaction (qRT-PCR)

After 20 mature COCs or 40 embryos in each treatment were rinsed three times in DPBS (without Ca^2+^ and Mg^2+^), total RNA extraction was performed via the RNeasy Micro Kit (Qiagen, Valencia, CA, USA) and the RNA was reverse-transcribed into 20 µL of cDNA via the Superscript III First-Strand Synthesis Kit (Invitrogen, Chicago, IL, USA) according to the manufacturer’s instructions. The cDNA samples were kept frozen at −20 °C until subsequent studies.

The steroidogenic genes, autophagy genes, and HIF-1α were selected and the levels of relevant mRNAs were compared via quantitative real-time PCR with the Light Cycler^®^ 96 software (Roche, Mannheim, Germany). Information on the primer pairs used for real-time PCR is provided in Table 1. The reactions in a total volume of 20 µL consisted of 2 µL of cDNA, 10 μL of 2× SYBR Green master mix (Takara, Dalian, China), 0.5 μL of forward primer, 0.5 μL of reverse primer at a concentration of 10 µM, and 7.0 μL of ddH2O. The cycling conditions for the Light Cycler^®^ 96 were 95 °C for 1 min, followed by 40 cycles, including denaturation for 10 s at 95 °C, annealing for 10 s at the appropriate temperature (Table 1), and extension fluorescence acquisition for 15 s at 72 °C. Melting curves were analyzed before amplification to confirm the specificity of the qRT-PCR primers. β-actin was selected as a housekeeping gene for cDNA normalization according to our previous studies [24,25], and the 2^−ΔΔCT^ method was used to evaluate the relative levels of mRNAs in each group [33]. Mature yak oocytes and embryos from the group without Rap were included in the control group.

### 2.8. Western Blot Analysis

Fifty mature COCs were selected and solubilized in 18 μL of Laemmli sample buffer supplemented with 1 mM phenylmethanesulfonyl fluoride (Beyotime, Shanghai, China) on ice. The supernatant was collected, blended with 2 μL of 5×SDS-PAGE sample loading buffer, and boiled for 10 min at 100 °C for protein denaturation. Equal volumes of total protein from each sample were separated via 12% sulfate polyacrylamide gel electrophoresis and transferred to 0.22 μm polyvinylidene fluoride (PVDF) membranes (Bio-Rad Laboratories, Hercules, CA, USA). The membranes were blocked with a solution of tris-buffered saline with tween 20 (TBST) containing 5% (*w*/*v*) nonfat dry milk for 1 h under slight shaking at 37 °C. The primary antibodies used were chosen according to the experimental design, diluted according to the information in Table S1, and then incubated with the membranes at 4 °C for 12 h. Horseradish peroxidase-conjugated secondary antibodies were selected and incubated with the membranes at 37 °C for 1 h. The protein ladder was visualized on X-ray film via an enhanced chemiluminescence detection kit (Beyotime, Shanghai, China). The membrane was washed three times between each step in TBST at 37 °C with slight shaking and then washed for 10 min. Statistical analyses of protein variations were performed via a densitometric analysis system (Biotanon, Shanghai, China), and β-actin was used to normalize the bands. The original western blots are provided in the Supplementary File S1.

### 2.9. Immunofluorescence Staining

To stain proteins in mature COCs and embryos, they were collected and fixed in fresh 4% paraformaldehyde at room temperature for 2 h, permeabilized with 1% Triton X-100 (Solarbio, Beijing, China) in DPBS for 10 min, and blocked with 3% bovine serum albumin (BSA) in PBS for 1 h. The COCs and embryos were incubated with primary antibodies overnight at 4 °C, followed by incubation with secondary antibodies that were stained with different types of fluorescence at room temperature for 1 h. Then, the COCs and embryos were incubated for 3 min with 5 ng·mL^−1^ DAPI for nuclear labeling. The COCs and embryos were washed three times between each step in PBS at room temperature with gentle pipetting and then washed for 5 min. After the last wash, the COCs and embryos were mounted, fluorescence was observed via a fluorescence microscope (DeltaVision Elite, GE Healthcare, Buckinghamshire, UK), and images were acquired via the SoftWoRx 5.5 software with a Cool SNAP HQ camera (Photometrics, Tucson, AZ, USA).

### 2.10. Statistical Analyses

At least three replicates were included in each treatment, and the results are expressed as the means and standard errors of the means (SEMs). Statistical analyses were performed via IBM SPSS Statistics (v22.0) and GraphPad Prism 9.0 (San Diego, CA, USA), followed by Tukey–Kramer multiple comparison tests. A chi-square test was used for the statistics of the maturation rate and development rate. A *p*-value lower than 0.05 was considered a statistically significant difference.

## 3. Results

### 3.1. Rap Enhanced the Developmental Competence of Yak OOCYTES

The maturation rates of yak oocytes after treatment with 0.1 nM (91.74 ± 4.65%) and 1.0 nM (91.40 ± 0.86%) Rap significantly increased compared with those in the control treatments (85.48 ± 2.26%), and the maturation rates did not significantly differ between these two groups (*p* > 0.05) (Table 2). However, supplementation with TCM-199 and 10 nM Rap (85.34 ± 1.58%) significantly decreased the maturation rates of yak oocytes compared with those under the other doses of Rap treatments (Table 2). Consequently, the optimal concentration of Rap during yak oocyte IVM was 0.1 nM, and the early embryonic development rates after parthenogenetic activation were investigated at this concentration, the results of which are shown in Table 3. The results revealed that the embryonic development rates at the four stages in the 0.1 nM Rap treatment group were also significantly greater (*p* < 0.05) than those in the control group (Table 3), a finding which implies that 0.1 nM Rap supplementation during IVM strengthened the development of yak embryos until the blastocyst stage.

### 3.2. Inhibiting the Endogenous E2 or HIF-1α Affects the Developmental Competence of Oocytes

Owing to the effects of Rap on the developmental competence of yak oocytes, we explored whether the endogenous E2 could inhibit these effects; the estrogen receptor antagonists G15 and 0.1 nM Rap were both used in the yak COC maturation system. As shown in Table 2, the maturation rate of yak oocytes in this group was significantly lower than that in the 0.1 nM Rap group (*p* < 0.05), and it was also significantly lower than that in the control group (*p* < 0.05). The early development rates of mature yak oocytes after parthenogenetic activation were also significantly lower in the 0.1 nM Rap + G15 group (*p* < 0.05) (Table 3). In addition, the HIF-α activity inhibitors PX-478 and 0.1 nM Rap were both used in the yak COC IVM system to evaluate whether HIF-1α could regulate the effects of Rap on the development of yak oocytes. The results revealed that both the maturation rate and the embryonic development rate could be significantly diminished by HIF-1α, which was also significantly lower than that in the control group (*p* < 0.05) (Table 2 and Table 3).

### 3.3. Rap Promotes the Level of E2 During Yak COC IVM

**Figure 2:**
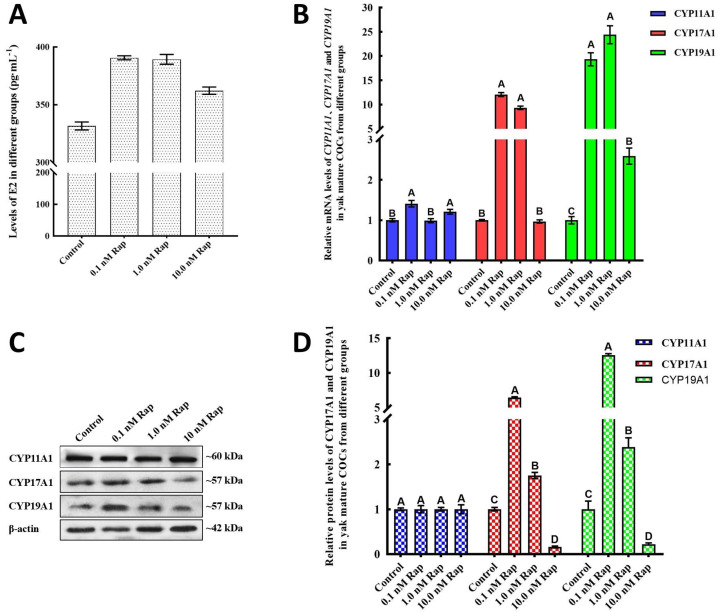
Rap increases the levels of E2 and steroidogenic enzymes during IVM of yak COCs. (**A**) The E2 levels increased significantly in the Rap treatment groups. (**B**) CYP17A1 and CYP19A1 mRNA levels increased significantly in COCs from the groups treated with 0.1 and 1.0 mM Rap, whereas there were no significant changes in CYP11A1 mRNA levels. (**C**) Protein expression of CYP17A1 and CYP19A1 in yak COCs from the groups treated with different concentrations of Rap was detected by western blot. (**D**) Intensity analysis of the blot band; the intensity was much greater in the 0.1 and 1.0 mM Rap groups than in the control groups, whereas it was lower in the 10 mM Rap group. The values are expressed as the means ± SEMs of *n* = 3. Superscripts (A, B, C, D) show significant differences (*p* < 0.05).

E2 levels in the maturation medium were detected by ELISA kits at different concentrations. As shown in Figure 2A, the levels of E2 in the groups treated with Rap were markedly greater than those in the control group (*p* < 0.05), and there was no significant difference in E2 levels between the groups treated with 0.1 nM and 1.0 nM Rap (*p* > 0.05). However, the level of E2 in the 10 nM Rap group significantly increased compared with that in the control groups which was significantly lower than that in the other concentrations of Rap groups (*p* < 0.05). Consistent with this finding, the mRNA and protein levels of the steroidogenic enzymes CYP17A1 and CYP19A1 in yak mature COCs were markedly increased by 0.1 nM and 1.0 nM Rap (*p* < 0.05) (Figure 2B–D). There was no significant change in the levels of CYP11A1 mRNA and protein among the four groups (Figure 2B,D).

### 3.4. Rap Increased the Expression of HIF-1α During Yak COC IVM

**Figure 3:**
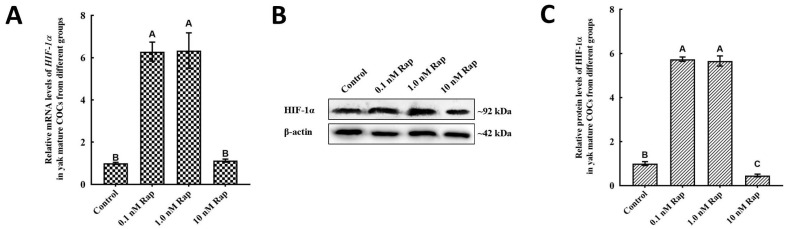
Rap induces HIF-1α expression in yak mature COCs. mRNA (**A**) and protein (**B**) expression of HIF-1α in mature yak COCs treated with different concentrations of Rap. (**C**) Blot band intensity analysis of the HIF-1α protein. The results are shown as the mean ± SEM of *n* = 3. Superscripts (A, B, C) indicate significant differences, *p* < 0.05.

The relationship between HIF-1α and Rap during yak oocyte IVM was further studied. As expected, 0.1 nM and 1.0 nM Rap greatly increased the mRNA and protein levels of HIF-1α (*p* < 0.05) (Figure 3), and there was no significant difference in E2 levels between these groups (*p* > 0.05). However, the protein and mRNA levels of HIF-1α substantially decreased in the group treated with 10 nM Rap (*p* < 0.05) and were not significantly different from the levels in the control group (*p* > 0.05) (Figure 3). The levels of HIF-1α were evidently upregulated in a dose-dependent manner during yak oocyte IVM.

### 3.5. Rap-Induced Autophagy in Mature COCs and Early Embryos

**Figure 4:**
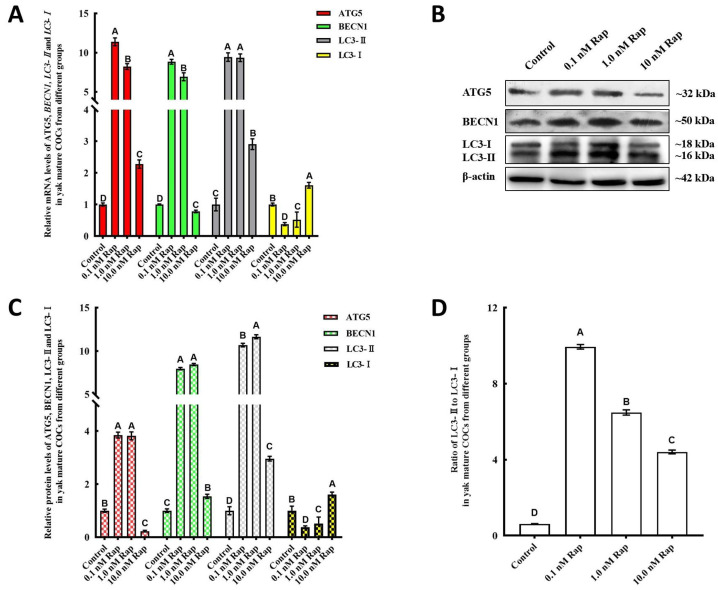
Levels of autophagy-related factors in yak mature COCs from different groups. (**A**) mRNA levels of ATG5, BECN1, LC3-I, and LC3-II in mature COCs from different groups. (**B**) Protein expression of ATG5, BECN1, LC3-I, and LC3-II in yak COCs from groups treated with different concentrations of Rap was detected by western blot. (**C**) Blot band intensity analysis of the ATG5, BECN1, LC3-I, and LC3-II proteins. (**D**) Ratios of LC3II/LC3I in different groups. Values with different superscripts (A, B, C, D) are significantly different (*p* < 0.05).

The effects of Rap on the autophagy of mature COCs and early embryos were evaluated on the basis of the levels of autophagy-related factors (ATG5, BECN1 and LC3). For the different concentrations of Rap, the expression of ATG5, BECN1, and LC3-II mRNAs and proteins was evidently increased in mature COCs from the groups with 0.1 nM and 1.0 nM Rap (*p* < 0.05) (Figure 4A–C), whereas the expression of LC3-I was significantly lower than that in the control group (*p* < 0.05). The ratio of LC3II/LC3I (a useful marker for autophagy) in the yak mature COCs from the 0.1 nM and 1.0 nM Rap groups was significantly increased at the protein level compared with that in the control groups (Figure 4D), and the ratio was highest in the 0.1 nM Rap group. The expression of most autophagy-related factors was significantly reduced in the 10 nM Rap group (Figure 4A–C). The ratio of LC3II/LC3I in the 10 nM Rap group was significantly lower than that in the 0.1 nM and 1.0 nM Rap groups; however, it was still higher than that in the control group (Figure 4D).

**Figure 5:**
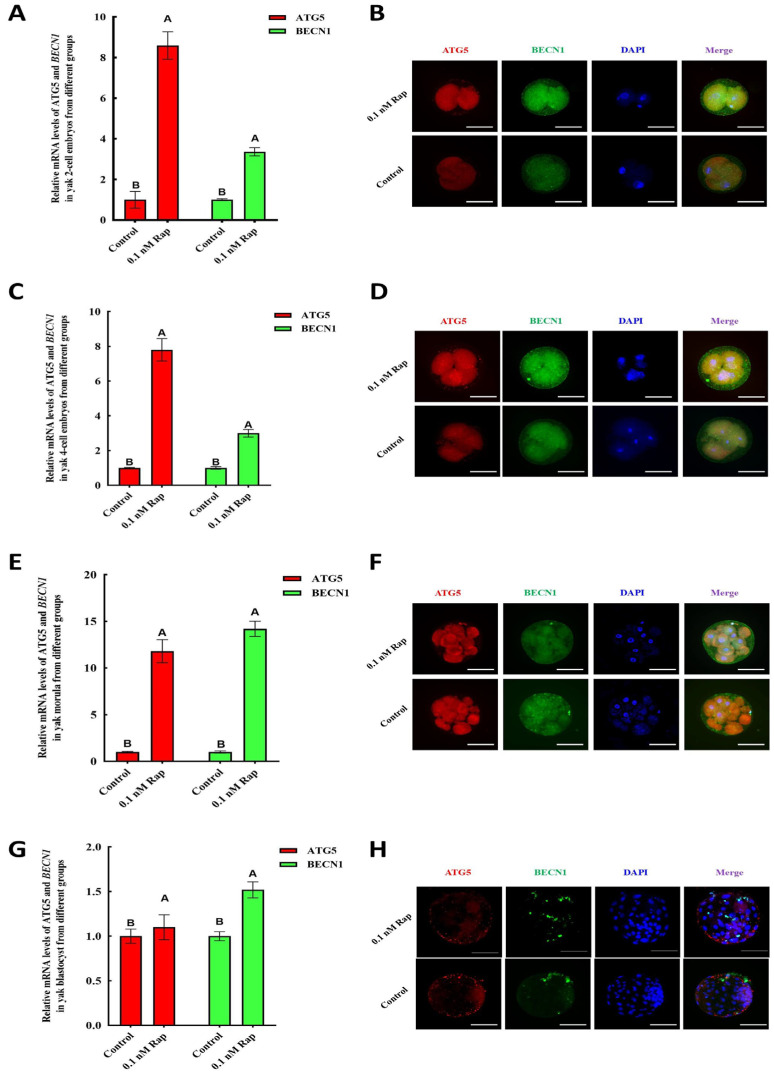
Levels of ATG5 and BECN1 in parthenogenetically activated yak embryos from control and 0.1 nM Rap groups. (**A**) mRNA levels of ATG5 and BECN1 in two-cell embryos. (**B**) Fluorescence of the ATG5 (red) and BECN1 (green) proteins in two-cell embryos. (**C**) mRNA levels of ATG5 and BECN1 in four-cell embryos. (**D**) Fluorescence of the ATG5 (red) and BECN1 (green) proteins in four-cell embryos. (**E**) mRNA levels of ATG5 and BECN1 in morula embryos. (**F**) Fluorescence of the ATG5 (red) and BECN1 (green) proteins in morula embryos. (**G**) mRNA levels of ATG5 and BECN1 in blastocysts. (**H**) Fluorescence of the ATG5 (red) and BECN1 (green) proteins in blastocysts. Values in A, C, E, and G are expressed as mean ± SEM of *n* = 3. Superscripts (A, B) show significant differences, *p* < 0.05. Bar = 200 μm in (**B**,**D**,**F**,**H**).

In accordance with the levels of autophagy-related factors in yak mature COCs, the mRNA levels of ATG5 and BECN1 also showed an elevated expression in early embryos from the 0.1 nM Rap groups compared with the control group (Figure 5A–G). Additionally, the protein fluorescence of ATG5 and BECN1 could be detected in all embryos from four stages, and the fluorescence intensity in embryos from the 0.1 nM Rap groups was significantly higher than that from the control group (Figure 5B,D,F,H).

### 3.6. Inhibiting the Endogenous E2 Activity Downregulates HIF-1α and Autophagy

**Figure 6:**
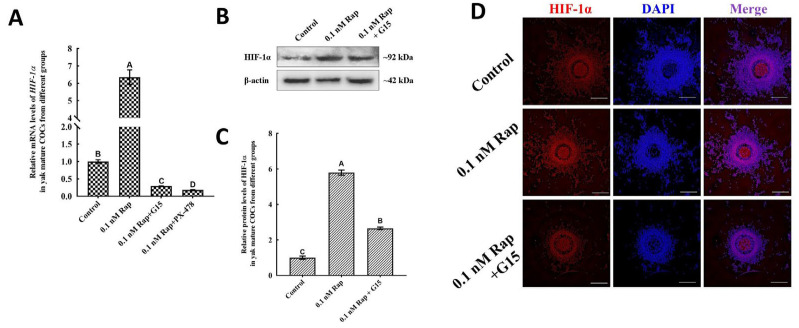
The inhibitory activity of E2 influenced the effect of Rap-induced HIF-1α. (**A**) The levels of HIF-1α mRNA decreased significantly in the 0.1 nM Rap + G15 treatment groups. (**B**) Protein expression of HIF-1α in yak COCs from the 0 nM Rap, 0.1 nM Rap, and 0.1 nM Rap + G15 groups was detected by western blot. (**C**) Blot band intensity analysis of the HIF-1α protein. (**D**) Immunofluorescence analysis of HIF-1α (red) protein in yak COCs. Superscripts (A, B, C, D) show significant differences, *p* < 0.05 in (**A**), Bar = 200 μm in (**D**).

**Figure 7:**
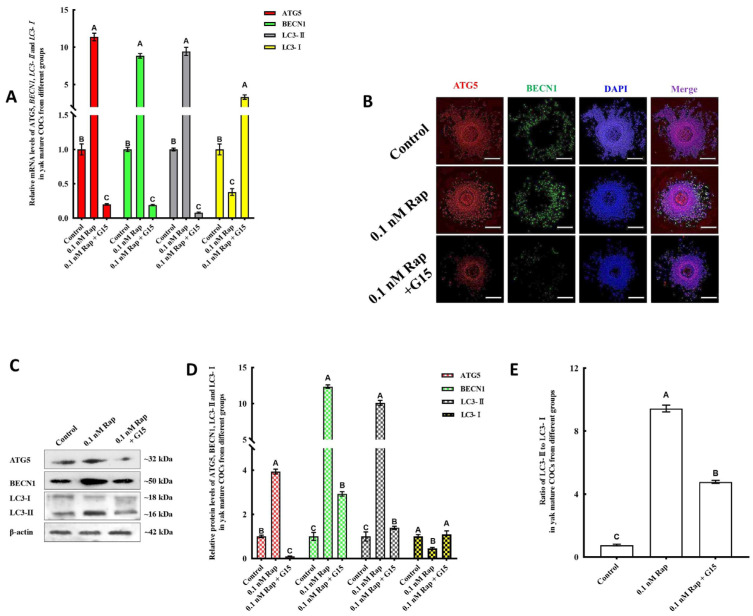
The effect of Rap-induced autophagy was influenced by the inhibition of E2 activity. (**A**) ATG5, BECN1, and LC3-Ⅱ mRNA levels decreased significantly in the 0.1 nM Rap + G15 treatment groups. (**B**) Immunofluorescence analysis of ATG5 (red) and BECN1 (green) proteins in yak COCs. (**C**) Protein expression of ATG5, BECN1, LC3-I, and LC3-II in yak COCs from 0 nM Rap, 0.1 nM Rap, and 0.1 nM Rap + G15 groups were detected by western blot. (**D**) Blot band intensity analysis of ATG5, BECN1, LC3-I, and LC3-II proteins. (**E**) Ratios of LC3II/LC3I in different groups. Values with different superscripts (A, B, C) are significantly different (*p* < 0.05). Bar = 200 μm in (**B**).

**Figure 8:**
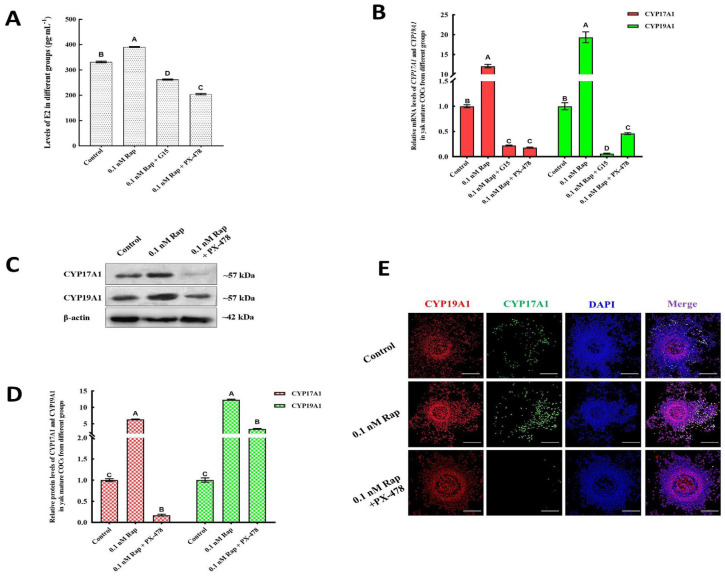
The effect of Rap-induced E2 synthesis was influenced by the inhibitory activity of HIF-1α. (**A**) E2 levels decreased significantly in the 0.1 nM Rap + PX-478 treatment groups. (**B**) The levels of CYP17A1 and CYP 19A1 decreased significantly in the 0.1 nM Rap + PX-478 treatment groups. (**C**) Protein expression of ATG5, CYP17A1, and CYP 19A1 in yak COCs from the 0 nM Rap, 0.1 nM Rap, and 0.1 nM Rap + G15 groups was detected via western blotting. (**D**) Blot band intensity analysis of the CYP17A1 and CYP19A1 proteins. (**E**) Immunofluorescence analysis of the CYP17A1 (red) and CYP19A1 (green) proteins in yak COCs. Superscripts (A, B, C, D) show significant differences, *p* < 0.05 in (**A**). Bar = 200 μm in (**E**).

The essential functions of the endogenous E2 in enhancing the impact of Rap-mediated HIF-1α and autophagy were further investigated by the addition of 1 µM G15, an estrogen receptor antagonist. As shown in Figure 6A–C, the expression levels of HIF-1α mRNA and protein in the 0.1 nM Rap + G15 group were significantly lower than those in the 0.1 nM Rap group (*p* < 0.05), and the levels of E2, CYP19A1, and CYP17A1 mRNAs in the 0.1 nM Rap + G15 group were significantly lower than those in the control groups (*p* < 0.05) (Figure 8A,B), a phenomenon which indicates that the endogenous E2 synthesis during IVM could be inhibited by 1 µM G15. Compared with those in mature COCs in the 0.1 nM Rap group, the mRNA and protein levels of autophagy-related factors were significantly lower in mature COCs treated with 0.1 nM Rap or G15 (*p* < 0.05) (Figure 7A,C–E). The proteins of HIF-1α, ATG5, and BECN1 were also stained by immunofluorescence and are shown in Figure 6D and Figure 7B. As shown in Figure 6D and Figure 7B, weaker fluorescence was observed in yak mature COCs from the 0.1 nM Rap + G15 group compared to those from the 0.1 nM Rap group, and the fluorescence intensity was brightest in mature COCs from the 0.1 nM Rap group. Additionally, both HIF-1α and ATG5 proteins could be detected in yak cumulus cells and oocytes in COCs from the 0.1 nM Rap group (Figure 6D and Figure 7B), whereas BECN1 was detected only in cumulus cells (Figure 7B). These results demonstrate that, after the endogenous E2 was inhibited, the ability of Rap to upregulate HIF-1α and induce autophagy during IVM was also inhibited.

### 3.7. Inhibiting the Levels of HIF-1α Results in Reduced E2 Synthesis and Autophagy

**Figure 9:**
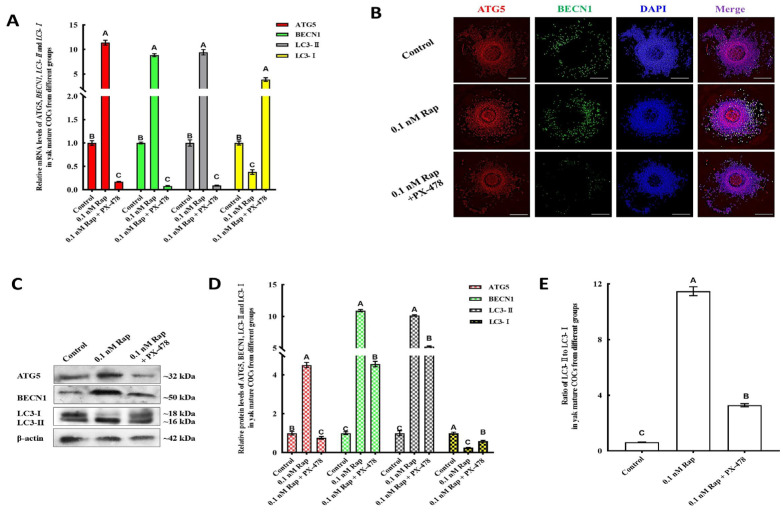
The effect of Rap-induced autophagy was influenced by the inhibitory activity of HIF-1α (**A**). The levels of ATG5, BECN1, and LC3-II mRNAs decreased significantly in the 0.1 nM Rap + PX-478 treatment groups. (**B**) Immunofluorescence analysis of the ATG5 (red) and BECN1 (green) proteins in yak COCs. (**C**) Protein expression of ATG5, BECN1, LC3-I, and LC3-II in yak COCs from the 0 nM Rap, 0.1 nM Rap, and 0.1 nM Rap + PX-478 groups were detected by western blot. (**D**) Blot band intensity analysis of the ATG5, BECN1, LC3-I, and LC3-II proteins. (**E**) Ratios of LC3II/LC3I in different groups. Values with different superscripts (A, B, C) are significantly different (*p* < 0.05). Bar = 200 μm in (**B**).

The biological effects of HIF-1α suppression on Rap-mediated E2 synthesis and autophagy were assessed by treatment with 25 µM PX-478, an inhibitor of HIF-α activity. Unlike those in the 0.1 nM Rap-treated group, the endogenous E2, CYP17A1, and CYP19A1 levels in the 0.1 nM Rap + PX-478 group were substantially diminished (*p* < 0.05) (Figure 8A–D), and the mRNA level of HIF-1α in the 0.1 nM Rap + PX-478 group was markedly reduced compared with that in the control treatment group (*p* < 0.05) (Figure 6A), implying that HIF-α activity in yak COCs could be inhibited by 25 µM PX-478. In addition, the combination of 0.1 nM Rap and PX-478 greatly reduced the expression of ATG5 and BECN1 at the mRNA and protein levels (*p* < 0.05) (Figure 9A,C–E), diminishing the positive effects of Rap on autophagy in mature yak COCs. As shown in Figure 8E and Figure 9B, the fluorescence intensity of CYP17A1, CYP19A1, and autophagy-related factors (ATG5 and BECN) in mature yak COCs from the 0.1 nM Rap + PX-478 group was weaker than that in the 0.1 nM Rap group, a phenomenon which further confirmed our results that the potential of HIF-1α to Rap promoted E2 synthesis and induced autophagy during the IVM of yak COCs. In addition, the fluorescence of CYP19A1 in COCs from all groups was brighter than that of CYP17A1, and CYP19A1 was predominantly distributed on cumulus cells (Figure 8E). The fluorescence distributions of ATG5 and BECN in the 0.1 nM Rap + PX-478 group (Figure 9B) were similar to those in the 0.1 nM Rap + G15 group.

## 4. Discussion

Rap has been applied to improve the development ability of oocytes in various mammals for different purposes. Previous studies have shown that the developmental competence of pigs with morphologically poor oocytes could be improved via the induction of autophagy during IVM with 1 nM Rap [21]. Additionally, 1 nM Rap, as a potential lifespan extender, can increase the maturation rates of bovine oocytes by maintaining telomere length [5], and blastocyst formation is also influenced by the regulation of mRNAs that remain pluripotent, such as the Nanog homeobox (NANOG) and the sex-determining region Y-box 2 (SOX2), especially in low-quality oocytes [34,35]. In experiments using mouse oocytes, Rap at 10 nM was selected as the optimal concentration for improving the developmental competence and quality of IVM oocytes via multiple molecular mechanisms [15]. These findings indicate that the effects of rapamycin on the developmental competence of oocytes are concentration-dependent and have potential relevance to different species. In our present study, there was no significant difference in the effects of Rap on the maturation of 0.1 and 1.0 nM yak oocytes, whereas, when 10 nM Rap was used during IVM, an inhibitory effect on oocyte maturation was observed. Therefore, we hypothesize that 0.1 nM is the optimal concentration during IVM of yak COCs and evaluated its effects on embryo development in our subsequent experiments. Furthermore, the toxic effect of Rap on oocytes could be prevented during IVM at higher concentrations, such an effect having been reported in bovines and mice [5,34]. Determining the optimal dose of rapamycin will help to increase the developmental potential of yak oocytes. The results of this study reveal that supplementation with 0.1 nM Rap during IVM not only increased the percentage of mature oocytes, but also promoted the development of parthenogenetically activated embryos. This study can better solve the problem of low reproductive efficiency of yaks due to low oxygen and colder environments.

The quality of mammalian oocytes plays an important role in maintaining early embryonic viability, which also impacts the establishment of pregnancy and normal fetal development [36]. In particular, autophagy has been verified to increase oocyte quality by regulating the proliferation and differentiation of ovarian granulosa cells [7], the adaptation of oocytes to stress [37,38], and the degradation of maternal factors [39]. Rap can activate autophagy by negatively regulating the function of mTOR [40,41] and is broadly applied in various mammalian cells to activate autophagy [42,43]. In studies utilizing porcine oocytes, autophagy in COCs was sufficiently induced by regulating the relative transcript levels of BECN 1 and LC3 mRNAs at 1 nM Rap during IVM [21], a phenomenon which is generally consistent with the findings of our study, and the levels of autophagy-related factors were significantly increased in mature yak COCs and parthenogenetically activated embryos from the group treated with 0.1 nM Rap. Its maturation and development rates have also increased significantly. These data indicate that Rap can regulate autophagy during the IVM of mammalian COCs and that even the autophagy levels in subsequent embryos are affected by the Rap used in IVM. Nevertheless, more studies are needed in other mammals to explore the effects and molecular mechanism of autophagy induction by Rap during IVM.

Systemically administered Rap can increase the levels of reproductive hormones, such as melatonin in rats [14], testosterone in mice [18], and the expression of steroidogenesis genes, which Rap also regulates in mammals. Consistent with previous findings, our findings that the addition of Rap elevated E2 levels during the IVM of yak COCs, for the first time, demonstrate that Rap could regulate E2 synthesis. Furthermore, CYP11A1, CYP17A1, and CYP19A1 are key steroidogenic enzymes, and that their levels correlate with E2 synthesis has been reported in mammals [29,44,45]. However, the level of CYP11A1 mRNA in mature yak COCs was not significantly different among these groups with different concentrations of Rap (Figure 2A), a phenomenon which implies that the level of E2 was not correlated with the CYP11A1 content in yak COCs, a finding which, in turn, may be related to the physiological properties of different species. In recent years, increasing research has revealed that the mTOR-rictor complex 2 (mTORC2) plays a role in meiotic maturation by influencing actin-dependent asymmetric division in mammalian oocytes [46,47], such a complex, as a novel moderator, contributing to the regulation of autophagy [48].

Additionally, the sensitivity of mTORC2 to Rap could be regulated by E2 in adaptive cardiac remodeling [17]. Autophagy events in mammalian oocytes regulated by E2 have been confirmed in pigs [1,11] and mice [7]. The effects of high levels of E2 on the autophagy of yak COCs should be addressed, and further investigation is needed to explore the relationship between the effects of Rap and E2 on autophagy. In this study, the endogenous E2 activity was effectively suppressed by the addition of an estrogen receptor antagonist. The specific mechanism is that G15 inhibits E2-stimulated GPER-mediated proliferation and signal transduction by antagonizing GPR30 receptors, thereby reducing the synthesis of estrogen, and estrogen is strictly regulated in the body. When the estrogen receptor signaling pathway is inhibited by G15, it indirectly inhibits estrogen synthesis by affecting the activity of estrogen synthesis-related enzymes or triggering feedback regulation mechanisms. These mechanisms, together, constitute the multiple roles of G15 in inhibiting estrogen, indicating that G15 diminished the positive effects of Rap on yak COC maturation, and the autophagy levels also decreased in the same group. These findings are consistent with the ability of E2 to induce the autophagy of Rap via the mTORC2 signaling pathway.

Another main interest of our investigation was that Rap regulated the levels of HIF-1α in mature yak COCs during IVM, such levels being positively correlated with the levels of E2 and autophagy. Similarly to our observations, the activation of autophagy by Rap in human SH-SY5Y neuroblastoma cells upregulates the expression of BECN1 and HIF-1α and enhances the protective effect against brain injury [49]. Furthermore, an in vitro study indicated that HIF-1α was positively correlated with another autophagy agonist, telomerase reverse transcriptase (TERT), resulting in the activation of autophagy [50]. Furthermore, the ability of Rap to upregulate E2 and autophagy levels was diminished when PX-478 inhibited HIF-α activity. These data suggest that HIF-1α is involved in the activation of autophagy by Rap during yak oocyte maturation.

HIF-1α stimulates adenosine triphosphate (ATP) generation in COCs by promoting the expression of mRNAs involved in glycolysis and glucose uptake, which are required for E2 synthesis, especially in a hypoxic environment [28]. The yak species was selected as a model for mechanistic research on HIF-1α in this study because reproductive physiology is highly influenced by hypoxia, which is also involved in yak oocyte maturation [24]. In this study, the level of E2 was substantially decreased by PX-478, which is used to inhibit the activity of HIF-1α. Moreover, the data revealed strong positive correlations between E2, CYP17A1, and CYP19A1 levels and HIF-1α activity inhibition. This evidence suggests that the effect of Rap-regulated E2 synthesis is affected by HIF-1α.

Interestingly, when G15 inhibited the endogenous E2 activity, the effects of Rap-upregulated HIF-1α were also diminished, indicating that E2 and HIF-1α are regulated by each other during the IVM of yak COCs. Nonetheless, research has recently demonstrated that E2 may downregulate the expression of HIF-1α in the placenta under 3% O_2_ tension [51], and the different effects of E2 on HIF-1α remain to be investigated in future studies. Overall, although different mechanisms of E2-regulated HIF-1α may exist in different species, a common factor is that both are involved in the regulation of autophagy during yak oocyte maturation. There may be many mechanisms for the effect of rapamycin on yak oocytes. The follow-up work can expand the mechanism by detecting double strand breaks, alkaline comet assay, caspase-3 activity, and other experiments.

## 5. Conclusions

**Figure 10:**
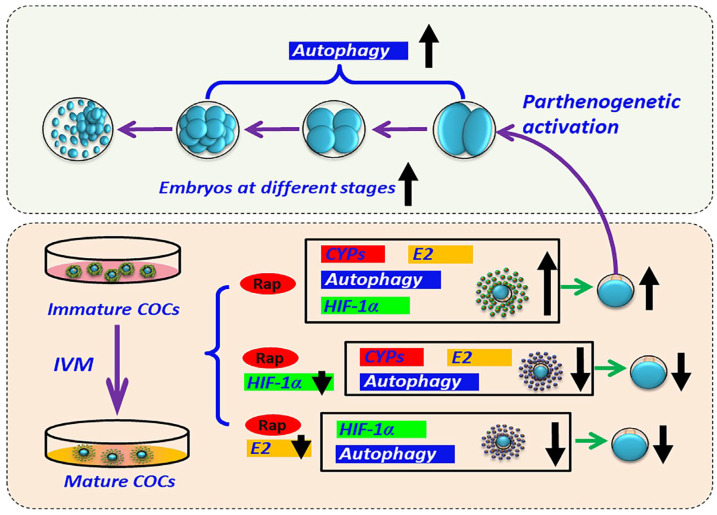
Research review: rapamycin increases the developmental competence of yak oocytes. The possible underlying mechanism is that Rap can activate autophagy and upregulate the levels of E2 and HIF-1α in mature oocytes. Additionally, the levels of both E2 and HIF-1α are regulated by each other and are involved in the regulation of autophagy by Rap in oocytes.

In conclusion, there was an increasing tendency for Rap to increase the development competence of yak oocytes at the optimum concentration during IVM. The possible underlying mechanism is that Rap can promote autophagy by upregulating 17β-estradiol and HIF-1α (Figure 10). Additionally, the levels of both E2 and HIF-1α are regulated by each other and are involved in the regulation of autophagy by Rap in oocytes (Figure 10). These findings will be useful for enhancing the quality of yak oocytes in vitro and promoting the development of yak assisted reproductive and breeding biotechnology.

## Figures and Tables

**Table 1:** Information for primers used in real-time PCR.

Gene	Primer Sequence	T_m_/°C	Amplicon Size (bp)	GenBank Accession No.
*CYP11A1*	F: TTTGCCTTTGAGTCCATC	60	273	NM_176644.2
	R: CCTAAATTCTGTTTTCCGTC			
*CYP17A1*	F: ATGGAAAAGATGAAGGGTT	60	106	NM_174304.2
	R: GCAGCAAGTTAGTGATGGA			
*CYP19A1*	F: GTAAGCTACTGAGAGTGGAAG	59	290	NM_174305.1
	R: GATGTATCTGTGTTGTCAGGTC			
*HIF-1α*	F: TGAAGGCACAGATGAATTGCTT	60	174	KU353607.1
	R: GTTCAAACTGAGTTAATCCCATGT			
*LC3-* *I*	F: GTAAAGAGGTGCAGCAGATC	60	143	NM_001046175.1
	R: GACCAACTCGCTCATGTTGAC			
*LC3-* *II*	F: GTCAACATGAGTGAGCTCATC	59	196	NM_001001169.1
	R: CGTATACCATATACAGGAATC			
*ATG5*	F: GATGAGATAACTGAACGCGAG	60	225	NM_001034579.2
	R: GTTCCTTGGAAGAGCTGAACT			
*BECN-1*	F: CCAACAGCTTCACTCTGATTGG	59	186	NM_001033627
	R: CAGTGACGTTGAGCTGAGTGTC			
*β-actin*	F: CTTCAACACCCCTGCCAT	60	238	JF830811
	R: CTCGGCTGTGGTGGTGAAG			

**Table 2:** Maturation rates of yak COCs cultured with different concentrations of rapamycin.

Treatment Groups	Total No. of Oocytes	Maturation Oocytes	Maturation Rates (% ± SEM)
Control	124	106	85.48 ± 2.26 ^b^
0.1 nM Rap	109	100	91.74 ± 4.65 ^a^
1.0 nM Rap	128	117	91.40 ± 0.86 ^a^
10 nM Rap	116	102	85.34 ± 1.58 ^b^
0.1 nM Rap + G15	126	95	75.40 ± 3.02 ^c^
0.1 nM Rap + PX-478	104	74	71.15 ± 1.80 ^d^

Maturation rates are shown as total numbers and as percentages of the total number of yak COCs in culture. The values indicate the mean standard error (MSE) of three independent experiments. Values with different superscripts in the same column are significantly different (*p* < 0.05).

**Table 3:** Effects of rapamycin on the parthenogenetic development of yak mature oocytes.

Treatment Groups	Total No. of Activated Oocyte	No. of Parthenogenetic Embryos at Different Stages			
		Two-to-Four Cell Embryos (%)	Four-to-Eight Cell Embryos (%)	Morula (%)	Blastocysts (%)
Control	106	82 (77.35 ± 0.64) ^b^	72 (67.92 ± 2.51) ^b^	60 (56.60 ± 4.52) ^b^	30 (28.32 ± 1.50) ^b^
0.1 nM Rap	100	82 (82.06 ± 2.04) ^a^	70 (70.52 ± 3.75) ^a^	59 (58.95 ± 3.16) ^a^	32 (32.05 ± 2.84) ^a^
0.1 nM Rap + G15	95	64 (67.37 ± 1.05) ^c^	50 (52.64 ± 1.38) ^c^	38 (40.02 ± 0.54) ^c^	20 (21.15 ± 2.63) ^c^
0.1 nM Rap + PX-478	74	49 (66.22 ± 5.16) ^c^	38 (51.40 ± 2.62) ^d^	29 (39.18 ± 3.06) ^c^	15 (20.26 ± 1.86) ^c^

The rates of parthenogenetic embryos at different stages were calculated from the number of mature oocytes. Statistical significance (*p* < 0.05) between the values is indicated with different superscripts in the same column.

## Data Availability

All data presented in this study are available upon request from the corresponding authors.

## Supplementary Materials

The following supporting information can be downloaded at: https://www.mdpi.com/article/10.3390/ani15030365/s1, Full western blots are provided in the Supplementary Materials File S1.
