# Supplementary material for: Rapamycin Increases the Development Competence of Yak (Bos grunniens) Oocytes by Promoting Autophagy via Upregulating 17β-Estradiol and HIF-1α During In Vitro Maturation
Source: Animals (Basel). 2025 Jan 27;15(3):365. doi: 10.3390/ani15030365 (PMC11816318; doi:10.3390/ani15030365)
Supplement: Supplementary file 1 [file animals-15-00365-s001.zip › Table S1.docx]

**Table S1.**Antibodies, suppliers, and dilutions used for immunohistochemistry and western blotting.

| **Antibodies** | | | **Originated** | **Dilution used** | |
| --- | --- | --- | --- | --- | --- |
|  | |  |  | **IF** | **WB** |
| **Primary** | CYP19A1 | | Rabbit polyclonal (ab18995, Abcam, Cambridge, UK) | 1:500 | 1:100 |
|  | CYP17A1 | | Goat polyclonal (ab48019, Abcam, Cambridge, UK) | 1:400 | 1:50 |
|  | CYP11A1 | | Goat polyclonal (ab272494, Abcam, Cambridge, UK) | 1:300 |  |
|  | HIF-1α | | Mouse monoclonal (ab16066, Abcam, Cambridge, UK) | 1:100 | 1:500 |
|  | ATG5 | | Rabbit polyclonal (ab228668, Abcam, Cambridge, UK) | 1:100 | 1:500 |
|  | BECN1 | | Mouse monoclonal (ab114071, Abcam, Cambridge, UK) | 1:200 | 1:500 |
|  | LC3 | | Rabbit polyclonal (ab51520, Abcam, Cambridge, UK) | 1:1000 | 1:1000 |
|  | β-actin | | Rabbit polyclonal (ab8227, Abcam, Cambridge, UK) |  | 1:1000 |
| **Secondary** | Mouse Anti-Rabbit IgG H&L/HRP | | (bs-0295M-HRP, Bioss, Beijing, China) |  | 1:5000(CYP19A1, ATG5, LC3, β-actin) |
|  | Goat Anti-Mouse IgG H&L/HRP | | (bs-40296G-HRP, Bioss, Beijing, China) |  | 1:5000(HIF-1α, BECN1) |
|  | Mouse Anti-Goat IgG H&L/HRP | | (bs-0294M-HRP-HRP, Bioss, Beijing, China) |  | 1:5000(CYP17A1) |
|  | Mouse Anti-Rabbit IgG H&L/AF488 | | (bs-0295M-AF488, Bioss, Beijing, China) | 1:800(CYP19A1, ATG5) |  |
|  | Rabbit Anti-Mouse IgG Fc/AF488 | | (bs-0377R-AF488, Bioss, Beijing, China) | 1:800(HIF-1α) |  |
|  | Rabbit Anti-Mouse IgG Fc/FITC | | (bs-0377R-FITC, Bioss, Beijing, China) | 1:800(BECN1) |  |
|  | Rabbit Anti-Goat IgG H& /FITC | | (bs-0294R-FITC, Bioss, Beijing, China) | 1:800(CYP17A1) |  |
