# Supplementary figures and images for: Rapamycin Increases the Development Competence of Yak (Bos grunniens) Oocytes by Promoting Autophagy via Upregulating 17β-Estradiol and HIF-1α During In Vitro Maturation
Source: Animals (Basel). 2025 Jan 27;15(3):365. doi: 10.3390/ani15030365 (PMC11816318; doi:10.3390/ani15030365)

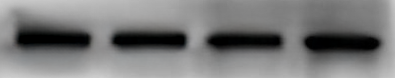

Supplement: Supplementary file 1 [file animals-15-00365-s001.zip › S1/WB original-241221/Fig2/Fig.2 CYP11A1-原图1.png]

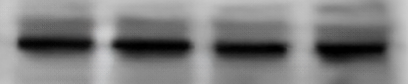

Supplement: Supplementary file 1 [file animals-15-00365-s001.zip › S1/WB original-241221/Fig2/Fig.2 CYP11A1-原图2 - 副本.png]

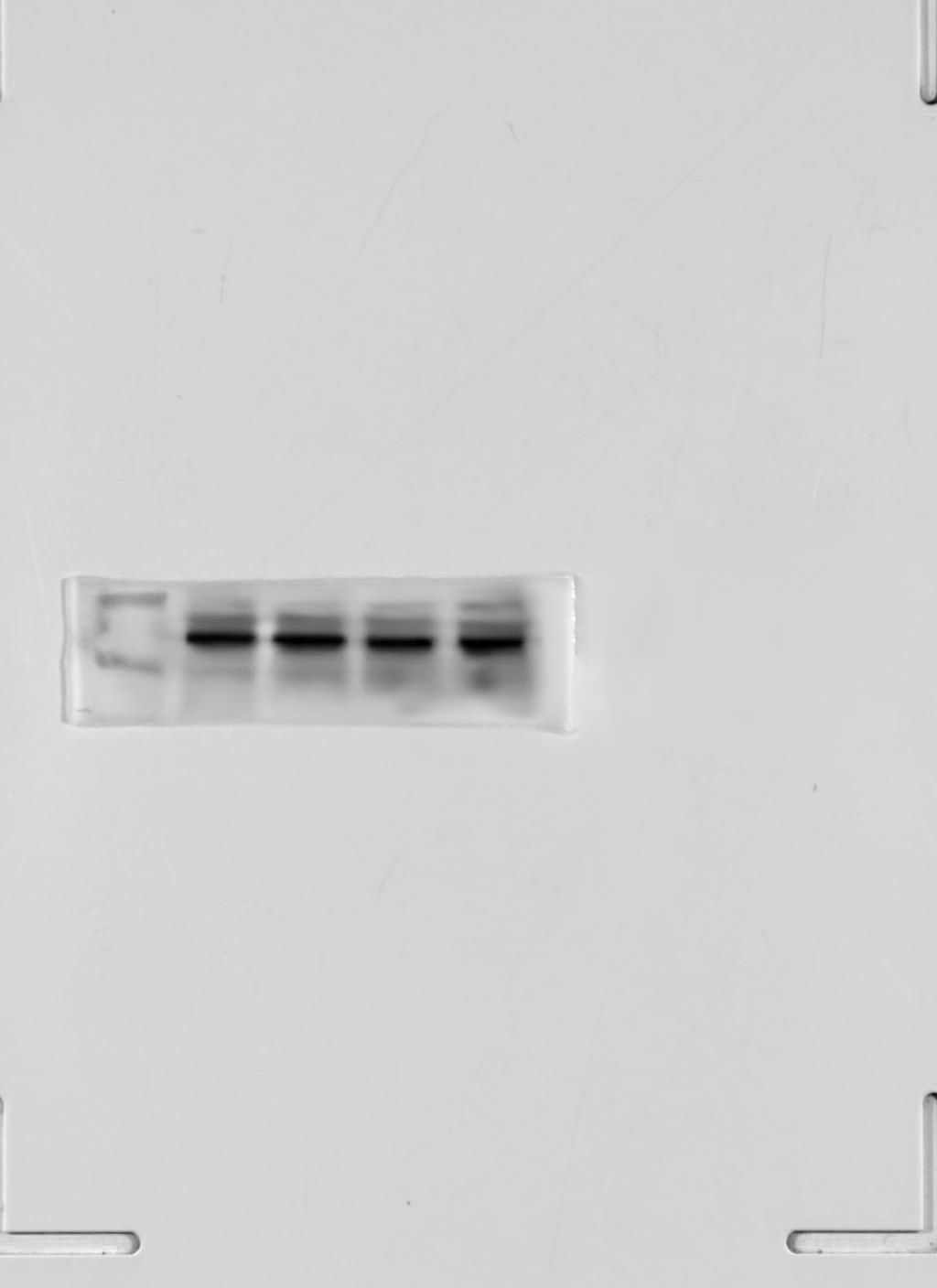

Supplement: Supplementary file 1 [file animals-15-00365-s001.zip › S1/WB original-241221/Fig2/Fig.2 CYP11A1-原图2.png]

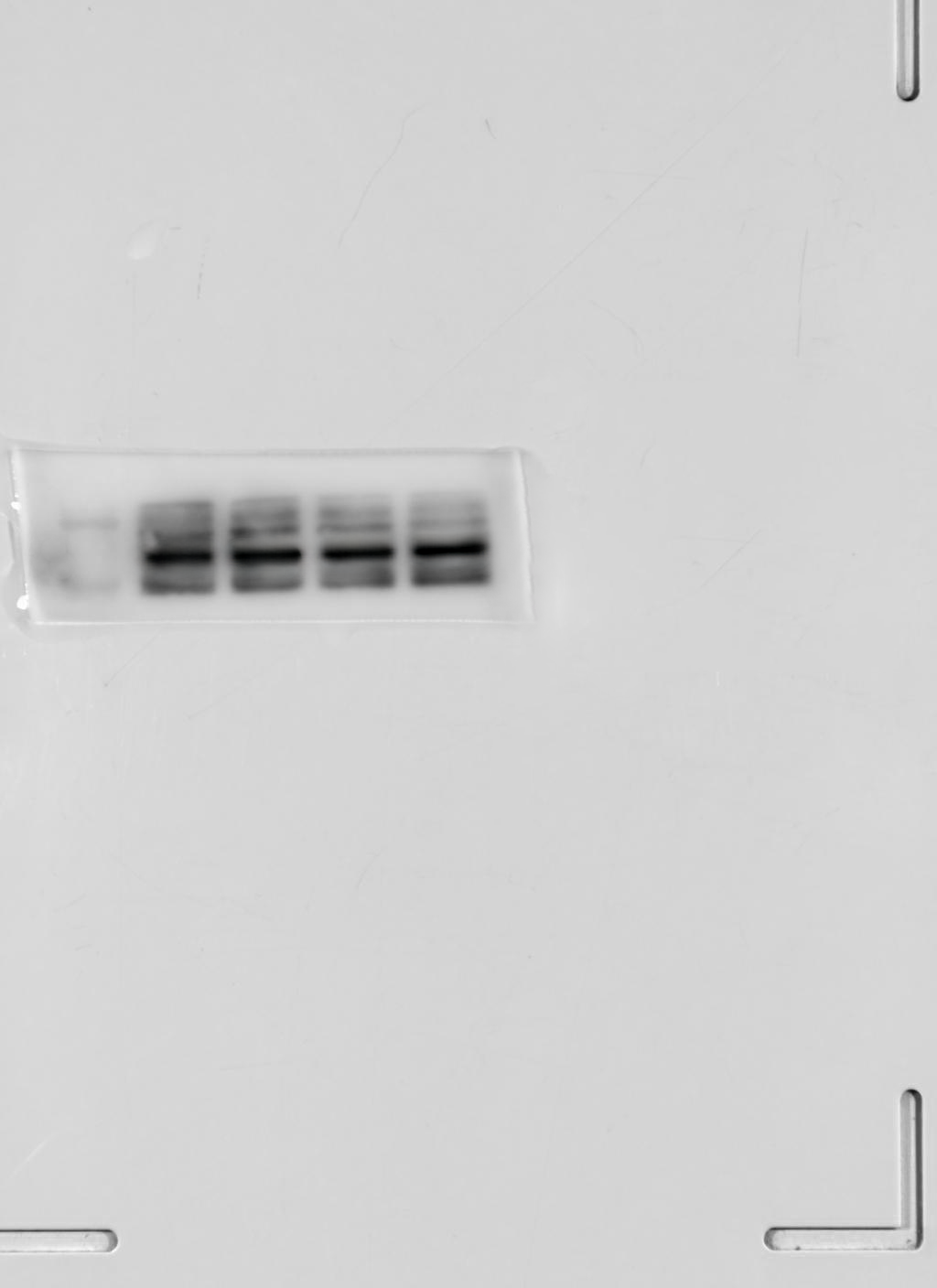

Supplement: Supplementary file 1 [file animals-15-00365-s001.zip › S1/WB original-241221/Fig2/Fig.2 CYP11A1-原图3.png]

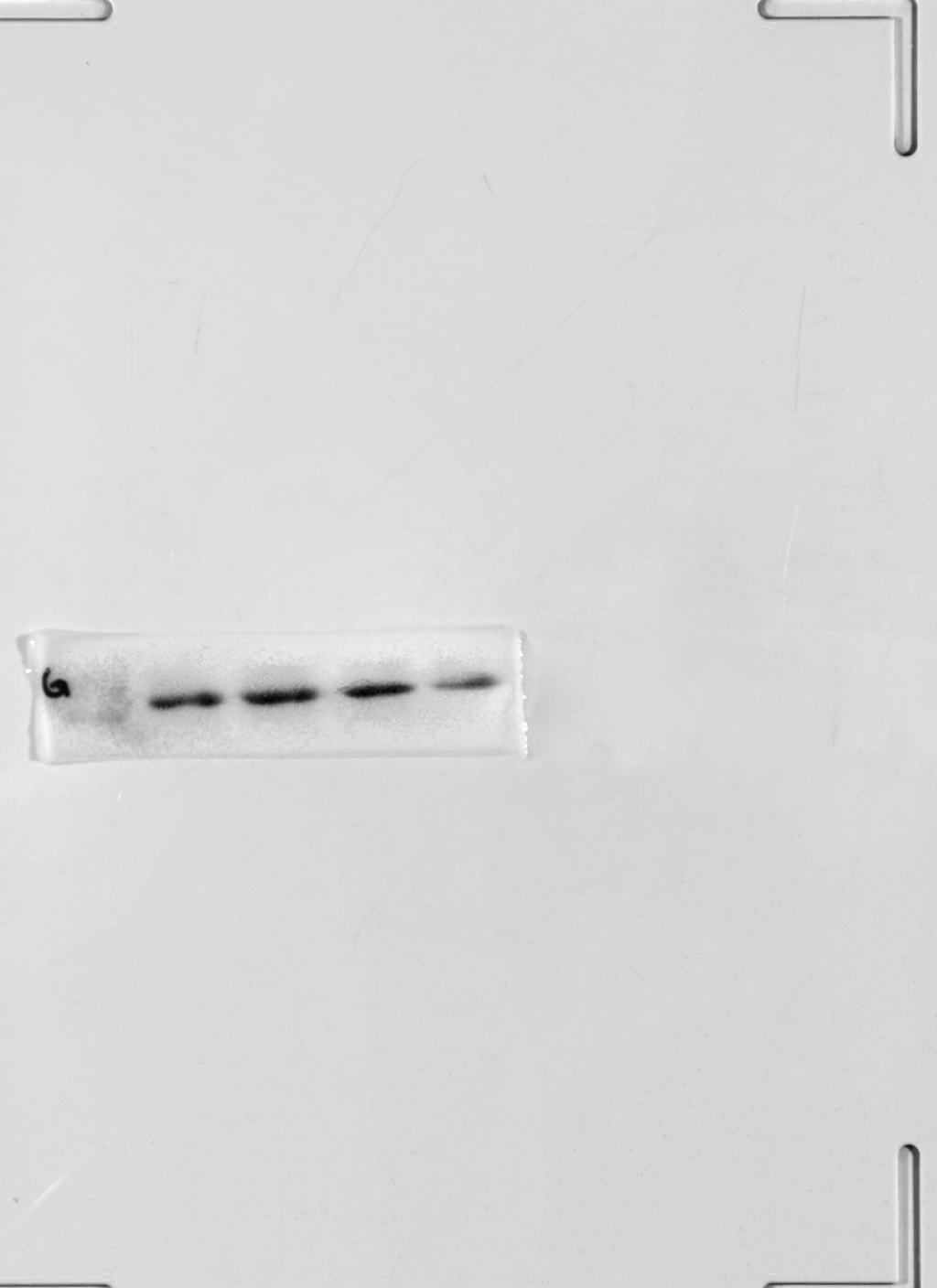

Supplement: Supplementary file 1 [file animals-15-00365-s001.zip › S1/WB original-241221/Fig2/Fig.2 CYP17A1-原图1.png]

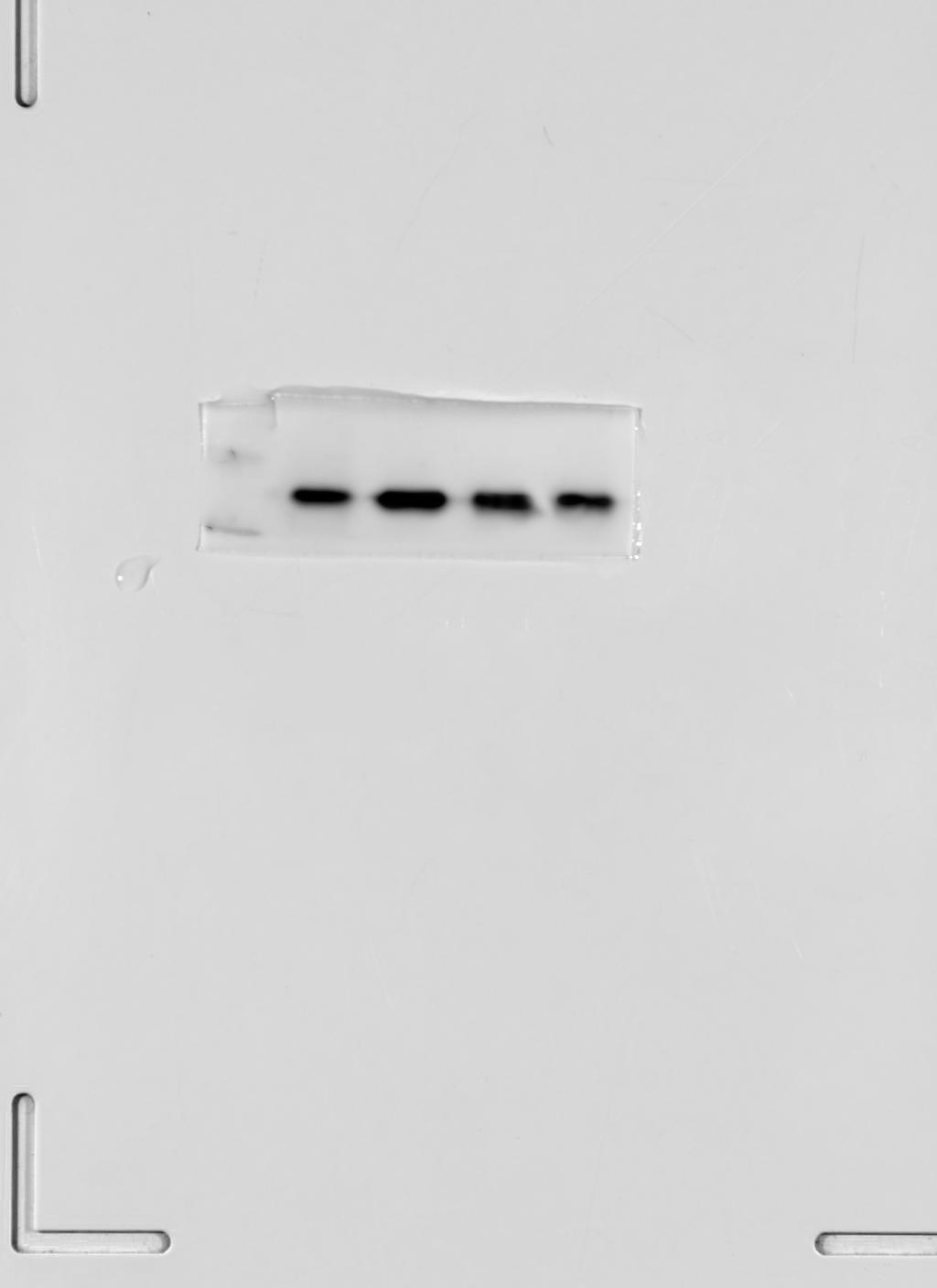

Supplement: Supplementary file 1 [file animals-15-00365-s001.zip › S1/WB original-241221/Fig2/Fig.2 CYP17A1-原图2.png]

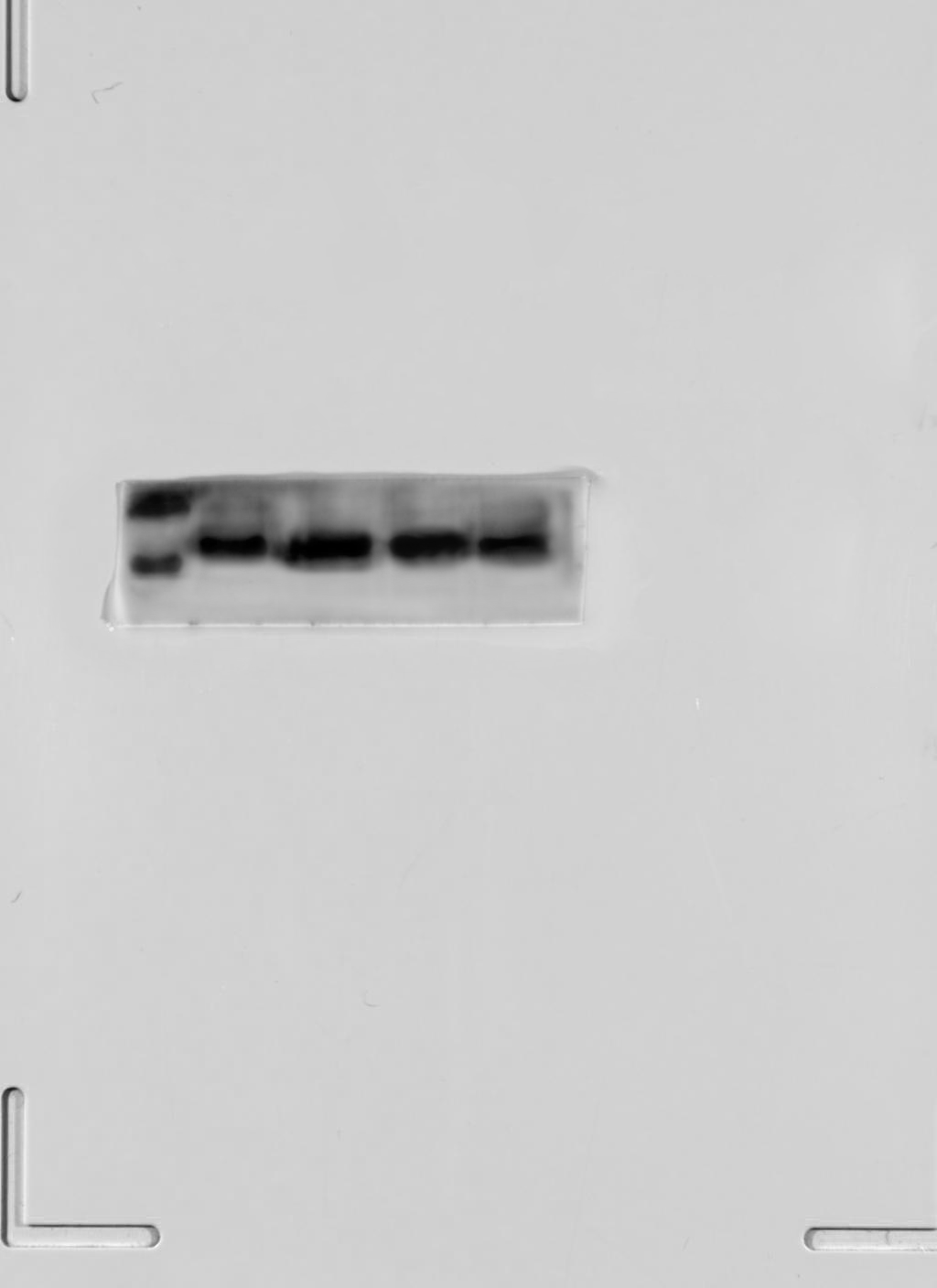

Supplement: Supplementary file 1 [file animals-15-00365-s001.zip › S1/WB original-241221/Fig2/Fig.2 CYP17A1-原图3.png]

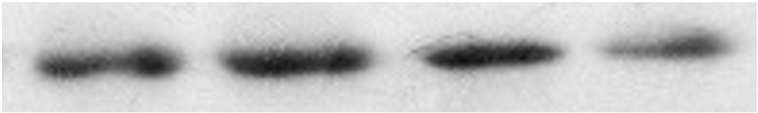

Supplement: Supplementary file 1 [file animals-15-00365-s001.zip › S1/WB original-241221/Fig2/Fig.2 CYP17A1.png]

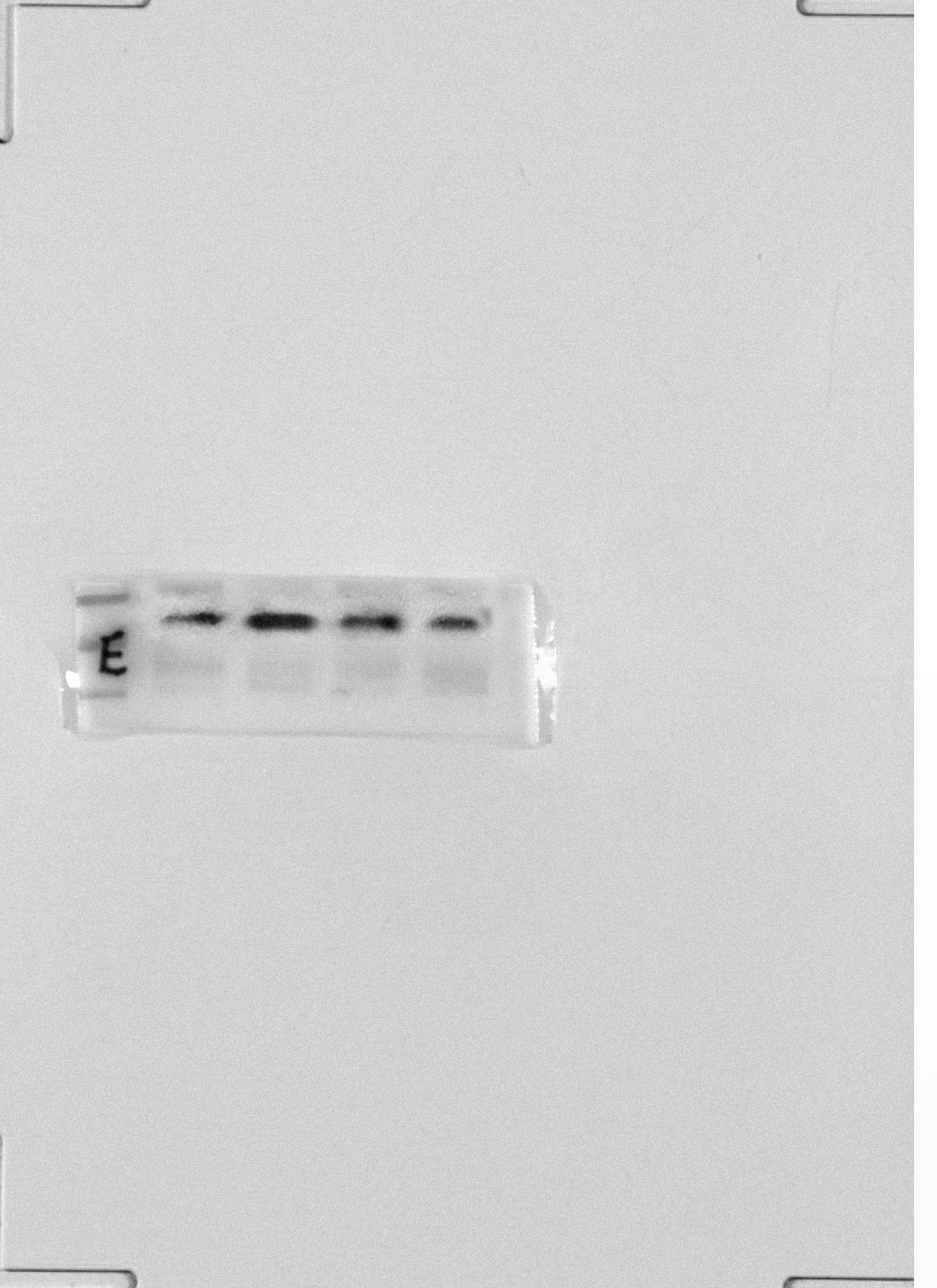

Supplement: Supplementary file 1 [file animals-15-00365-s001.zip › S1/WB original-241221/Fig2/Fig.2 CYP19A1-原图1.png]

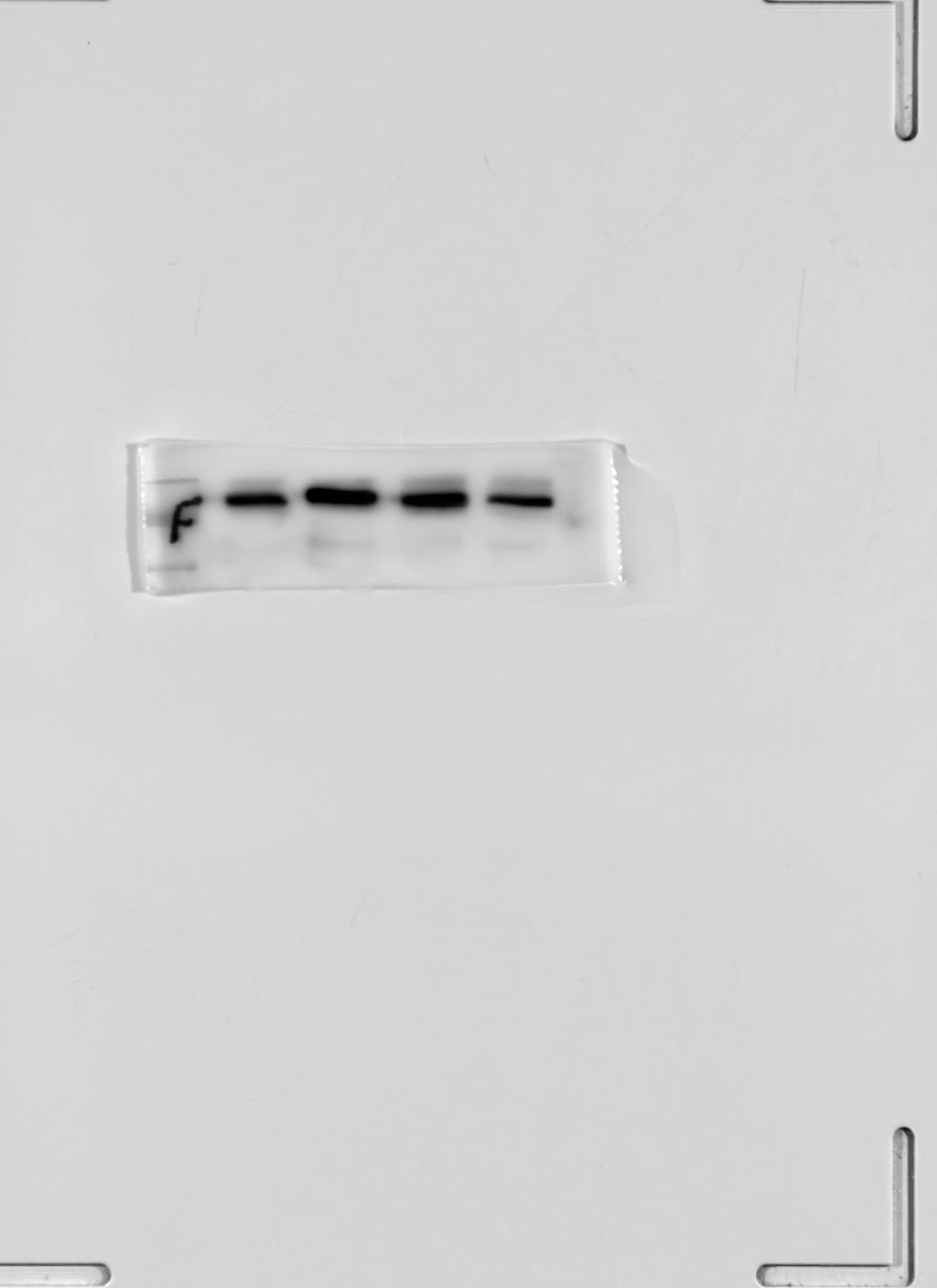

Supplement: Supplementary file 1 [file animals-15-00365-s001.zip › S1/WB original-241221/Fig2/Fig.2 CYP19A1-原图2.png]

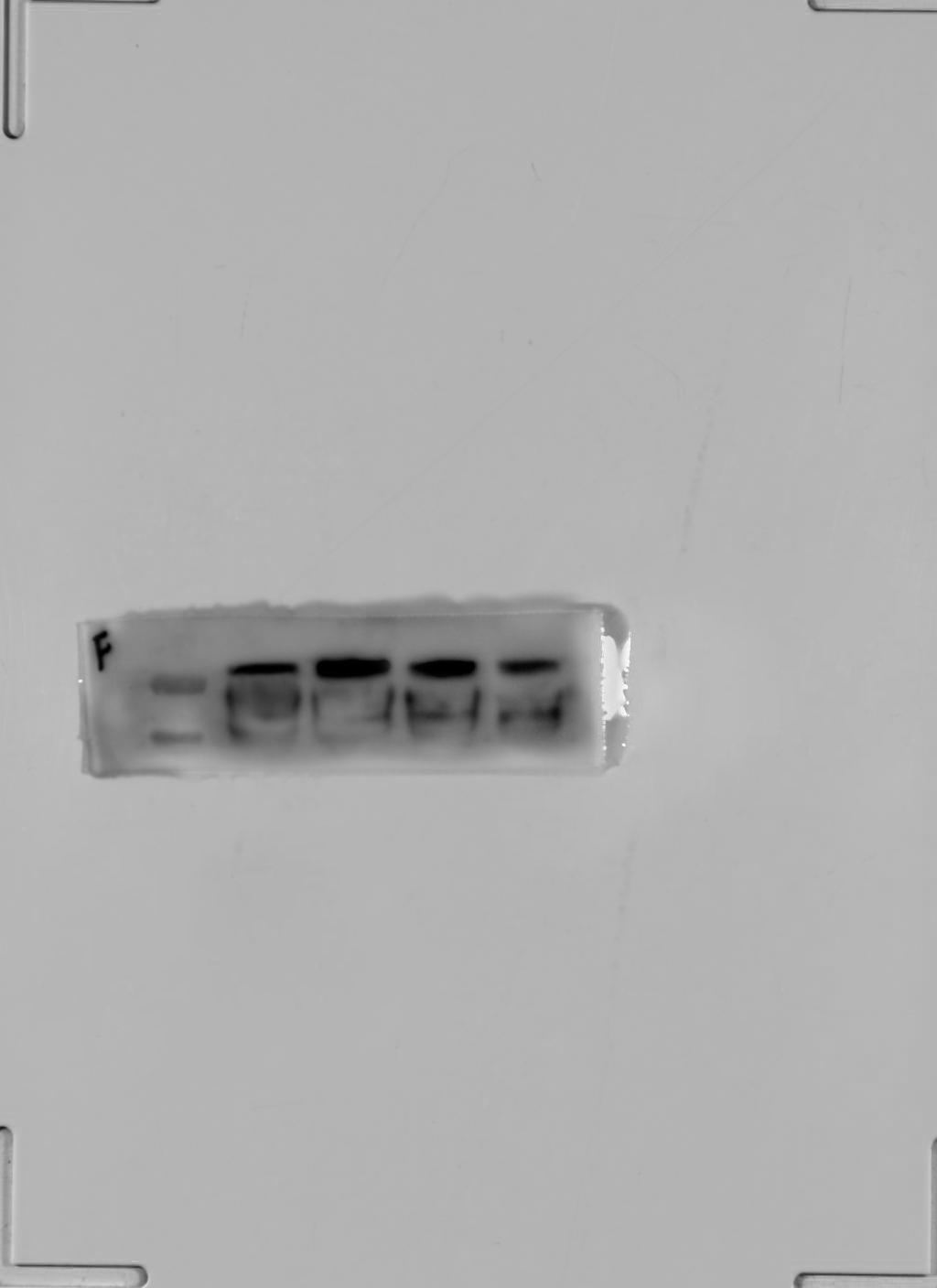

Supplement: Supplementary file 1 [file animals-15-00365-s001.zip › S1/WB original-241221/Fig2/Fig.2 CYP19A1-原图3.png]

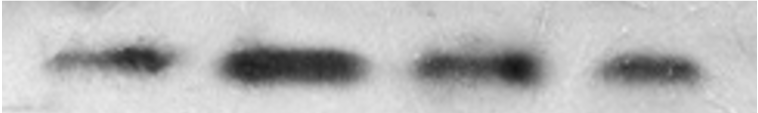

Supplement: Supplementary file 1 [file animals-15-00365-s001.zip › S1/WB original-241221/Fig2/Fig.2 CYP19A1.png]

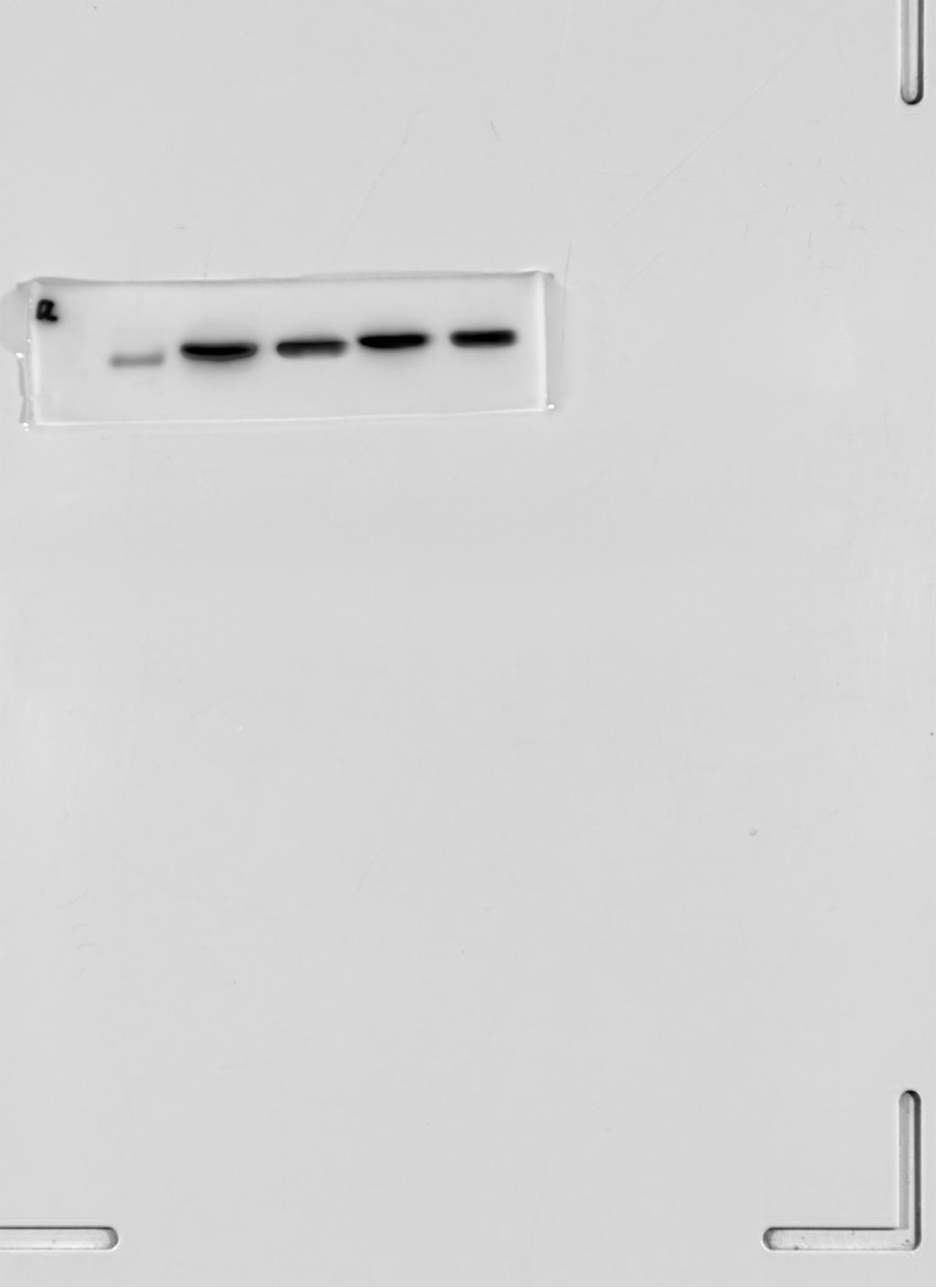

Supplement: Supplementary file 1 [file animals-15-00365-s001.zip › S1/WB original-241221/Fig2/Fig.2 β-actin-原图1.png]

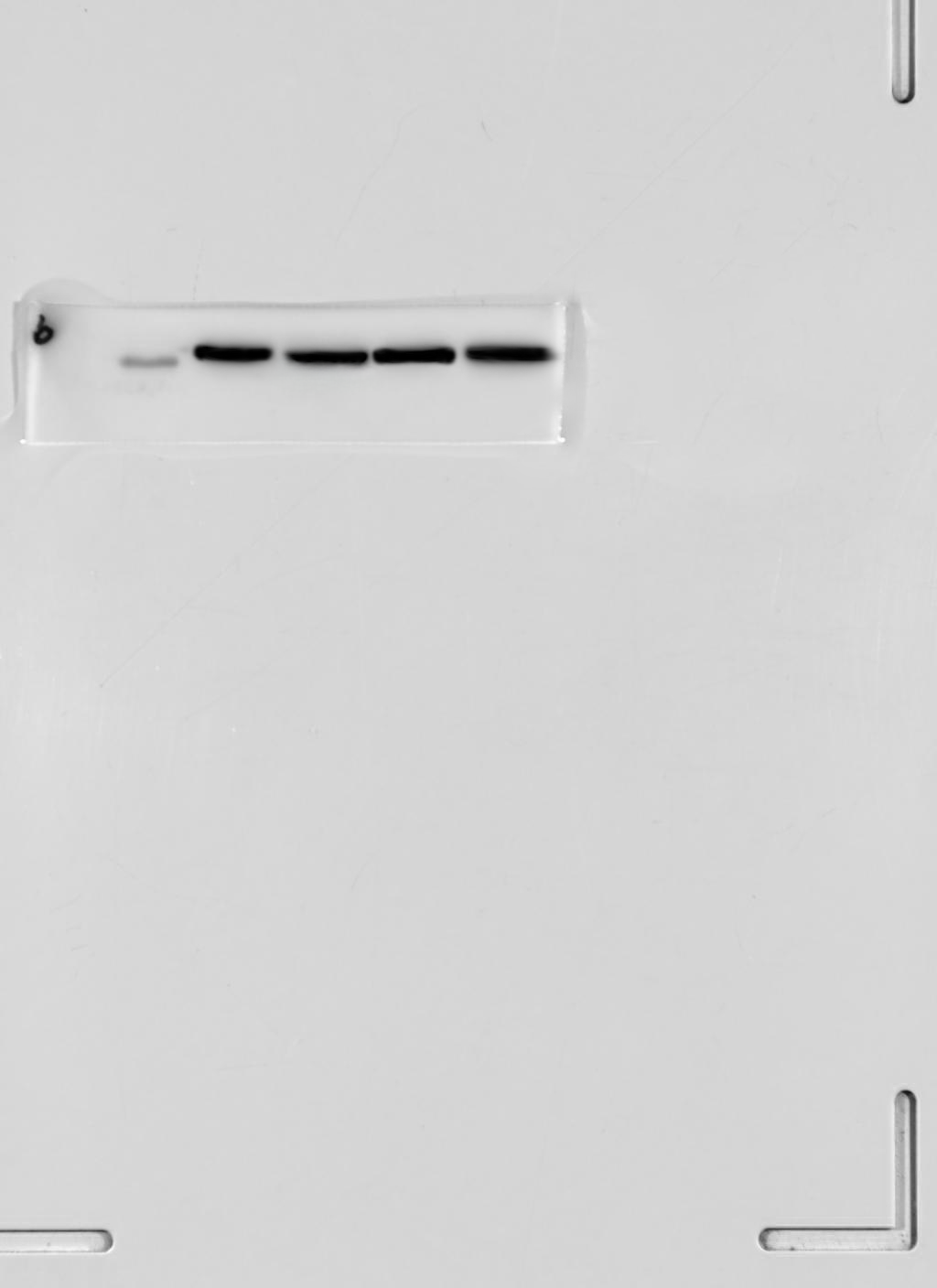

Supplement: Supplementary file 1 [file animals-15-00365-s001.zip › S1/WB original-241221/Fig2/Fig.2 β-actin-原图2.png]

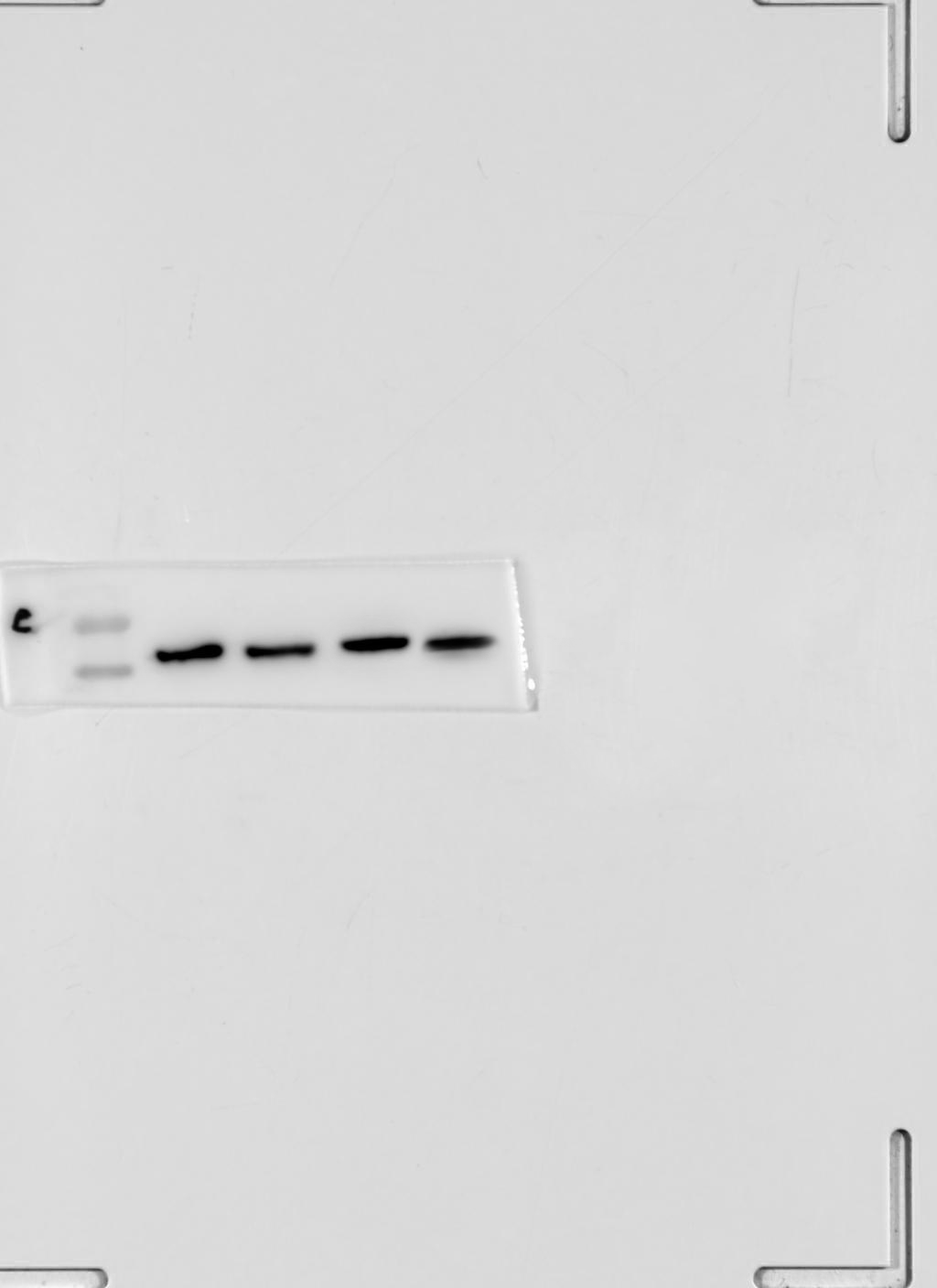

Supplement: Supplementary file 1 [file animals-15-00365-s001.zip › S1/WB original-241221/Fig2/Fig.2 β-actin-原图3.png]

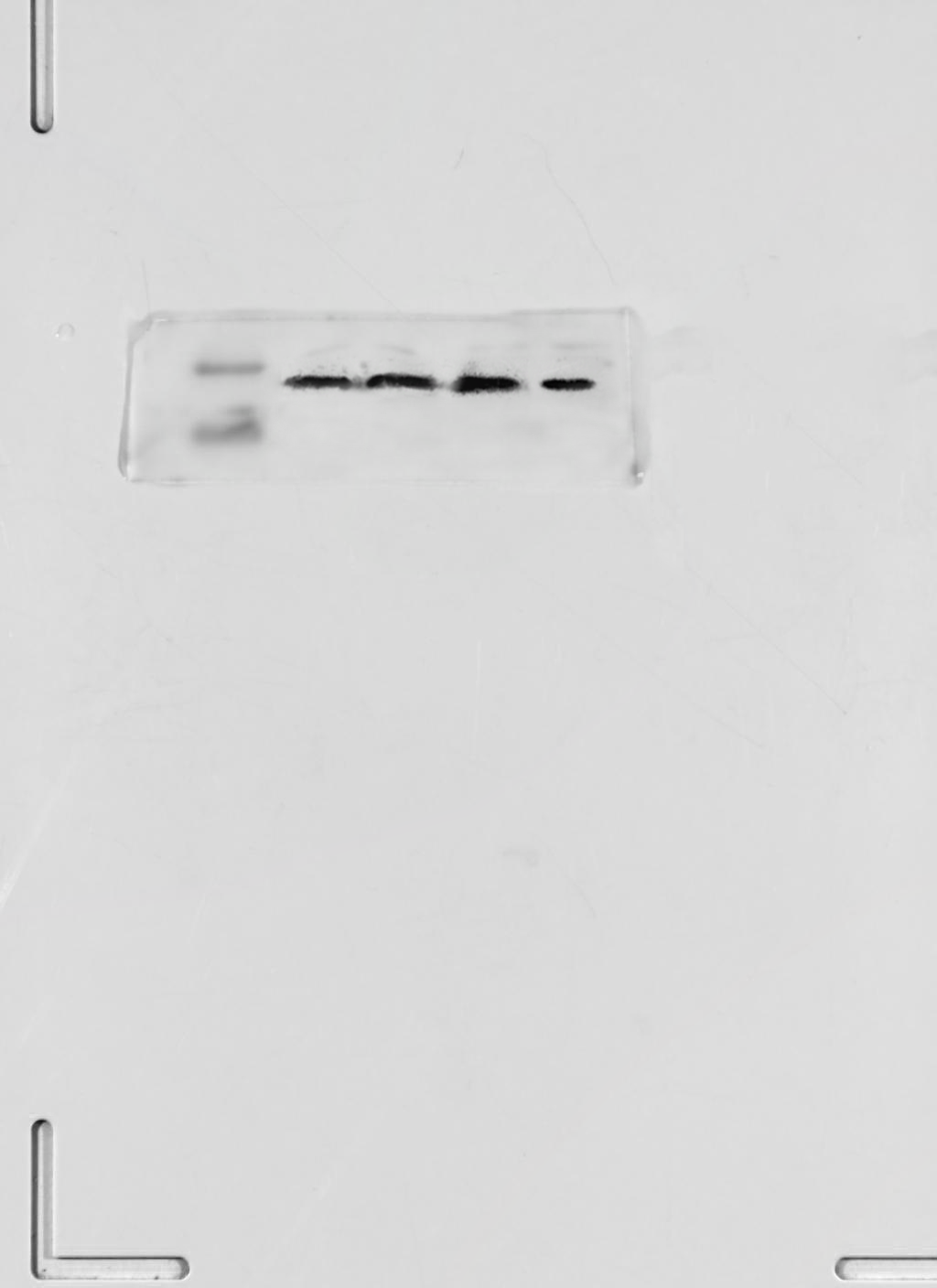

Supplement: Supplementary file 1 [file animals-15-00365-s001.zip › S1/WB original-241221/Fig3/Fig.3 HIF-1a-原图1.png]

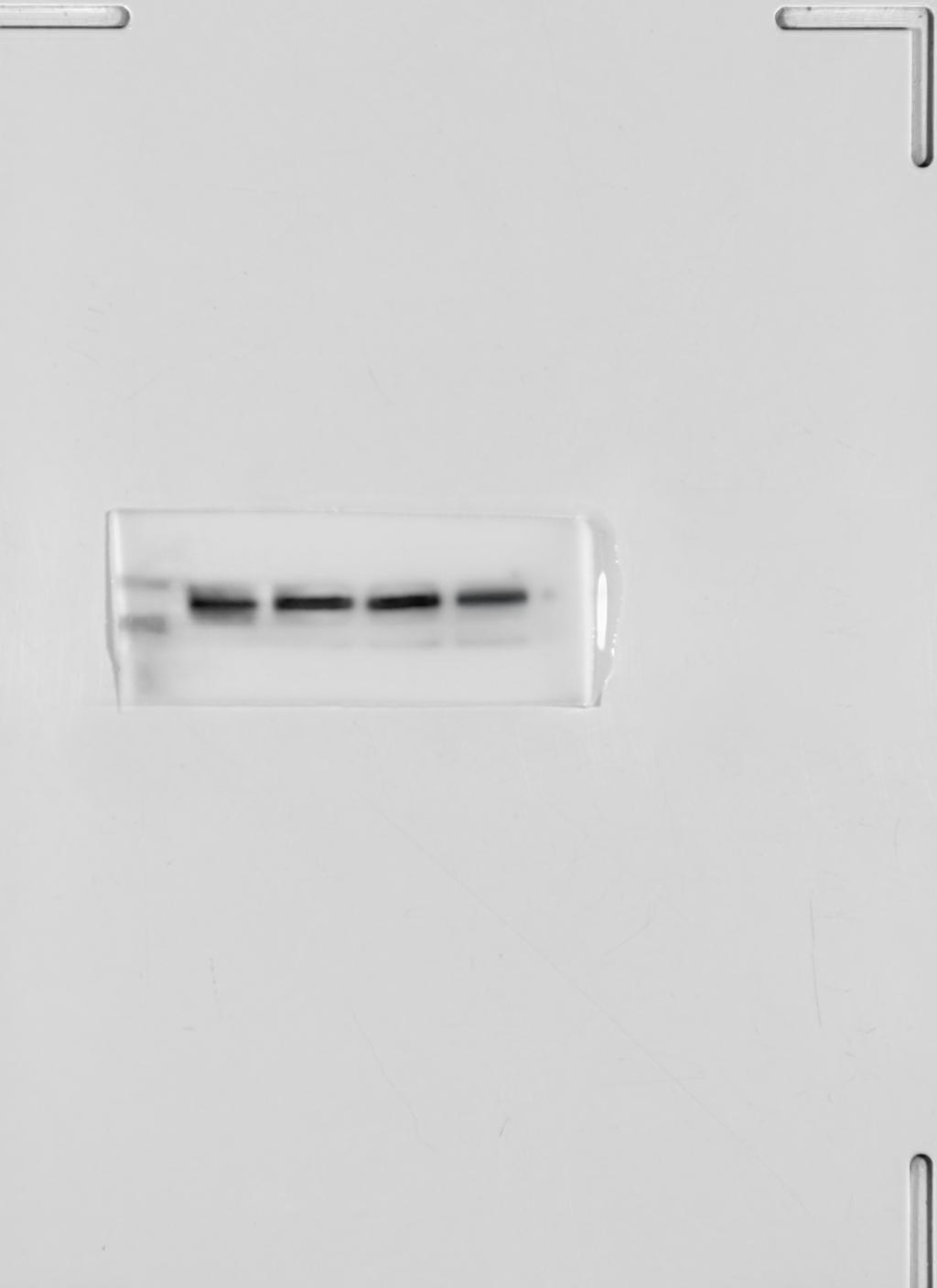

Supplement: Supplementary file 1 [file animals-15-00365-s001.zip › S1/WB original-241221/Fig3/Fig.3 HIF-1a-原图2.png]

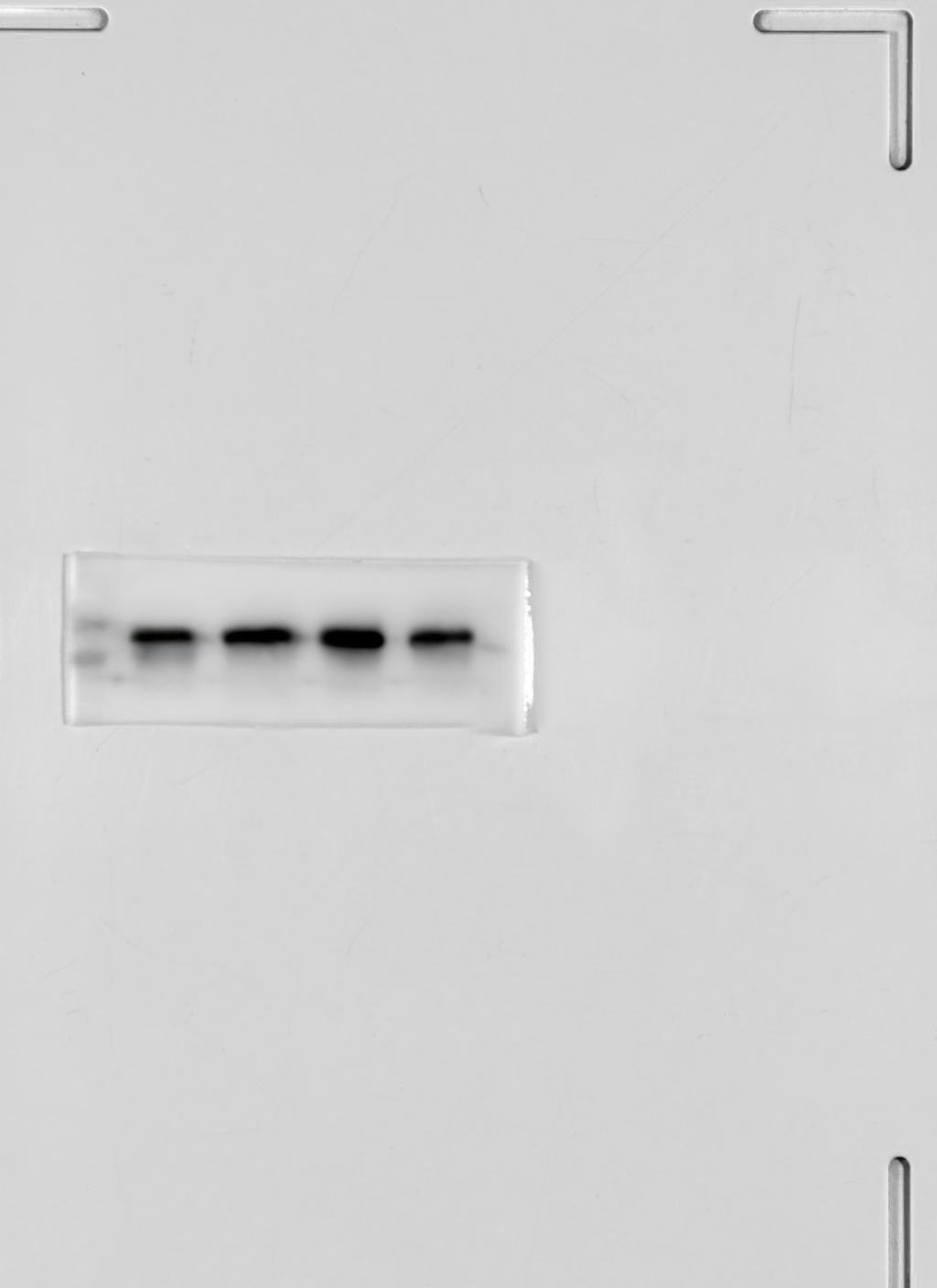

Supplement: Supplementary file 1 [file animals-15-00365-s001.zip › S1/WB original-241221/Fig3/Fig.3 HIF-1a-原图3.png]

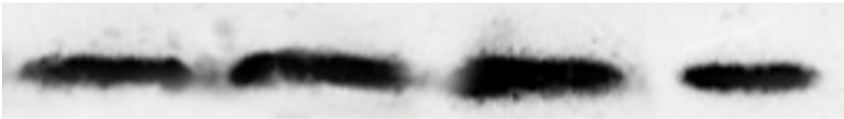

Supplement: Supplementary file 1 [file animals-15-00365-s001.zip › S1/WB original-241221/Fig3/Fig.3 HIF-1a.png]

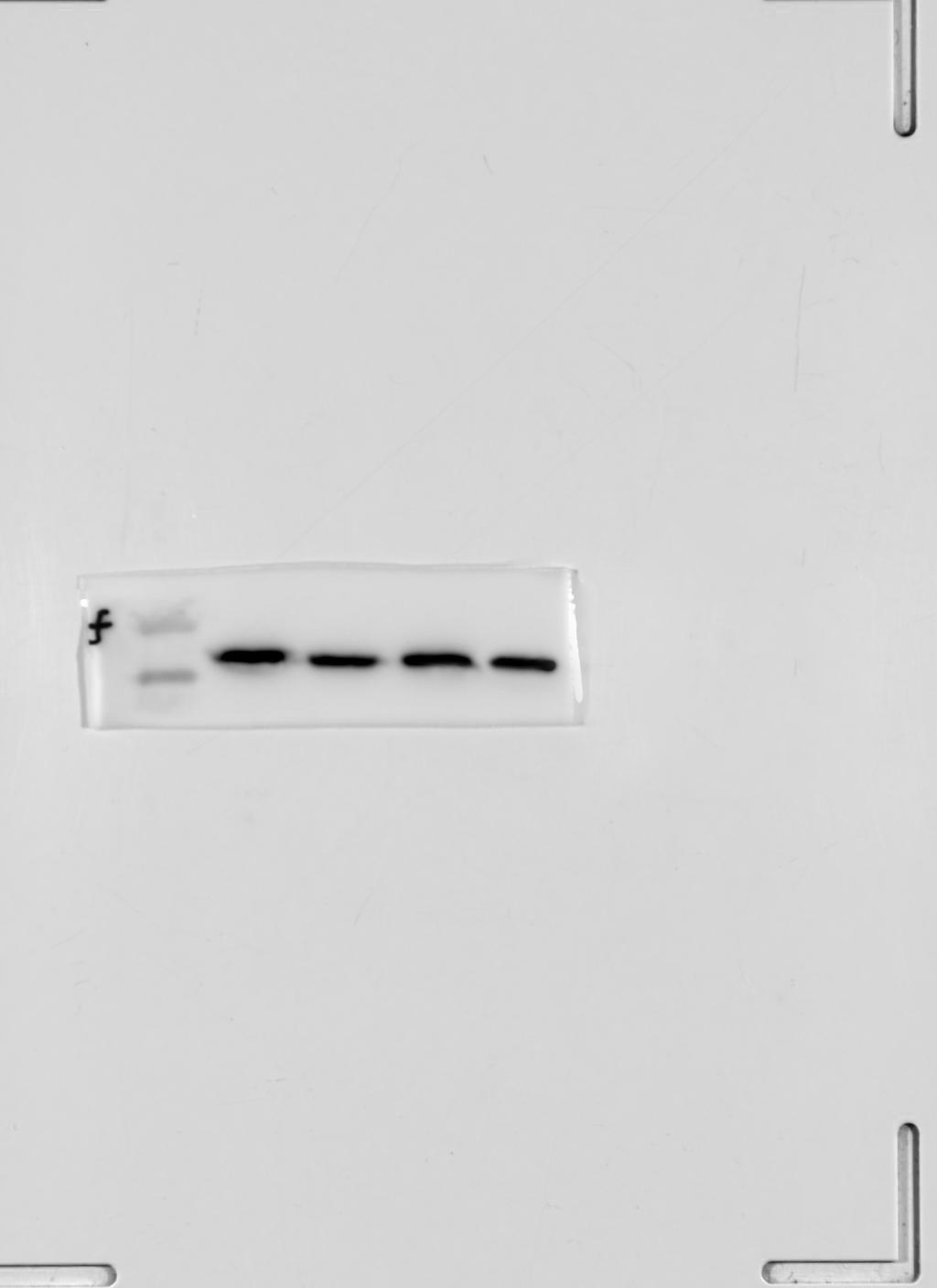

Supplement: Supplementary file 1 [file animals-15-00365-s001.zip › S1/WB original-241221/Fig3/Fig.3 β-actin-原图1.png]

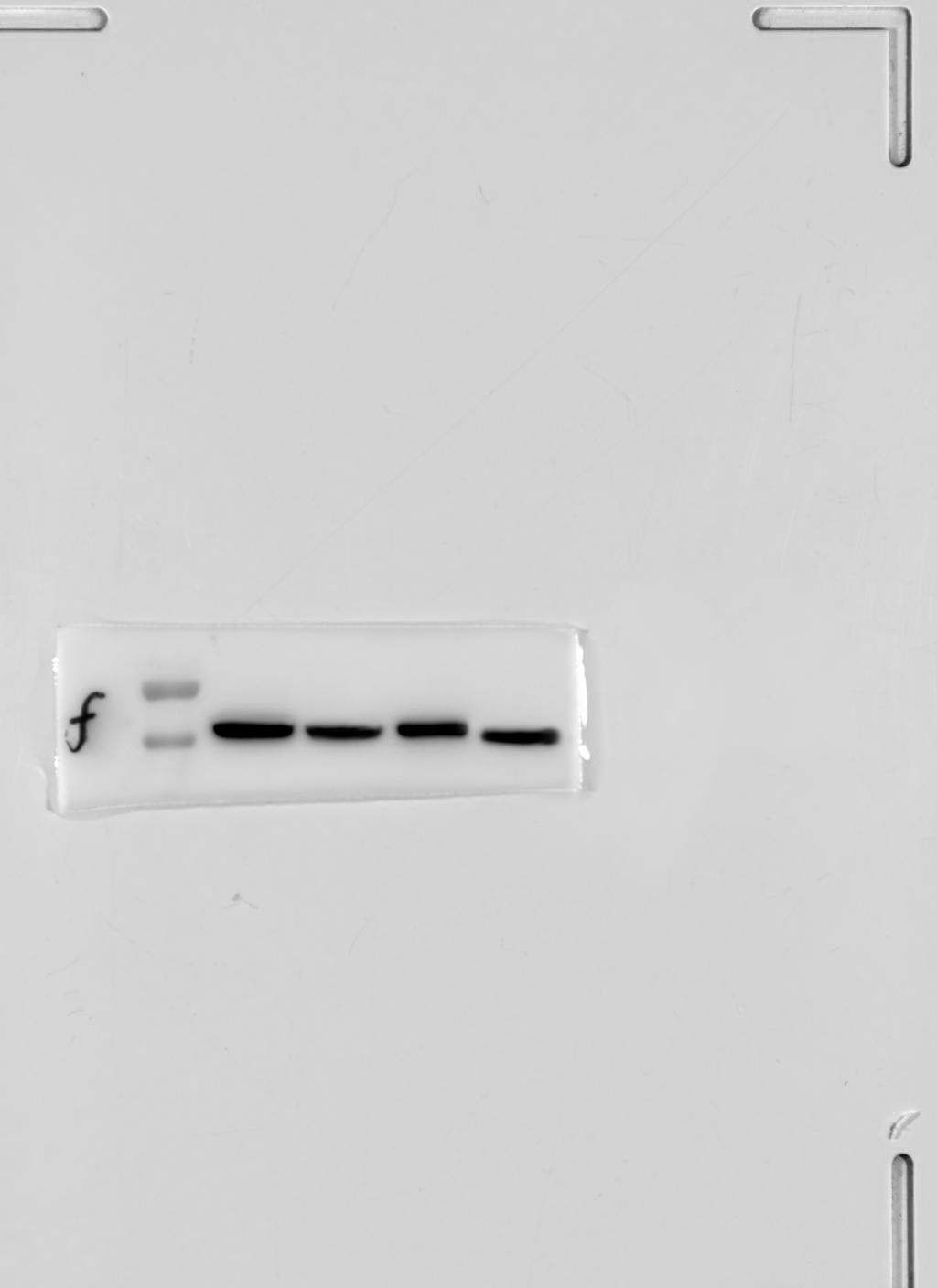

Supplement: Supplementary file 1 [file animals-15-00365-s001.zip › S1/WB original-241221/Fig3/Fig.3 β-actin-原图2.png]

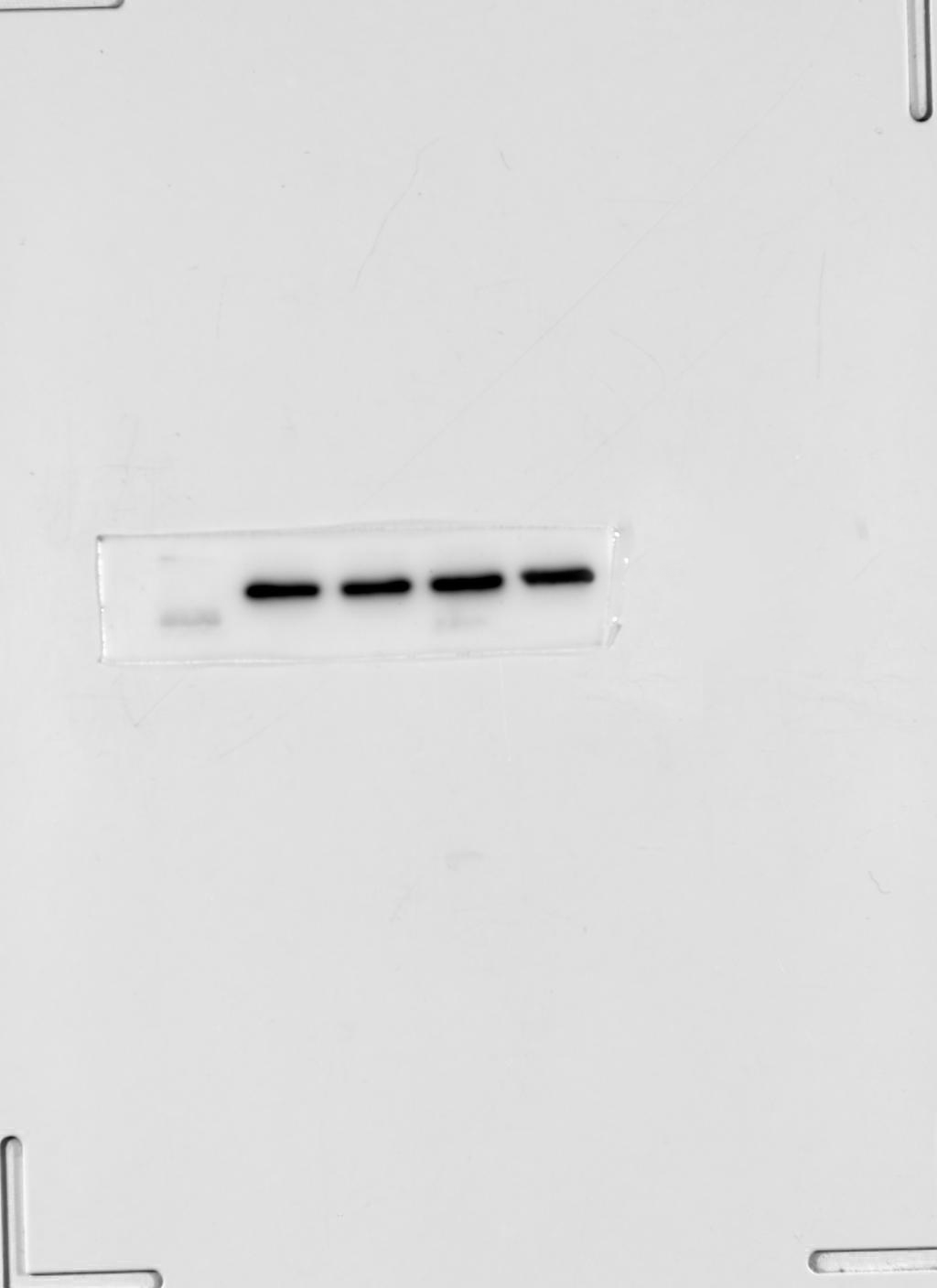

Supplement: Supplementary file 1 [file animals-15-00365-s001.zip › S1/WB original-241221/Fig3/Fig.3 β-actin-原图3.png]

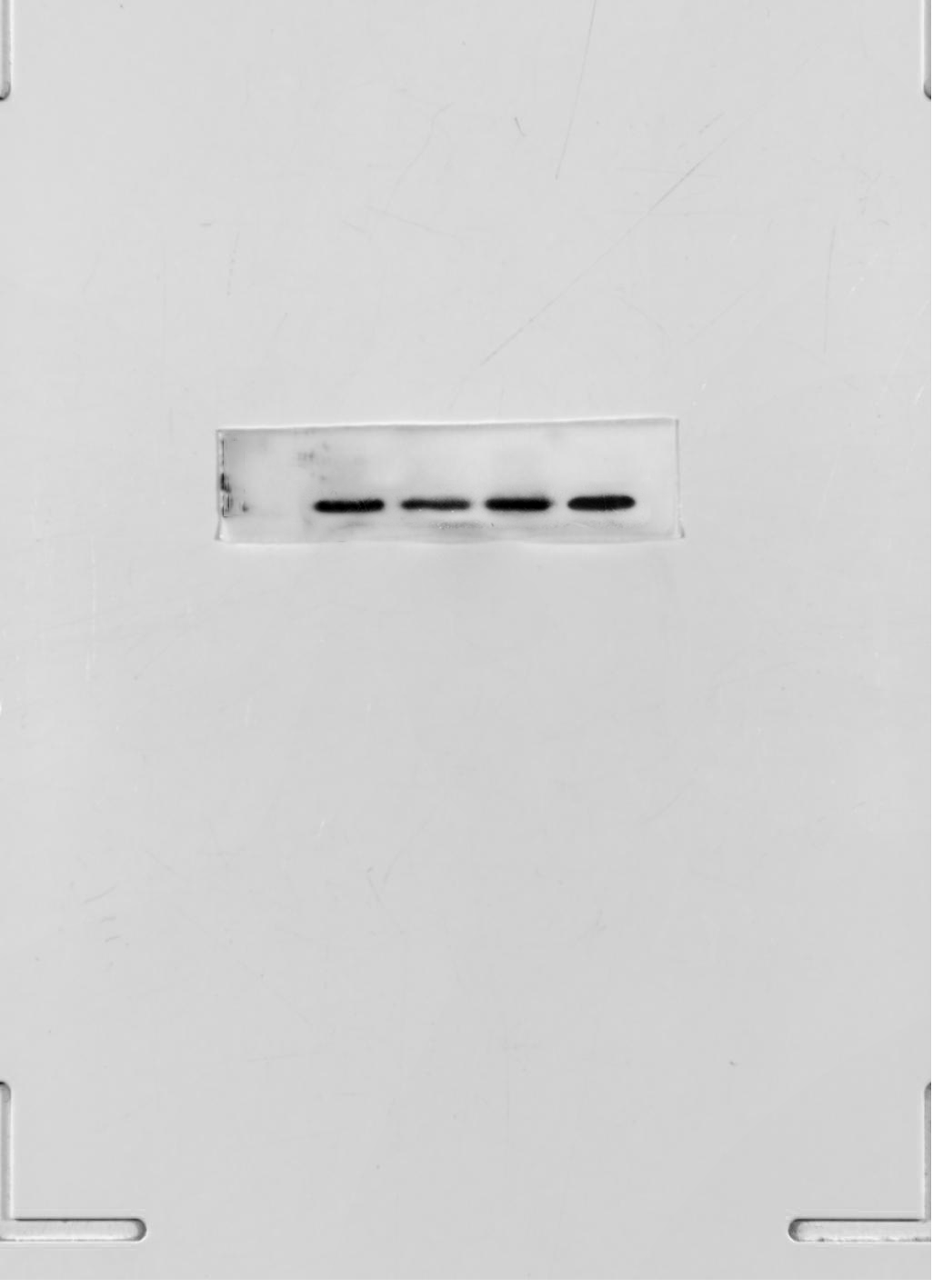

Supplement: Supplementary file 1 [file animals-15-00365-s001.zip › S1/WB original-241221/Fig4/Fig.4 actin-原图1.png]

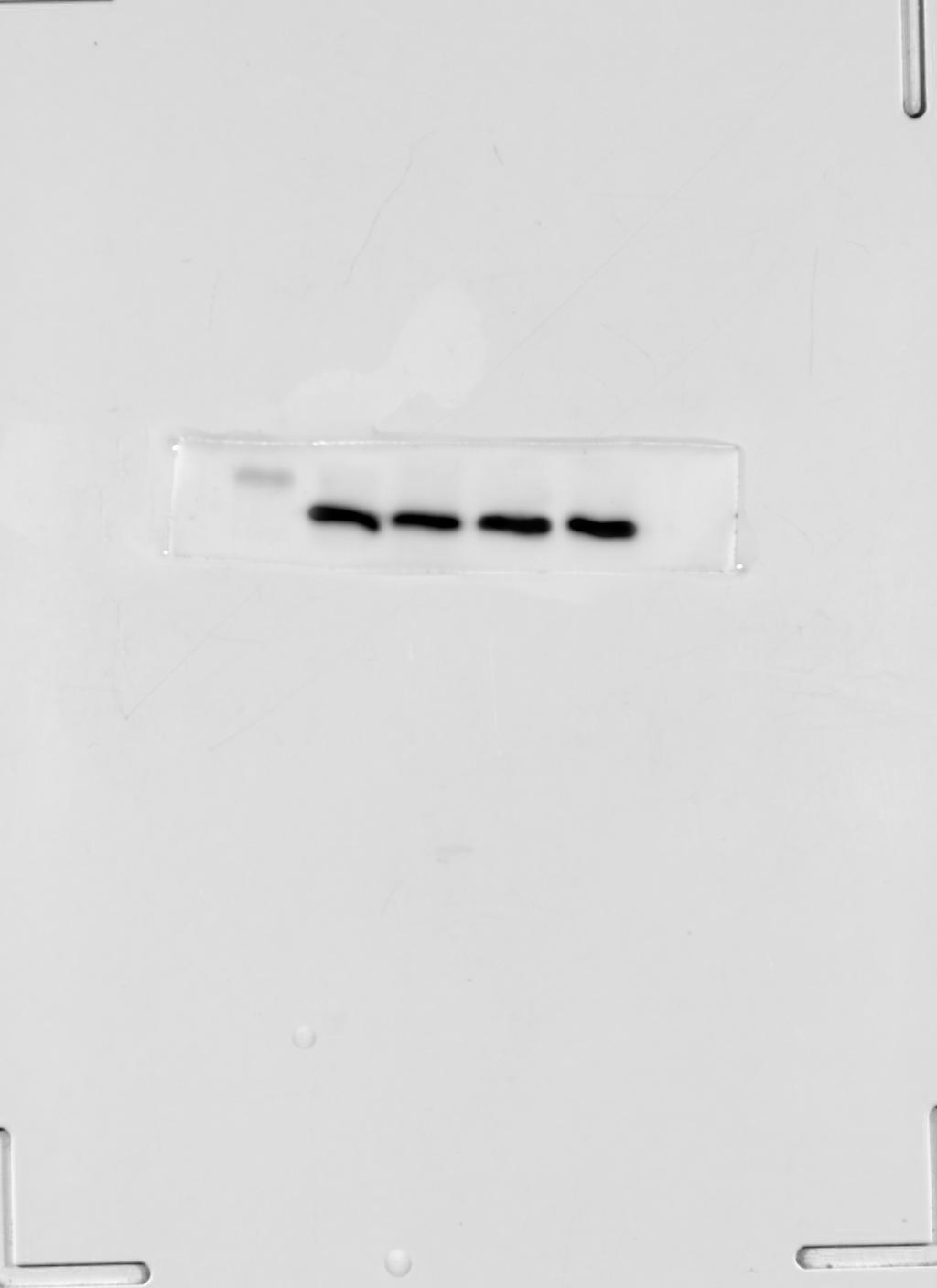

Supplement: Supplementary file 1 [file animals-15-00365-s001.zip › S1/WB original-241221/Fig4/Fig.4 actin-原图2.png]

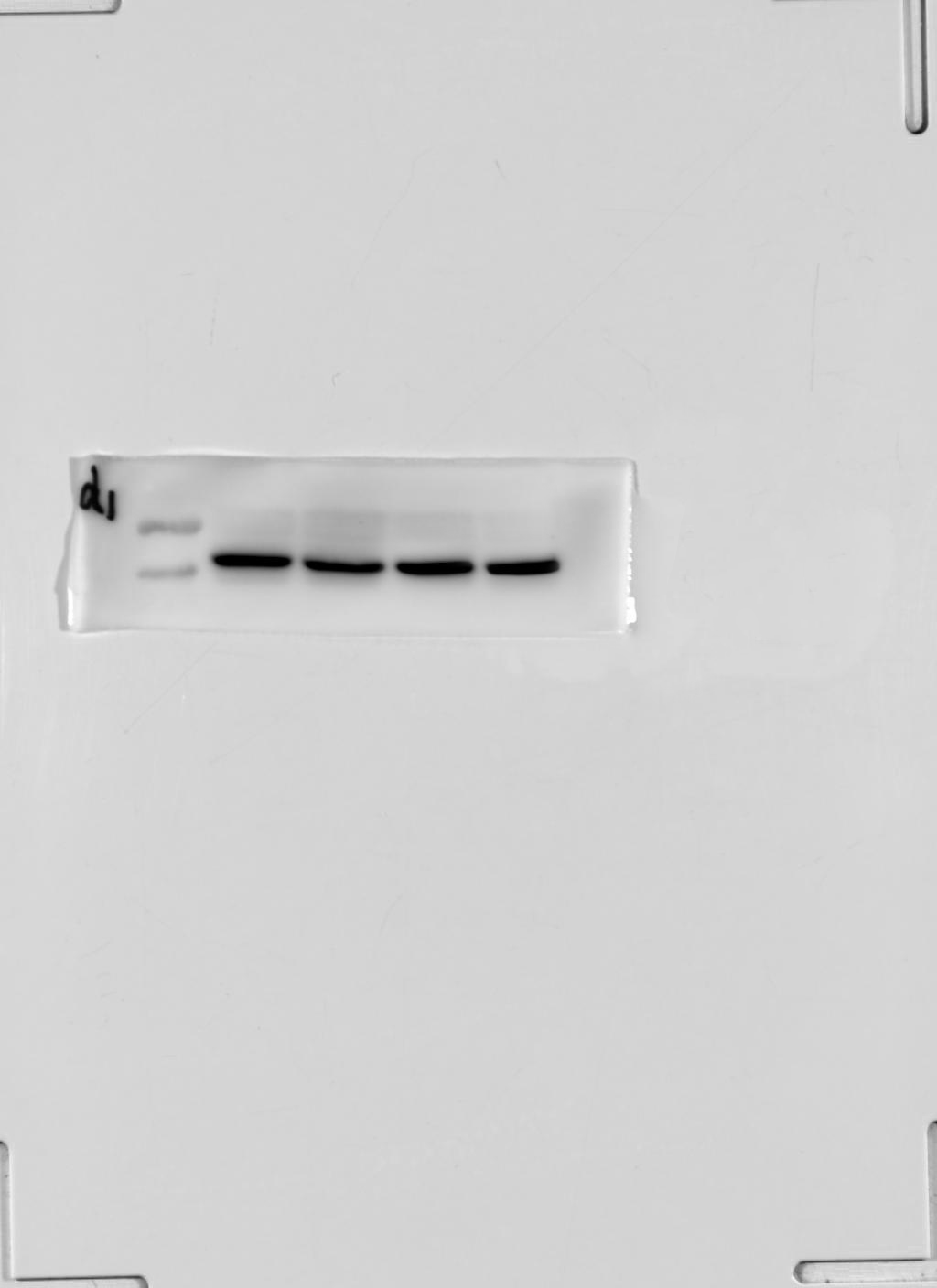

Supplement: Supplementary file 1 [file animals-15-00365-s001.zip › S1/WB original-241221/Fig4/Fig.4 actin-原图3.png]

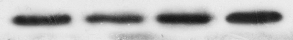

Supplement: Supplementary file 1 [file animals-15-00365-s001.zip › S1/WB original-241221/Fig4/Fig.4 actin.png]

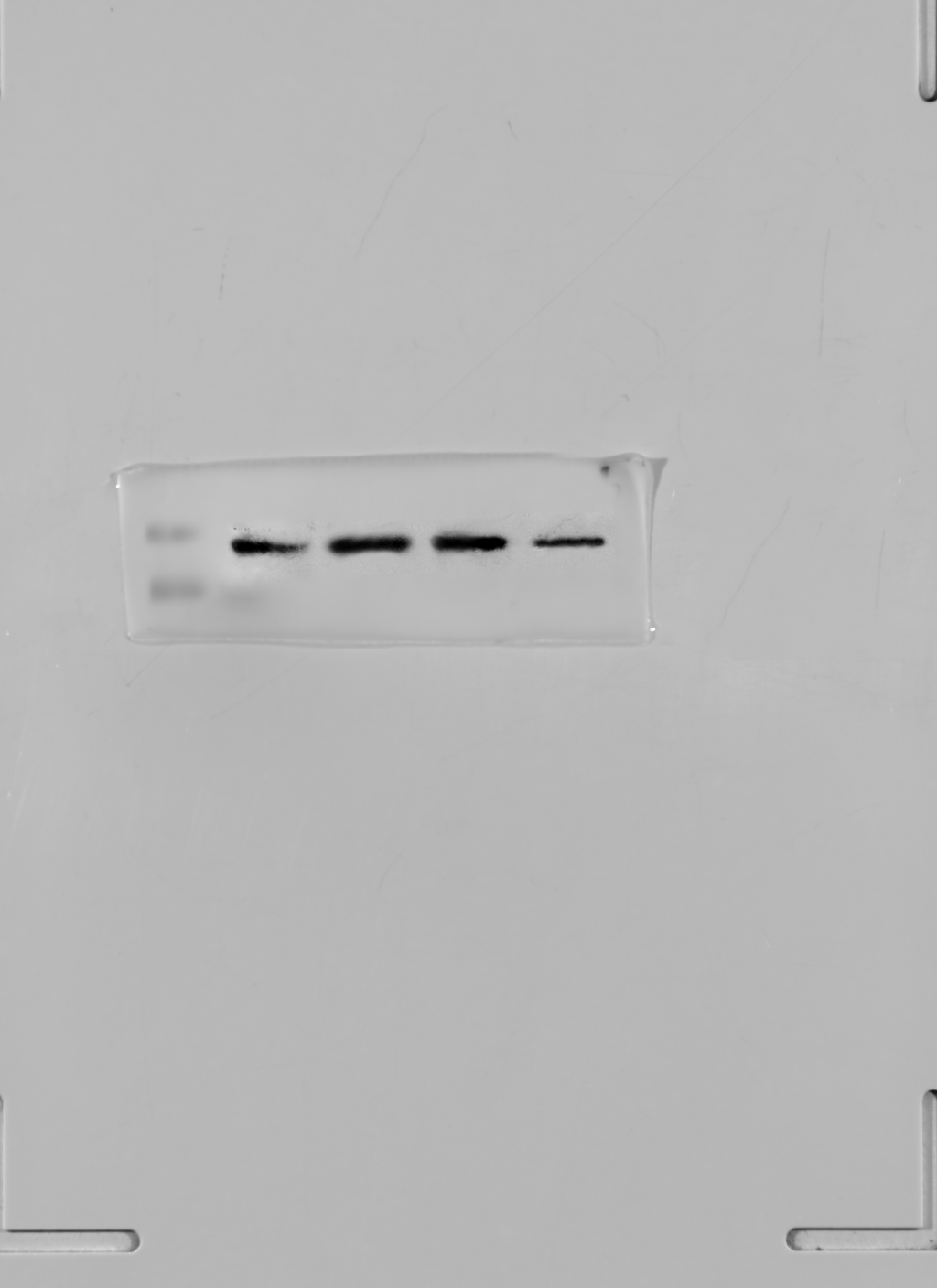

Supplement: Supplementary file 1 [file animals-15-00365-s001.zip › S1/WB original-241221/Fig4/Fig.4 ATG5-原图1.png]

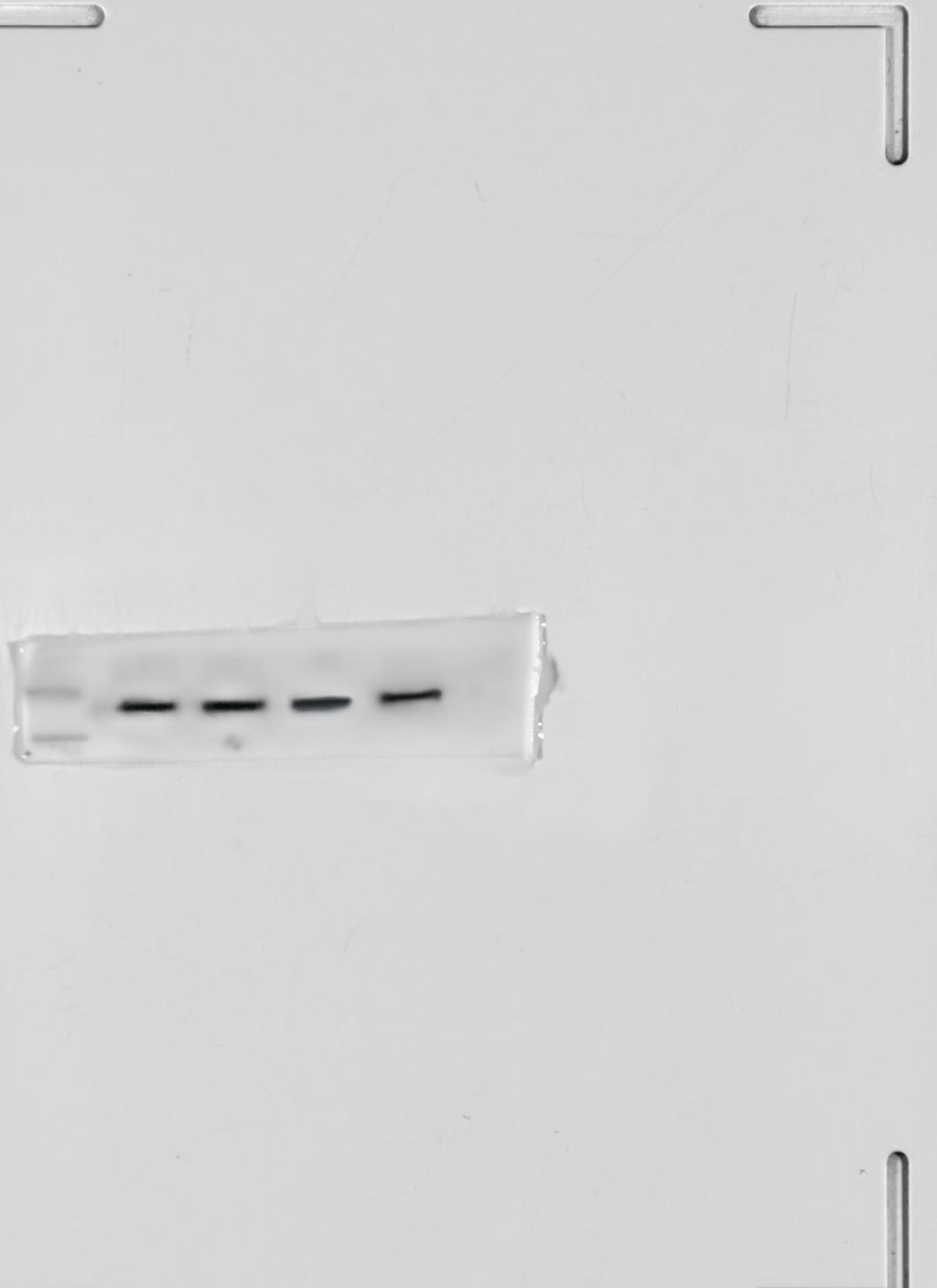

Supplement: Supplementary file 1 [file animals-15-00365-s001.zip › S1/WB original-241221/Fig4/Fig.4 ATG5-原图2.png]

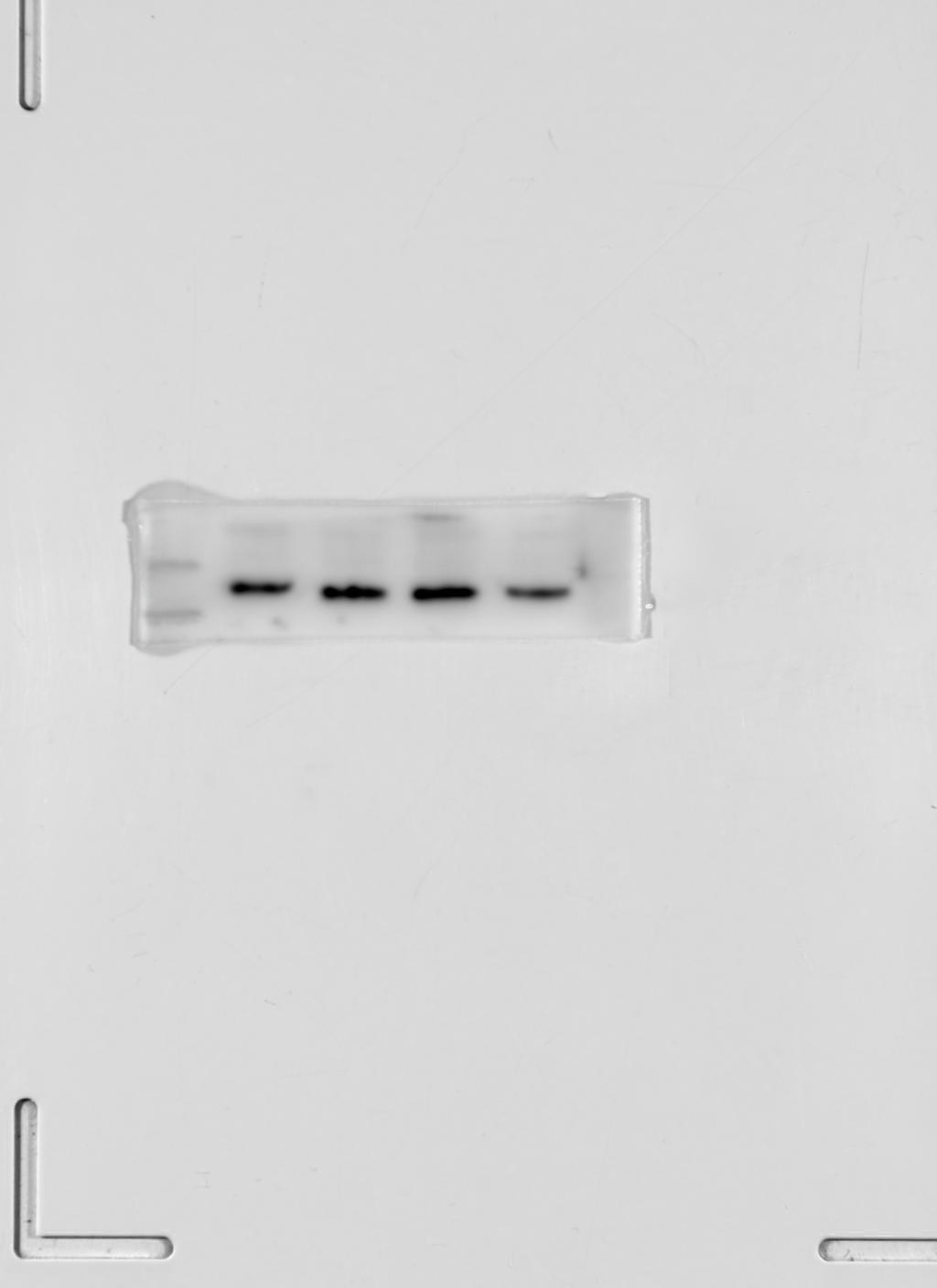

Supplement: Supplementary file 1 [file animals-15-00365-s001.zip › S1/WB original-241221/Fig4/Fig.4 ATG5-原图3.png]

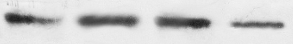

Supplement: Supplementary file 1 [file animals-15-00365-s001.zip › S1/WB original-241221/Fig4/Fig.4 ATG5.png]

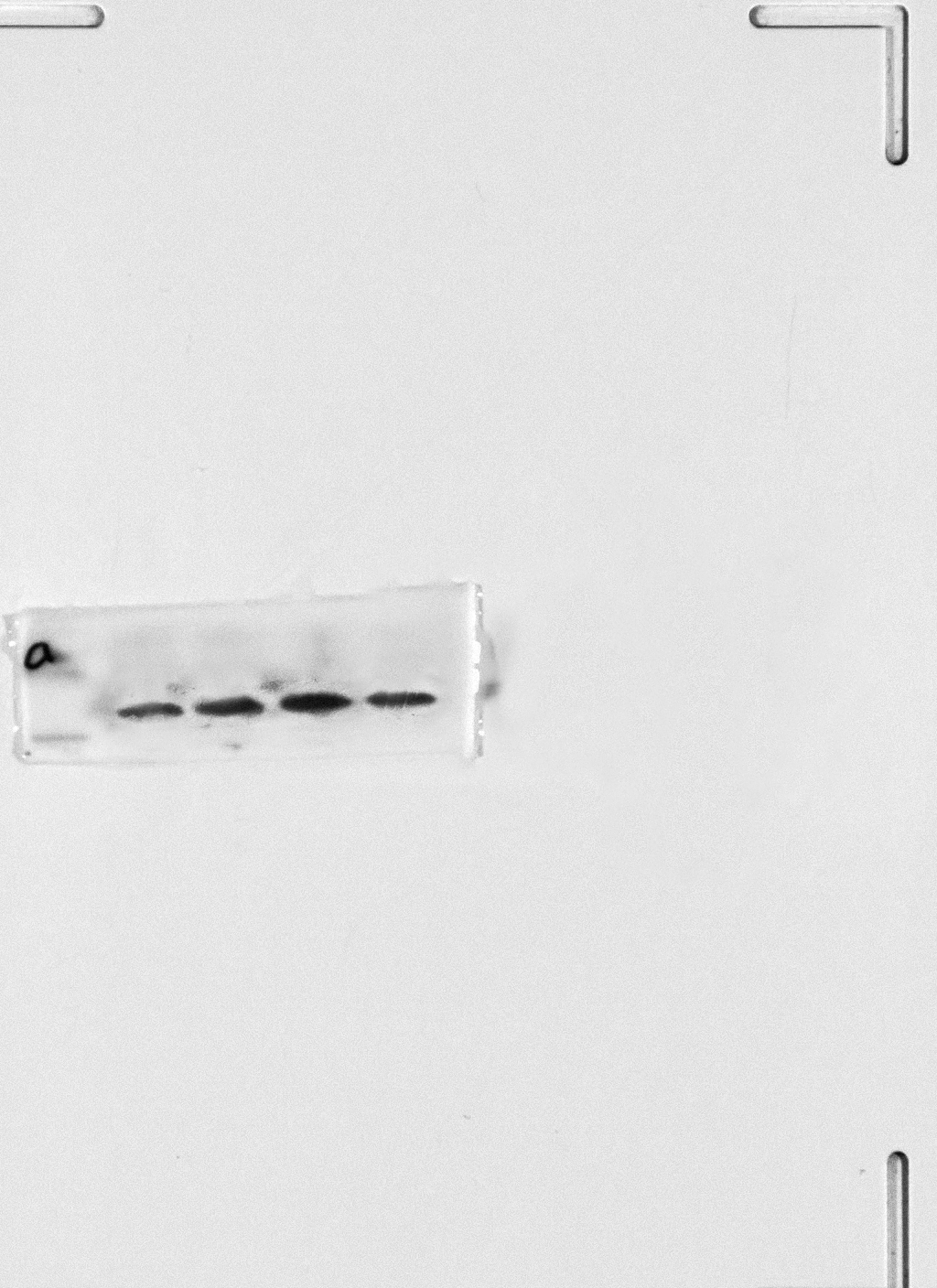

Supplement: Supplementary file 1 [file animals-15-00365-s001.zip › S1/WB original-241221/Fig4/Fig.4 BECN1-原图1.png]

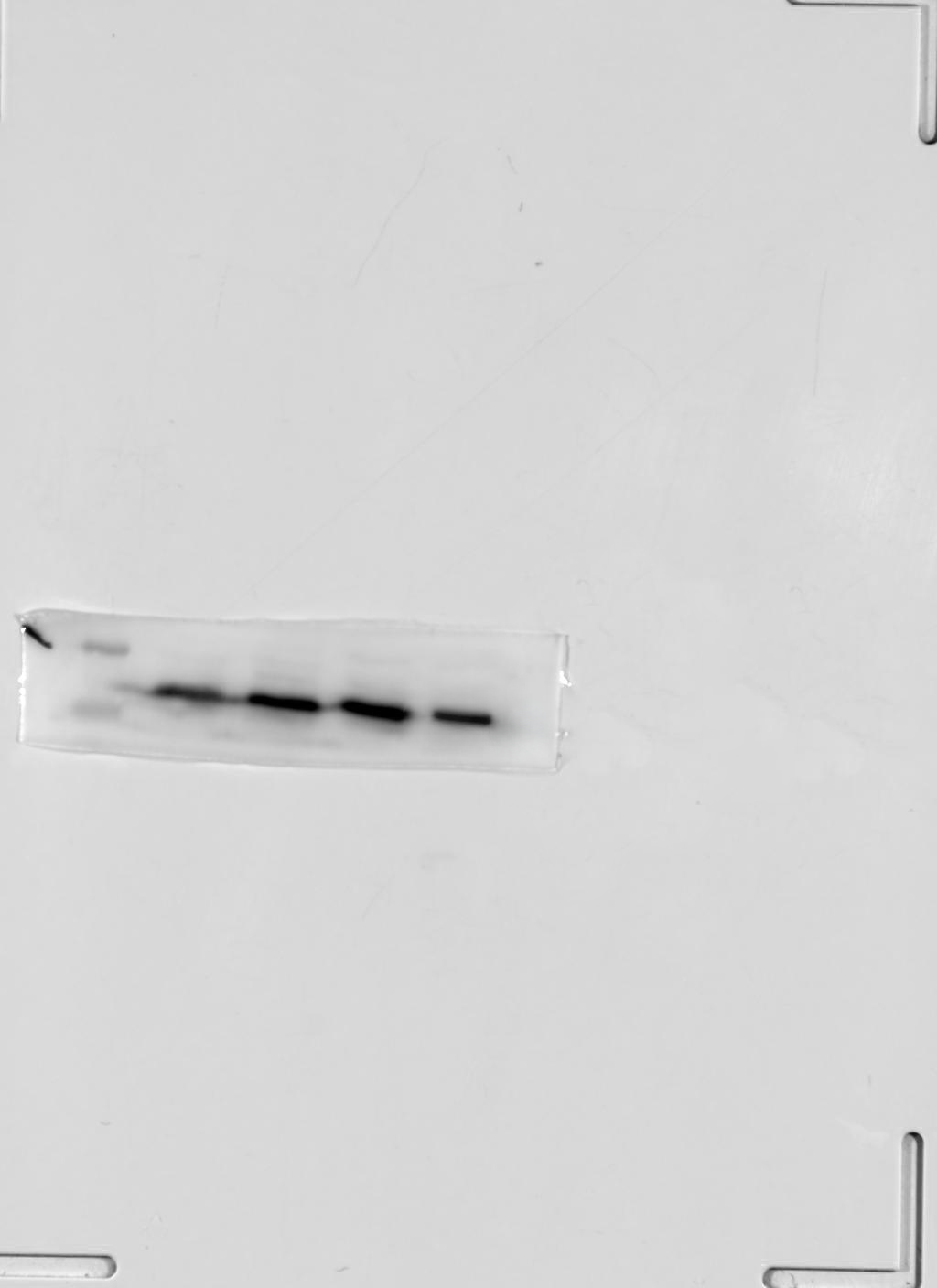

Supplement: Supplementary file 1 [file animals-15-00365-s001.zip › S1/WB original-241221/Fig4/Fig.4 BECN1-原图2.png]

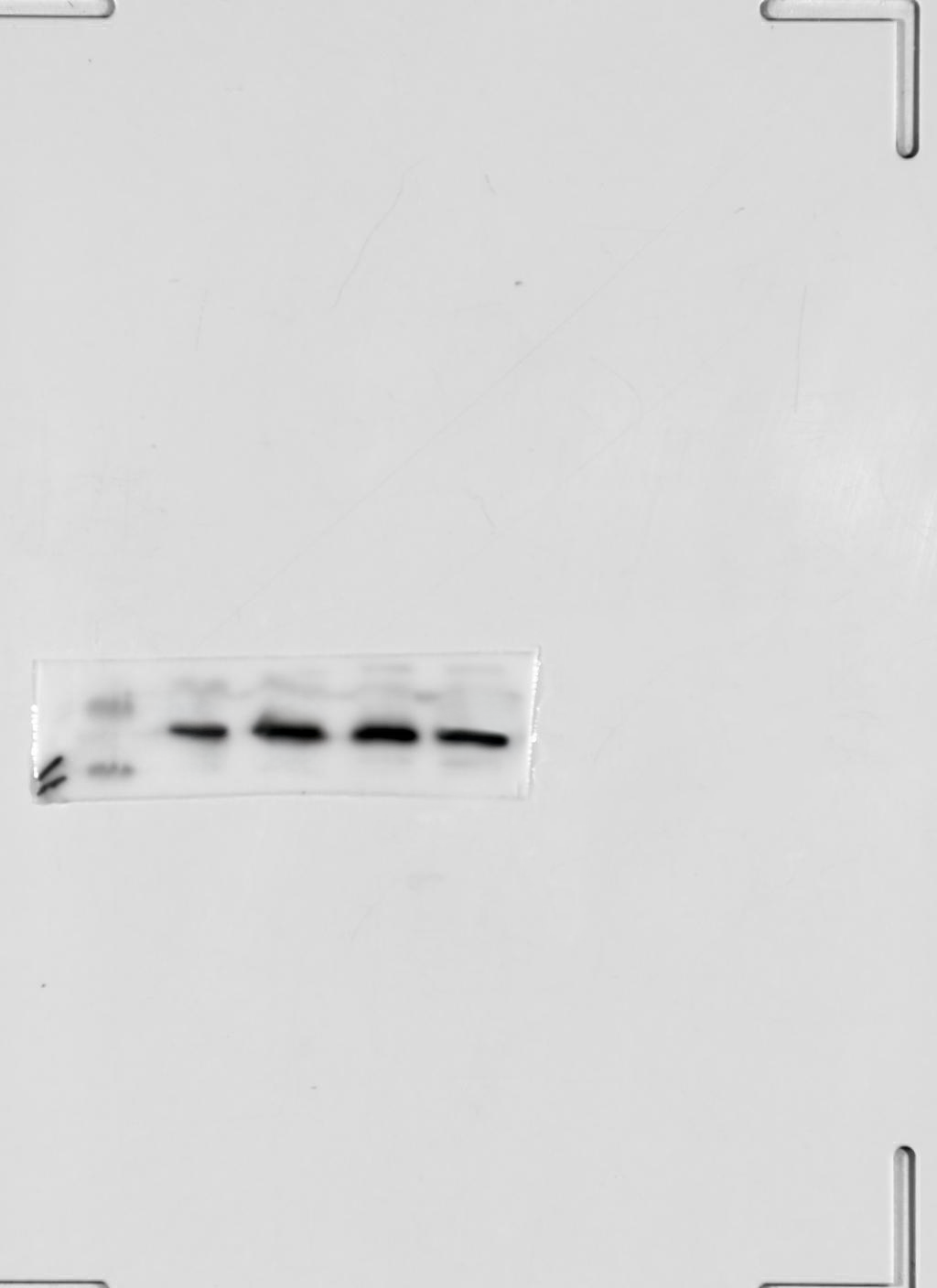

Supplement: Supplementary file 1 [file animals-15-00365-s001.zip › S1/WB original-241221/Fig4/Fig.4 BECN1-原图3.png]

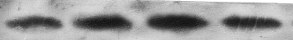

Supplement: Supplementary file 1 [file animals-15-00365-s001.zip › S1/WB original-241221/Fig4/Fig.4 BECN1.png]

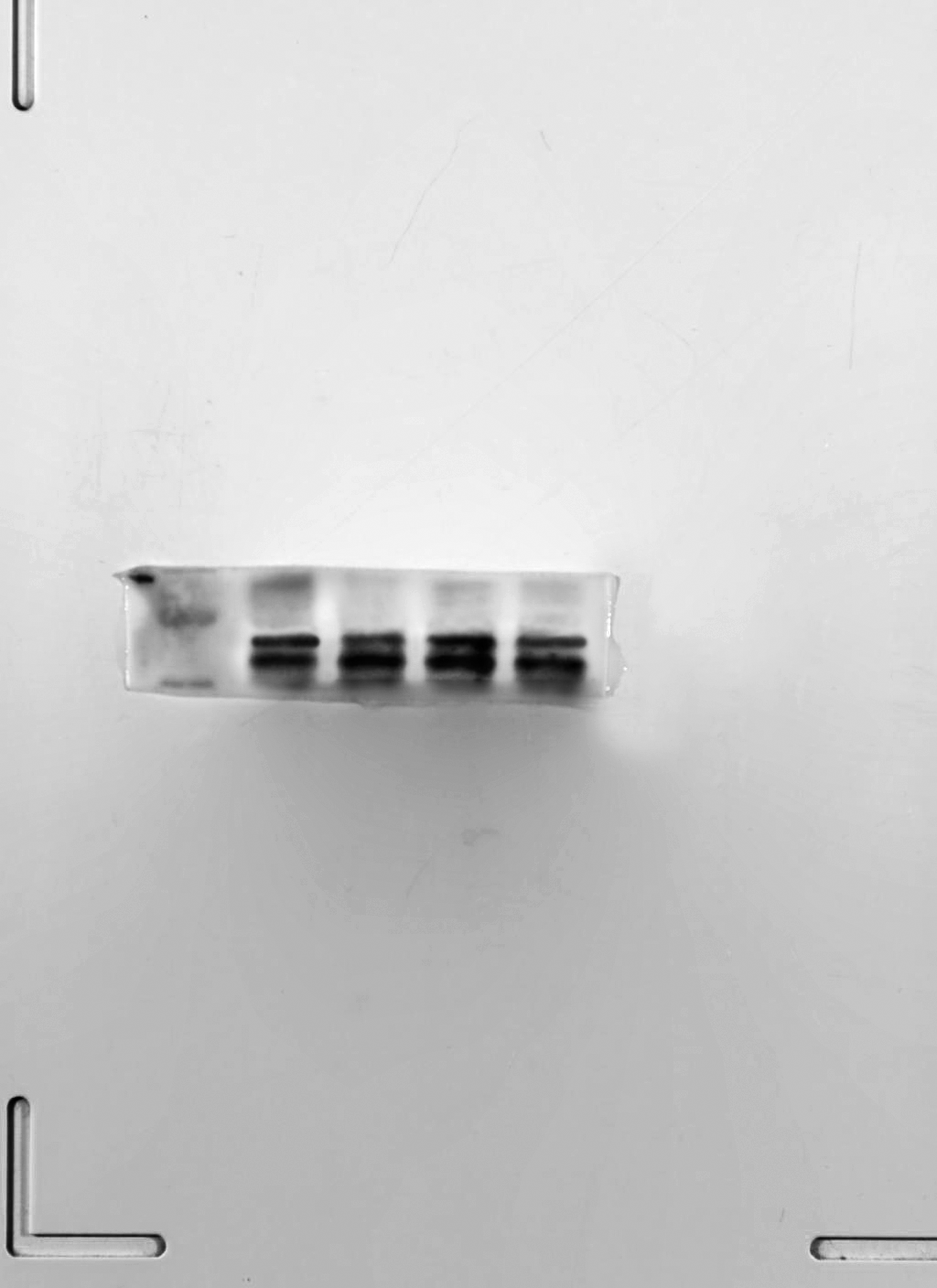

Supplement: Supplementary file 1 [file animals-15-00365-s001.zip › S1/WB original-241221/Fig4/Fig.4 LC3-I_II-原图1.png]

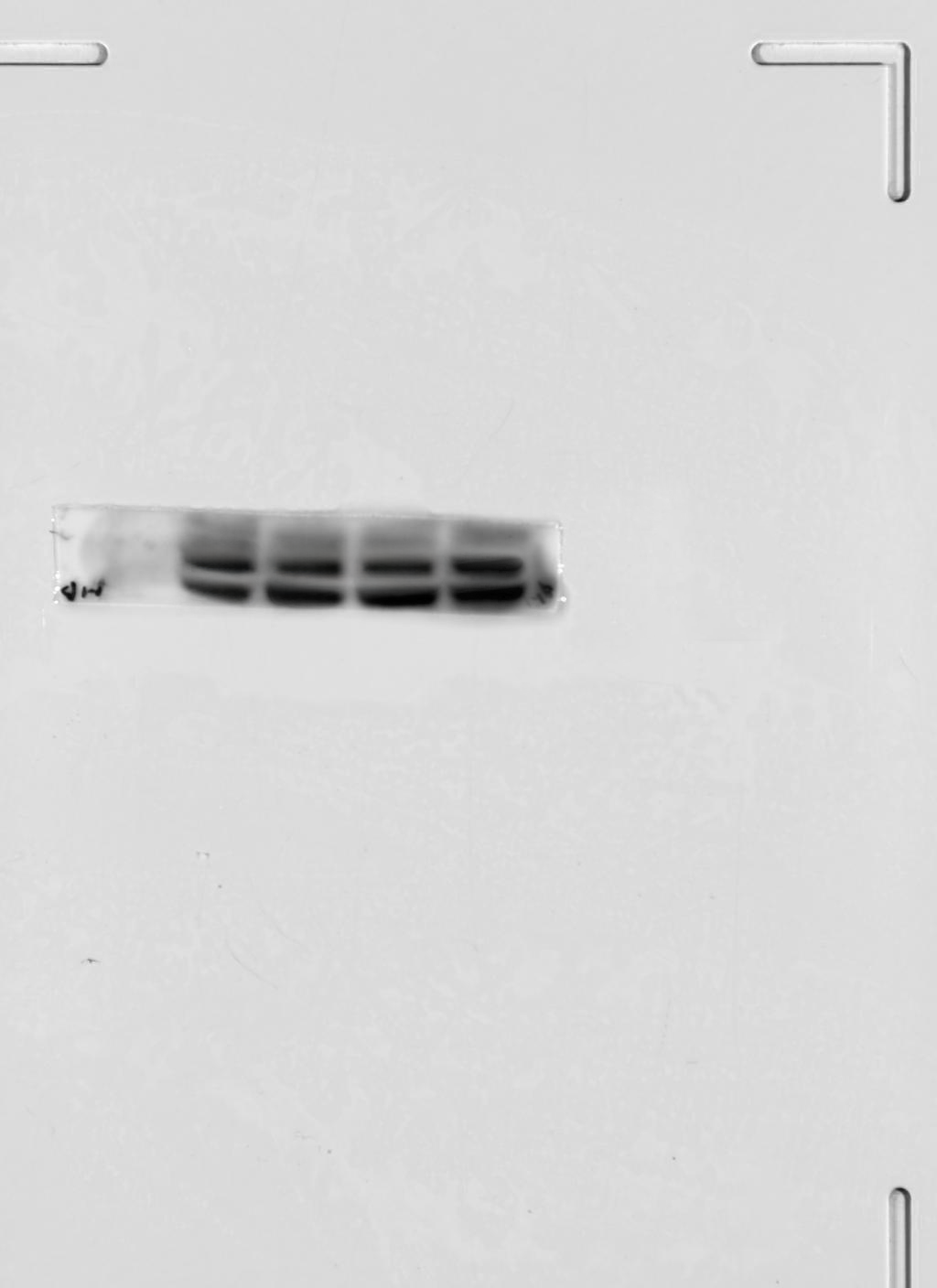

Supplement: Supplementary file 1 [file animals-15-00365-s001.zip › S1/WB original-241221/Fig4/Fig.4 LC3-I_II-原图2.png]

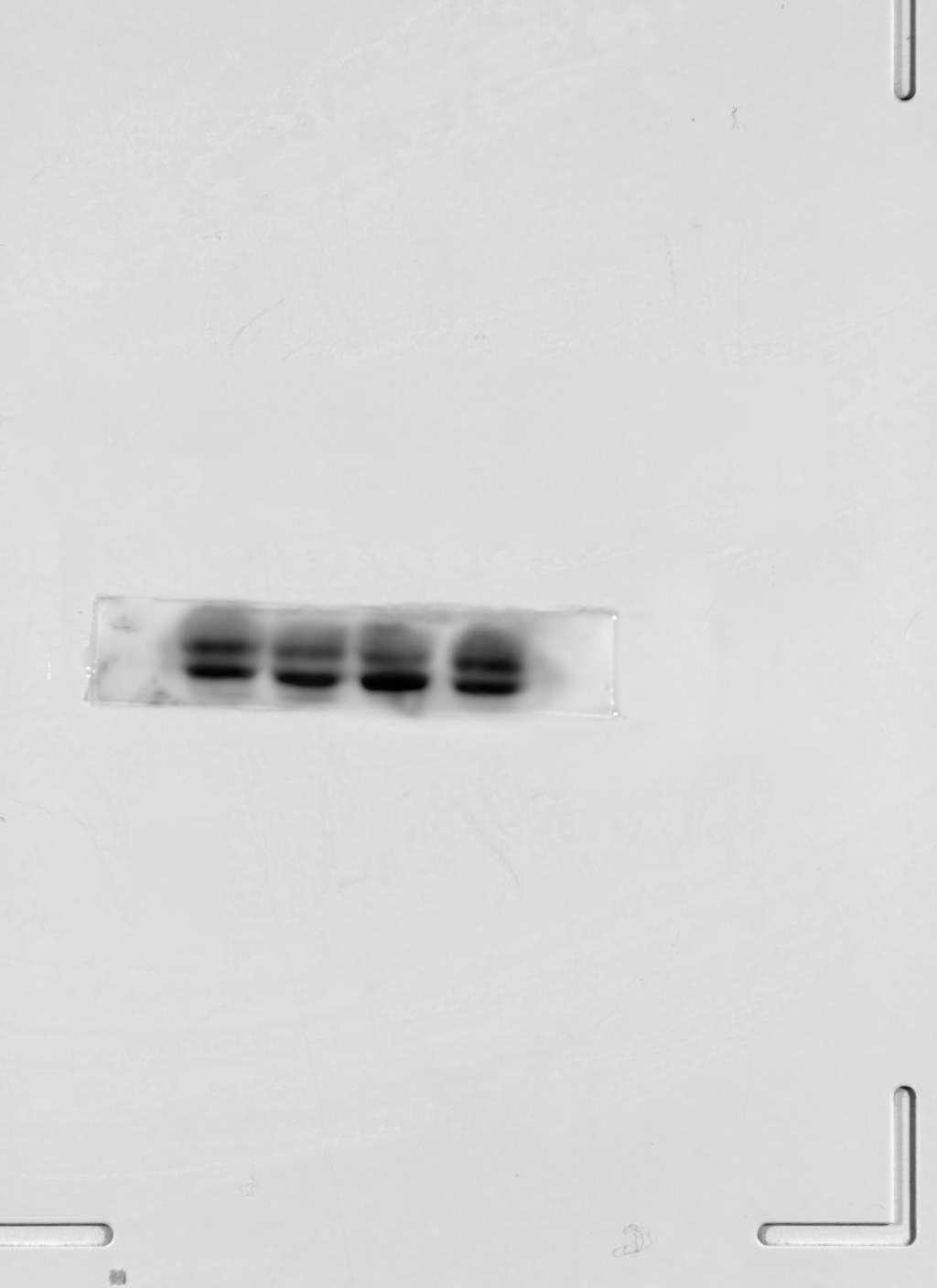

Supplement: Supplementary file 1 [file animals-15-00365-s001.zip › S1/WB original-241221/Fig4/Fig.4 LC3-I_II-原图3.png]

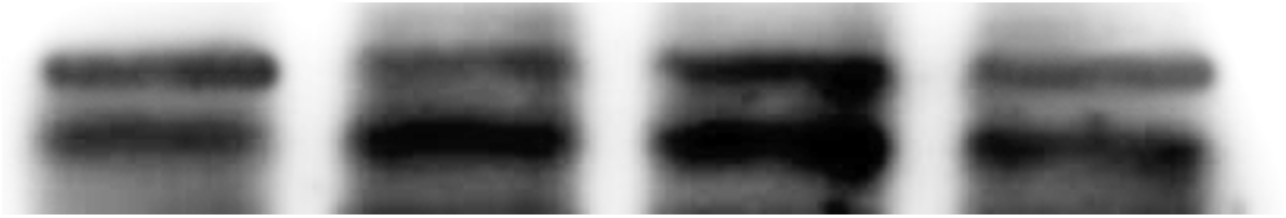

Supplement: Supplementary file 1 [file animals-15-00365-s001.zip › S1/WB original-241221/Fig4/Fig.4 LC3-I_II.png]

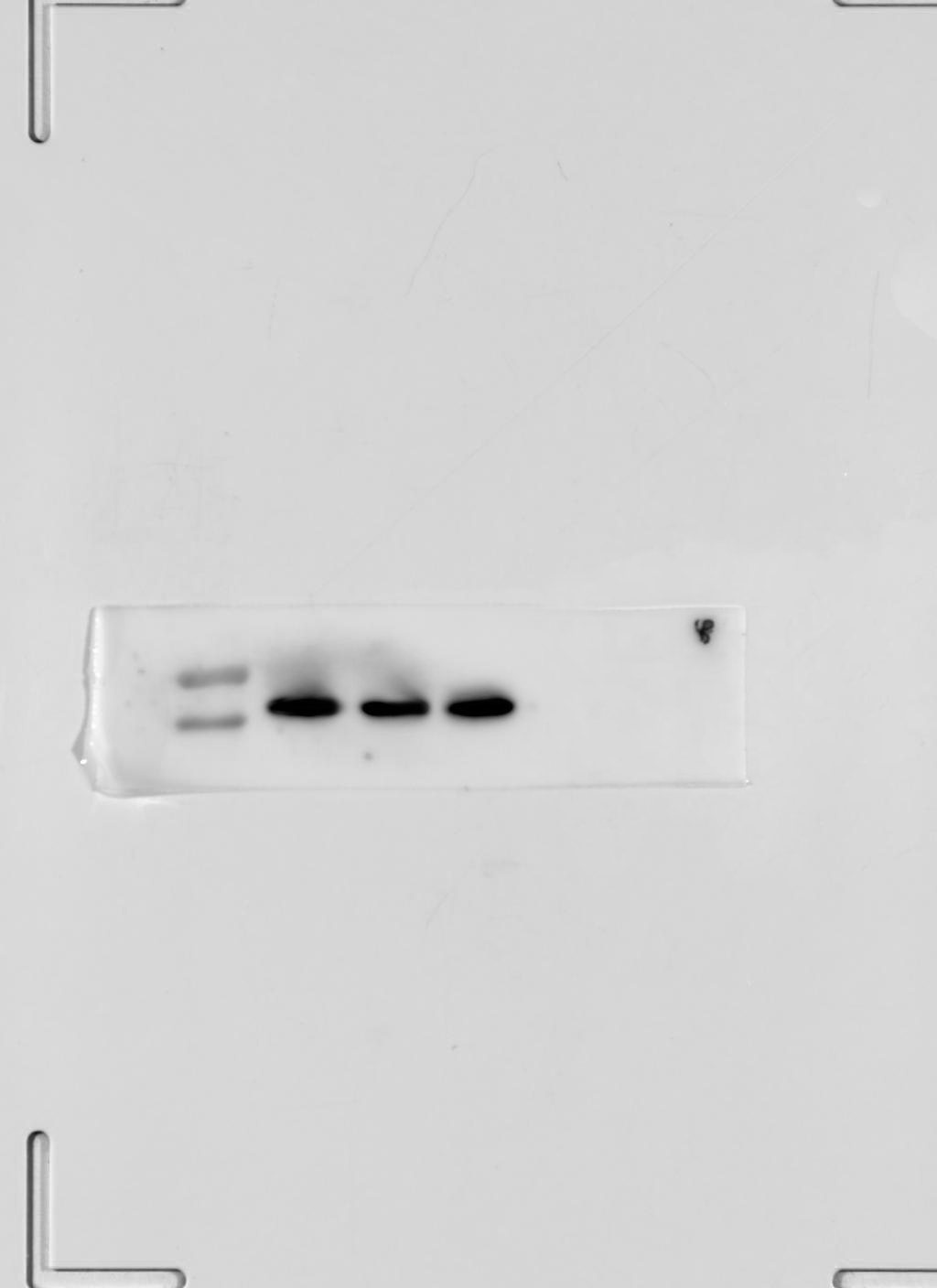

Supplement: Supplementary file 1 [file animals-15-00365-s001.zip › S1/WB original-241221/Fig6/Fig.6 actin-原图1.png]

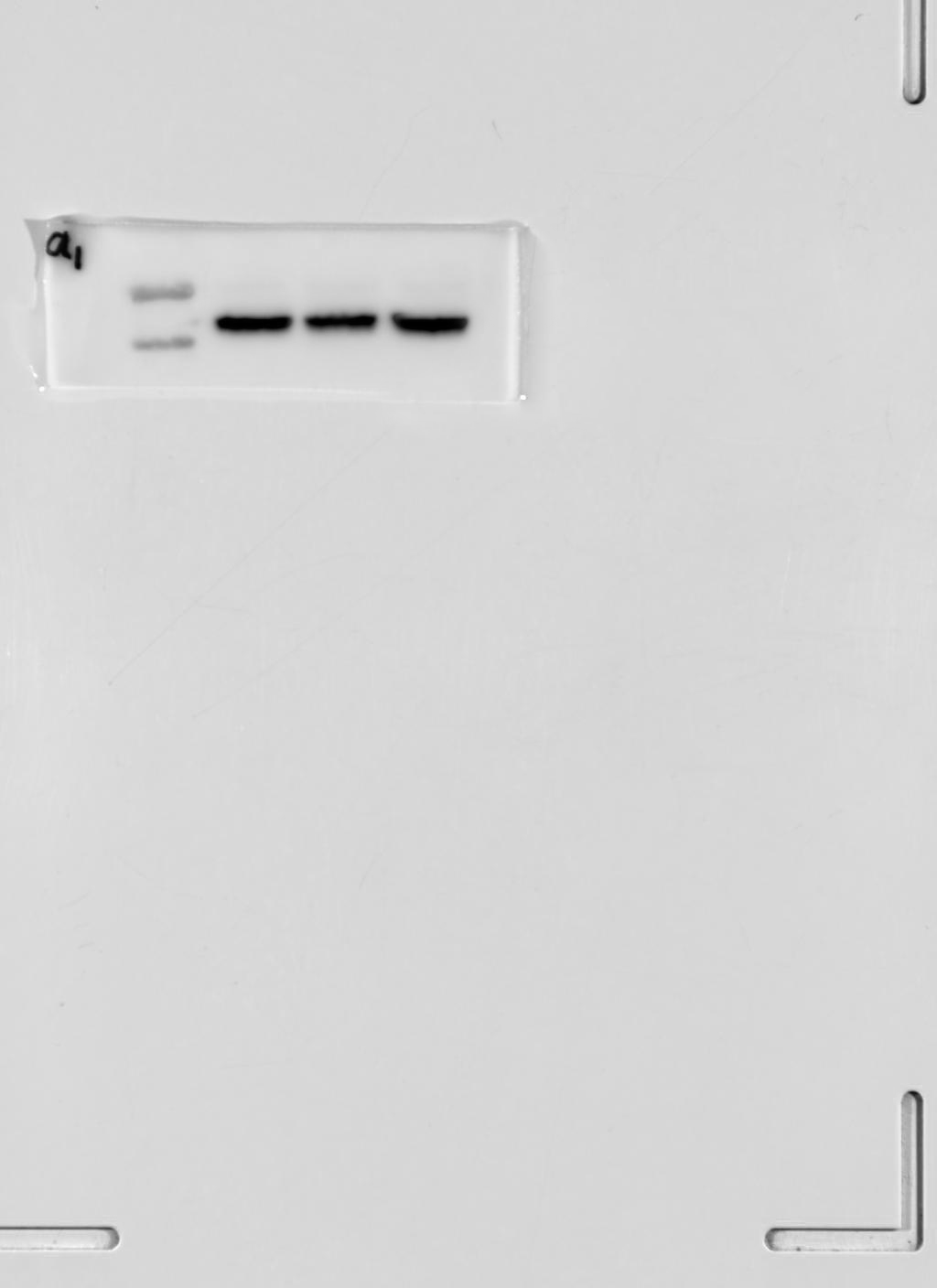

Supplement: Supplementary file 1 [file animals-15-00365-s001.zip › S1/WB original-241221/Fig6/Fig.6 actin-原图2.png]

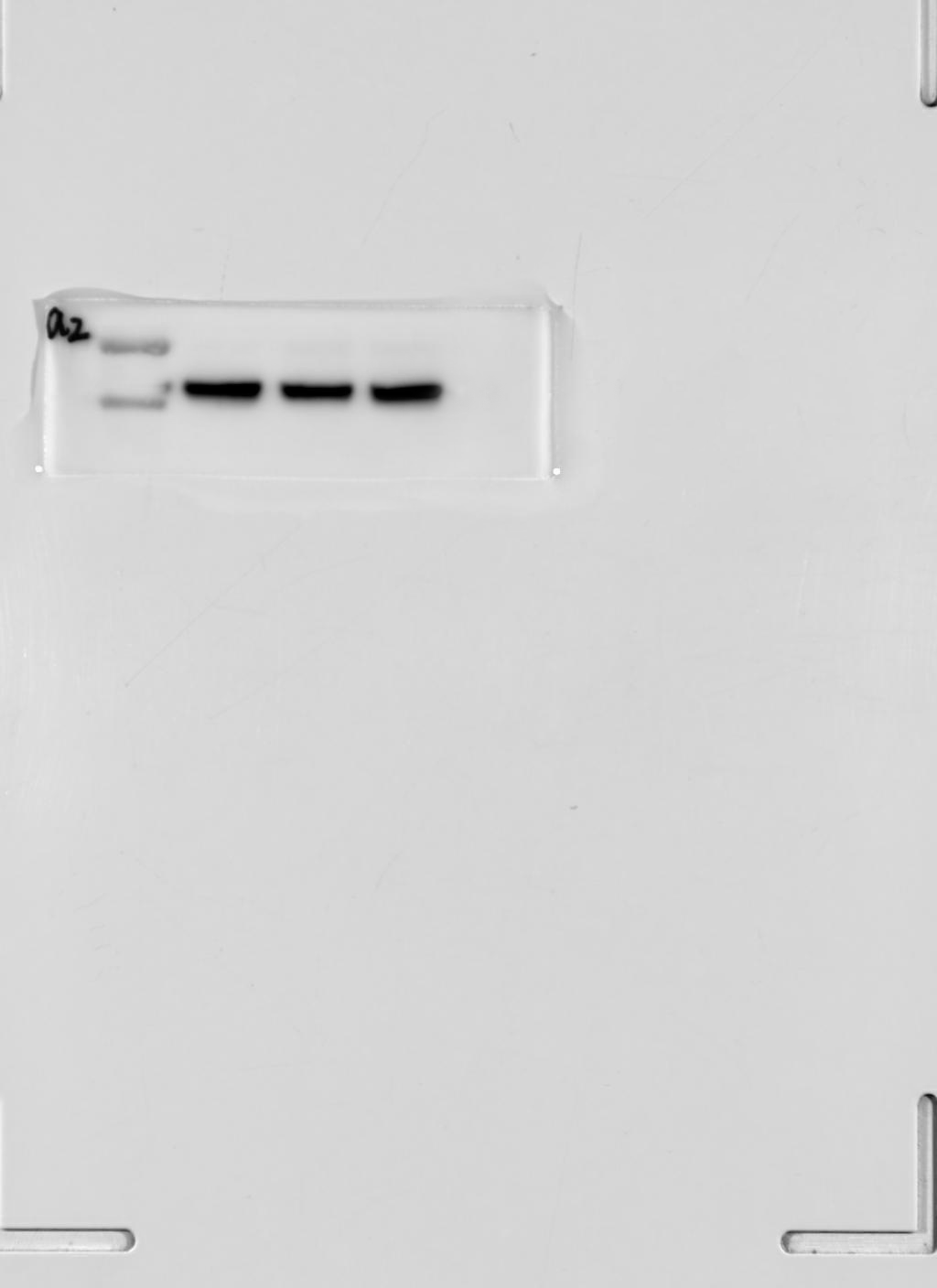

Supplement: Supplementary file 1 [file animals-15-00365-s001.zip › S1/WB original-241221/Fig6/Fig.6 actin-原图3.png]

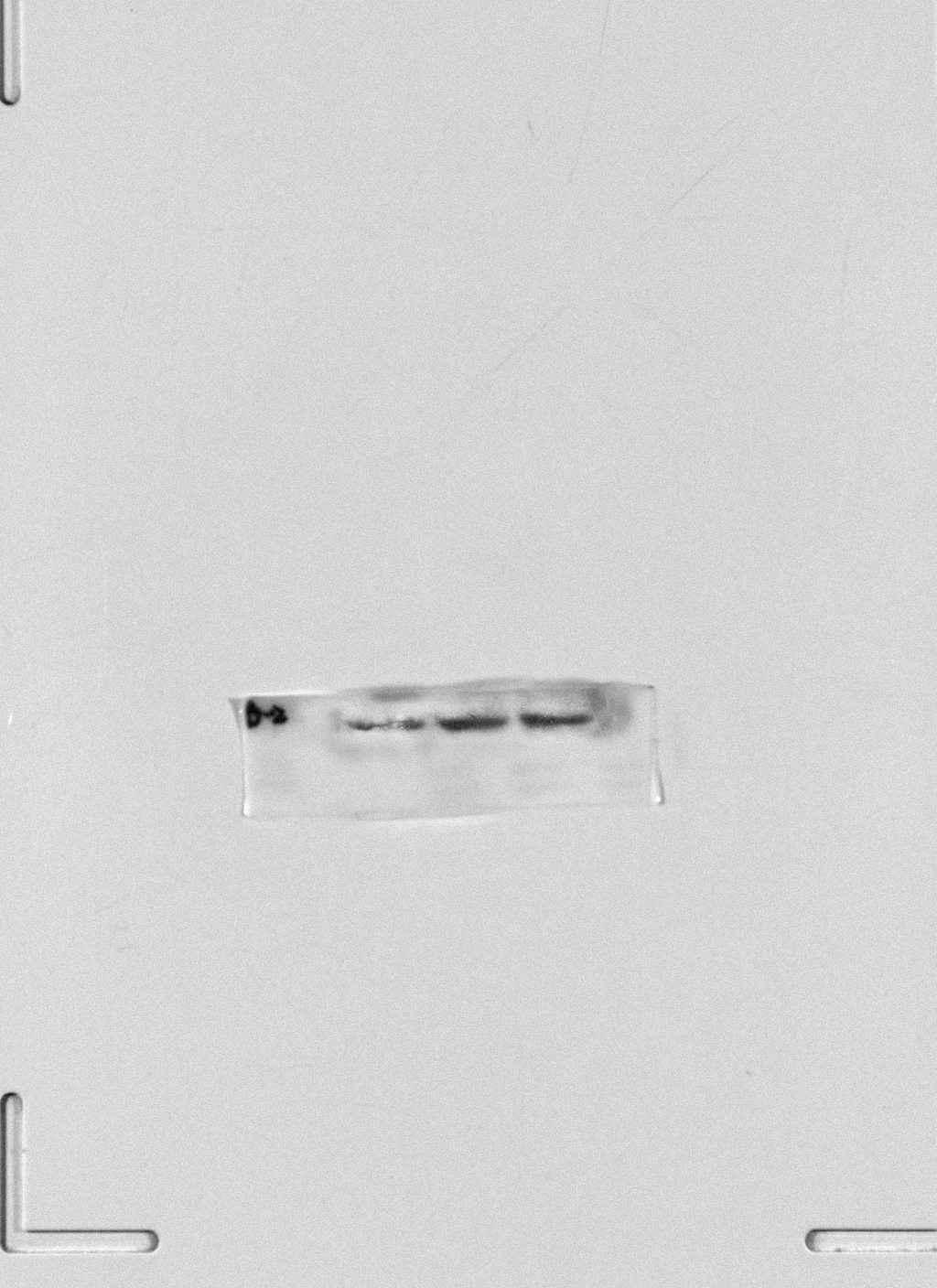

Supplement: Supplementary file 1 [file animals-15-00365-s001.zip › S1/WB original-241221/Fig6/Fig.6 HIF-1a-原图.png]

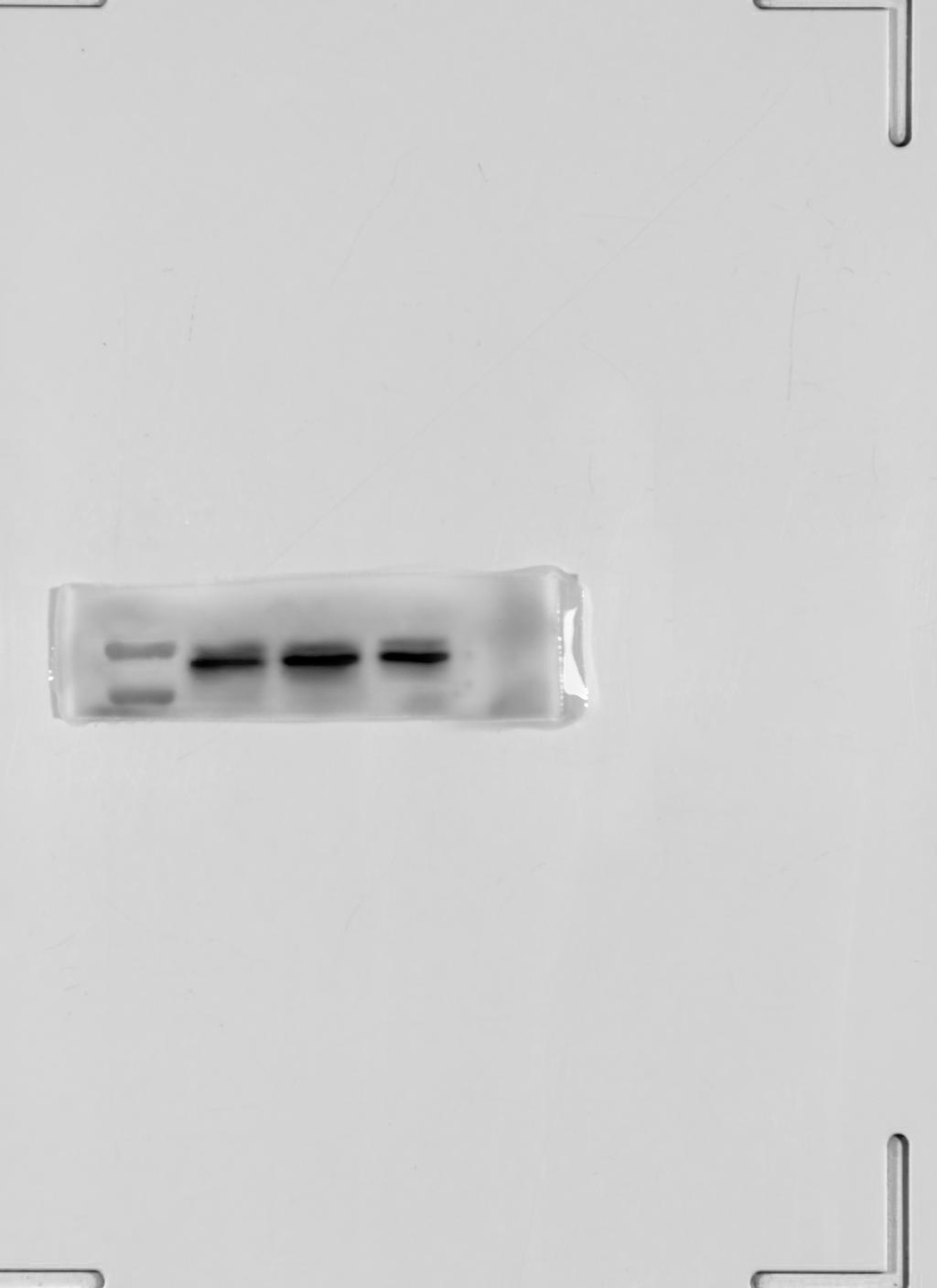

Supplement: Supplementary file 1 [file animals-15-00365-s001.zip › S1/WB original-241221/Fig6/Fig.6 HIF-1a-原图2.png]

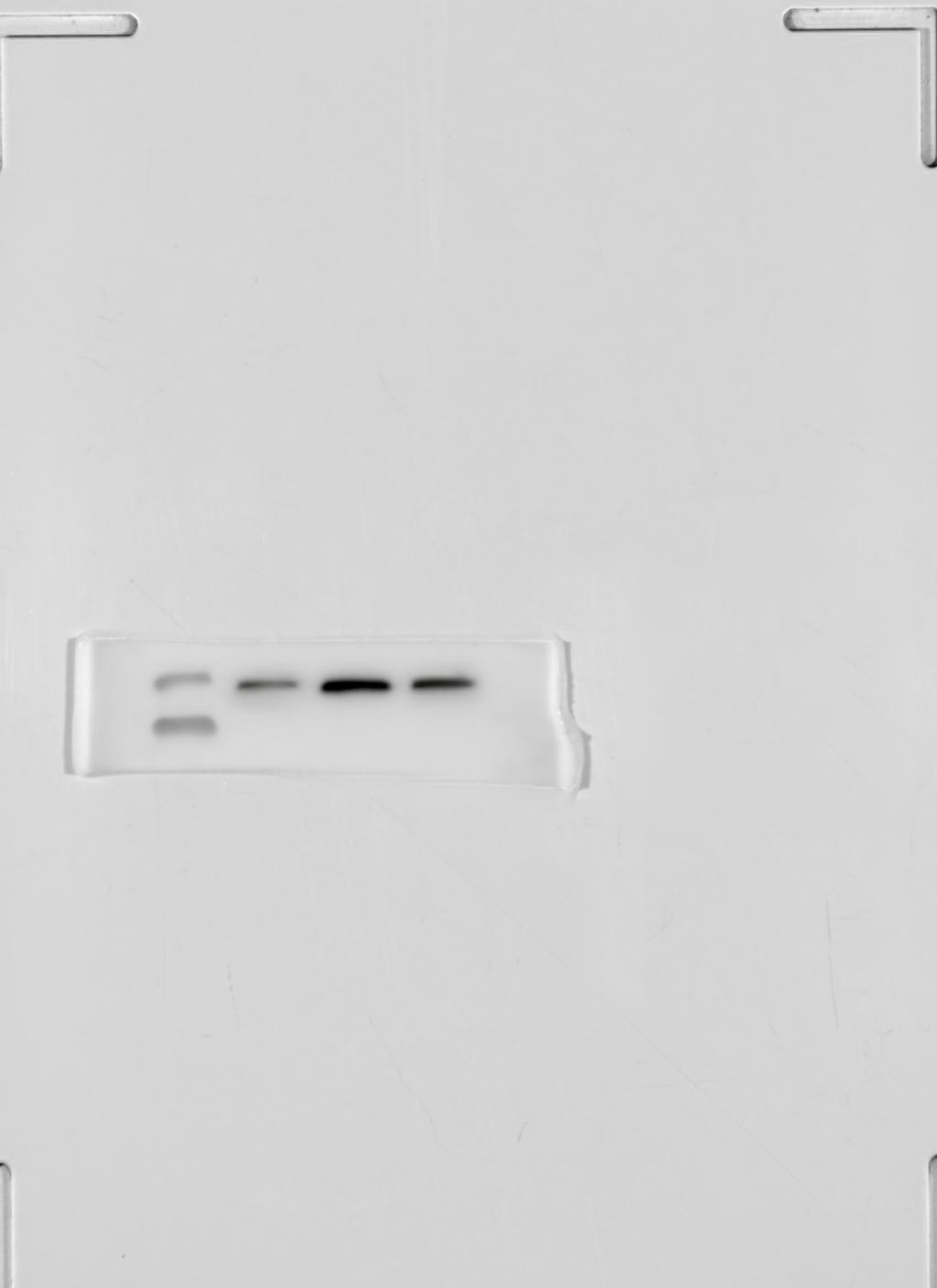

Supplement: Supplementary file 1 [file animals-15-00365-s001.zip › S1/WB original-241221/Fig6/Fig.6 HIF-1a-原图3.png]

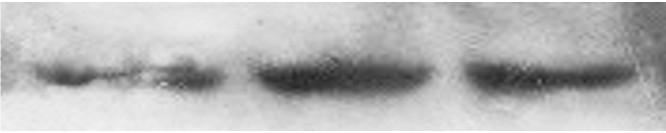

Supplement: Supplementary file 1 [file animals-15-00365-s001.zip › S1/WB original-241221/Fig6/Fig.6 HIF-1a.png]

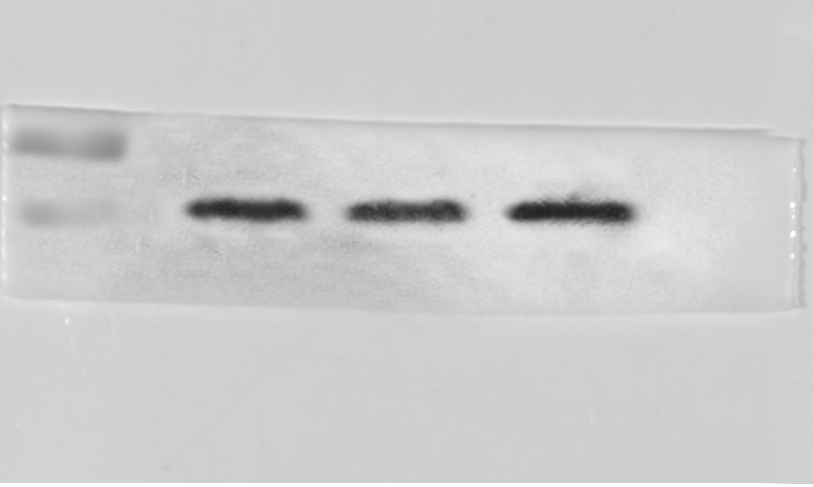

Supplement: Supplementary file 1 [file animals-15-00365-s001.zip › S1/WB original-241221/Fig7/Fig.7 actin-原图1.png]

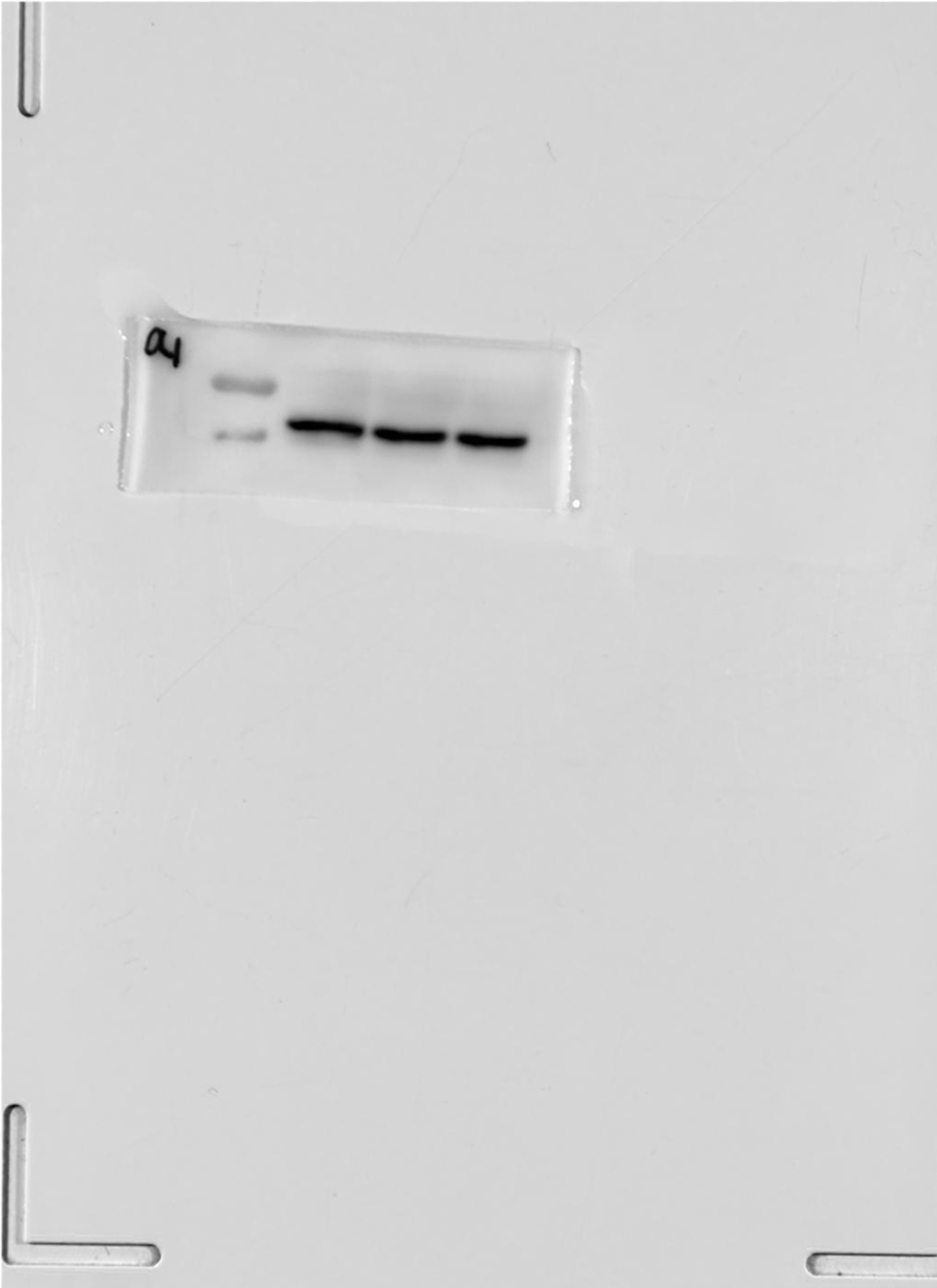

Supplement: Supplementary file 1 [file animals-15-00365-s001.zip › S1/WB original-241221/Fig7/Fig.7 actin-原图2.png]

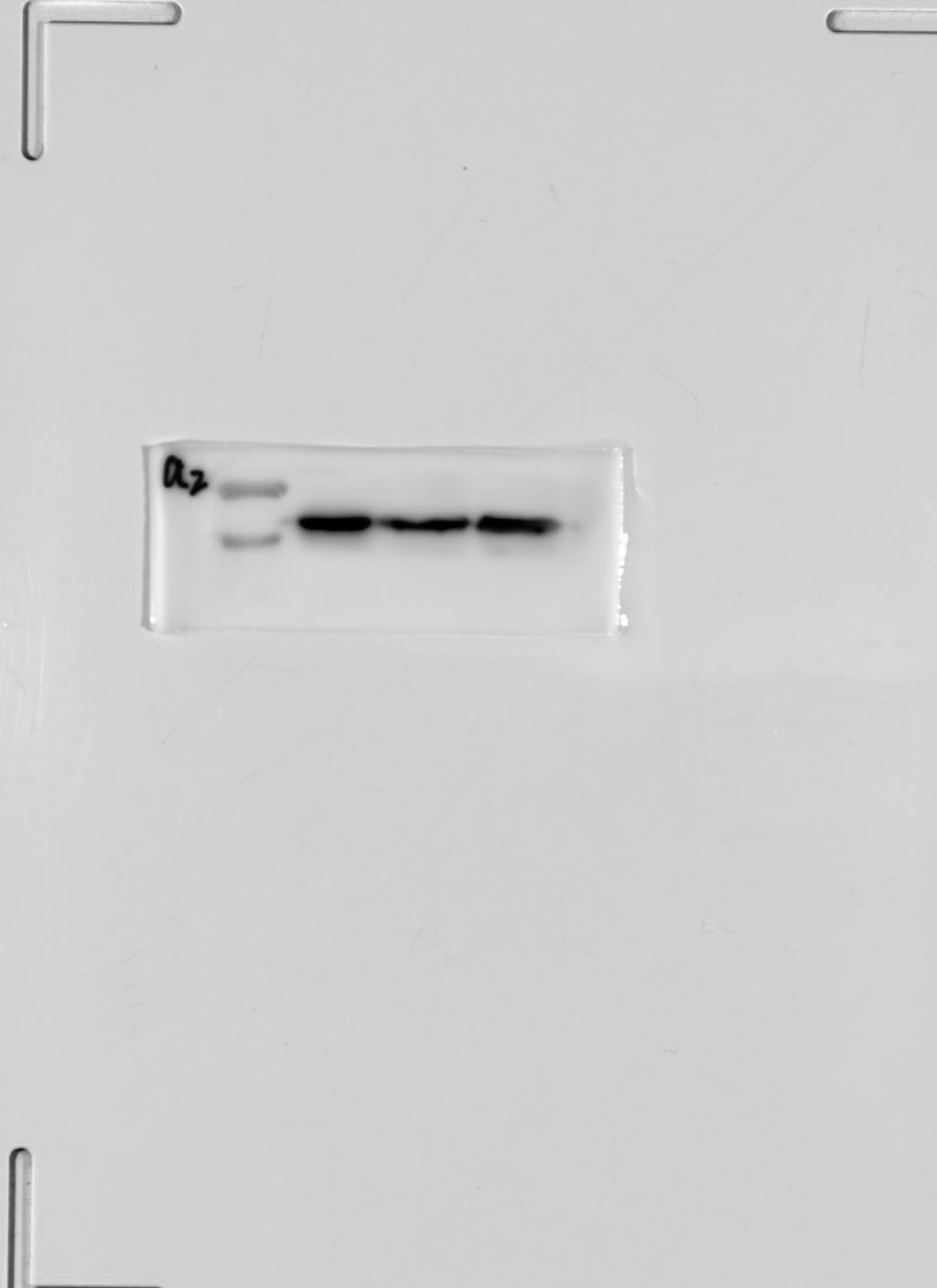

Supplement: Supplementary file 1 [file animals-15-00365-s001.zip › S1/WB original-241221/Fig7/Fig.7 actin-原图3.png]

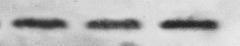

Supplement: Supplementary file 1 [file animals-15-00365-s001.zip › S1/WB original-241221/Fig7/Fig.7 actin.png]

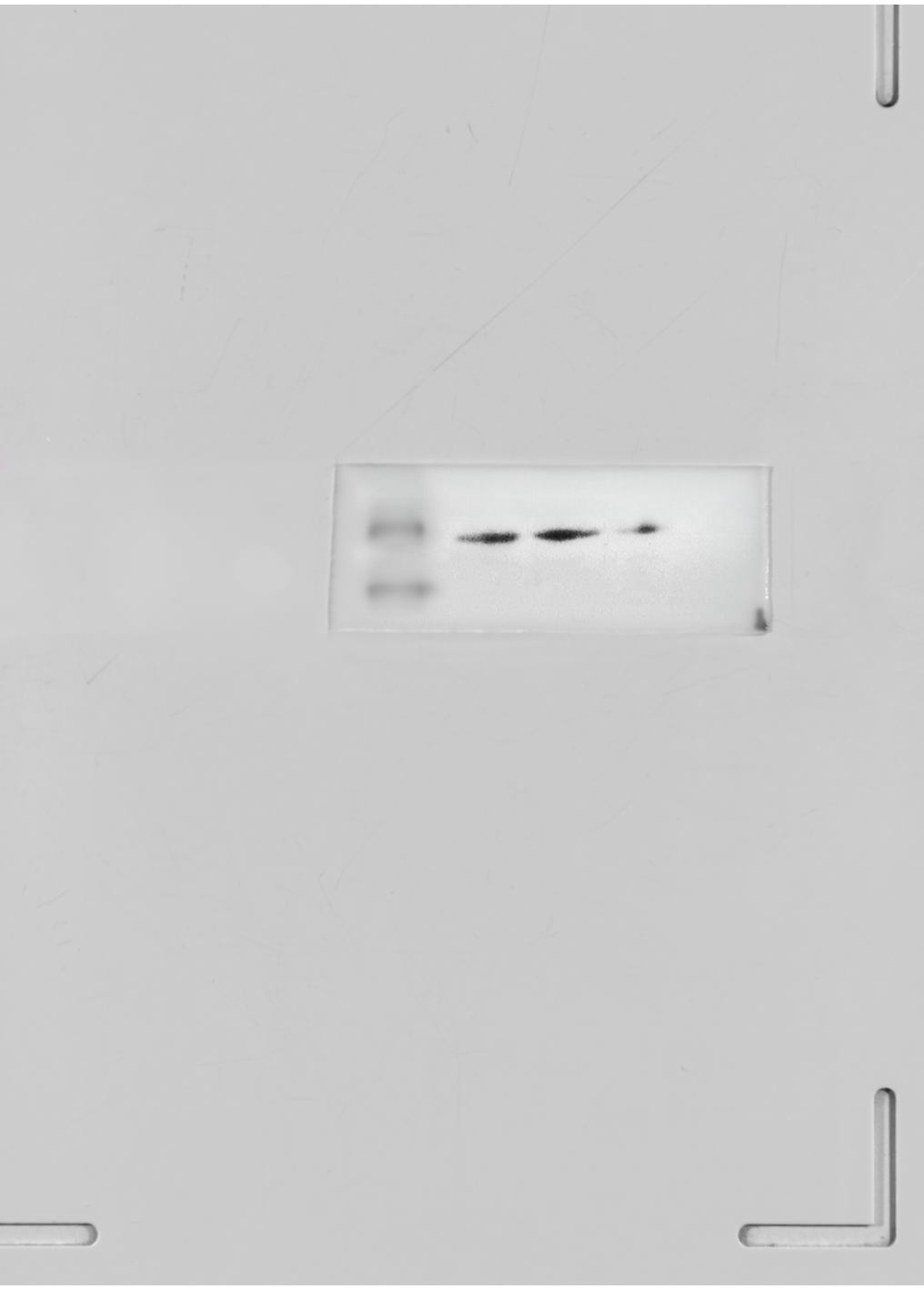

Supplement: Supplementary file 1 [file animals-15-00365-s001.zip › S1/WB original-241221/Fig7/Fig.7 ATG5-原图1.png]

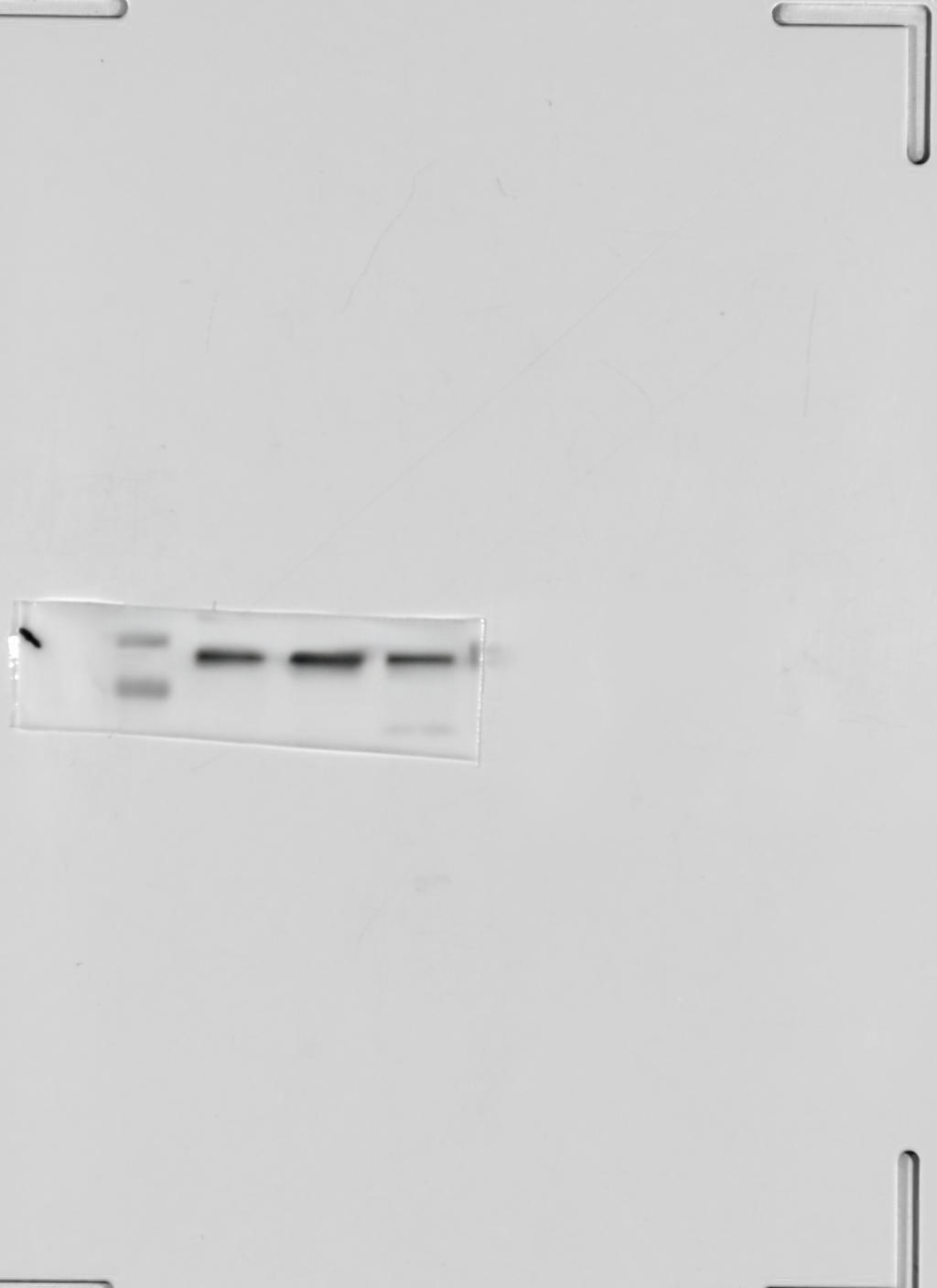

Supplement: Supplementary file 1 [file animals-15-00365-s001.zip › S1/WB original-241221/Fig7/Fig.7 ATG5-原图2.png]

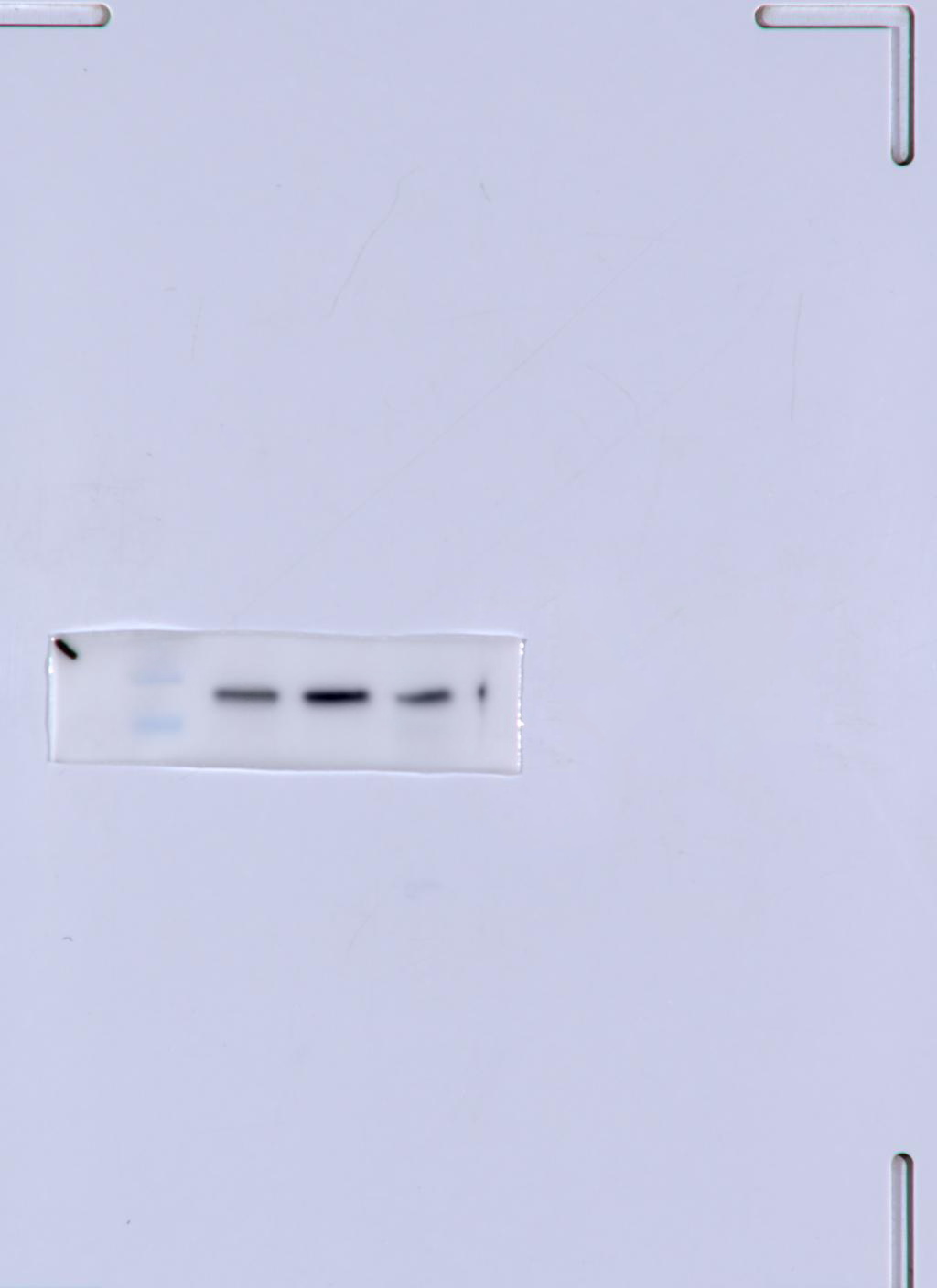

Supplement: Supplementary file 1 [file animals-15-00365-s001.zip › S1/WB original-241221/Fig7/Fig.7 ATG5-原图3.png]

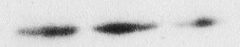

Supplement: Supplementary file 1 [file animals-15-00365-s001.zip › S1/WB original-241221/Fig7/Fig.7 ATG5.png]

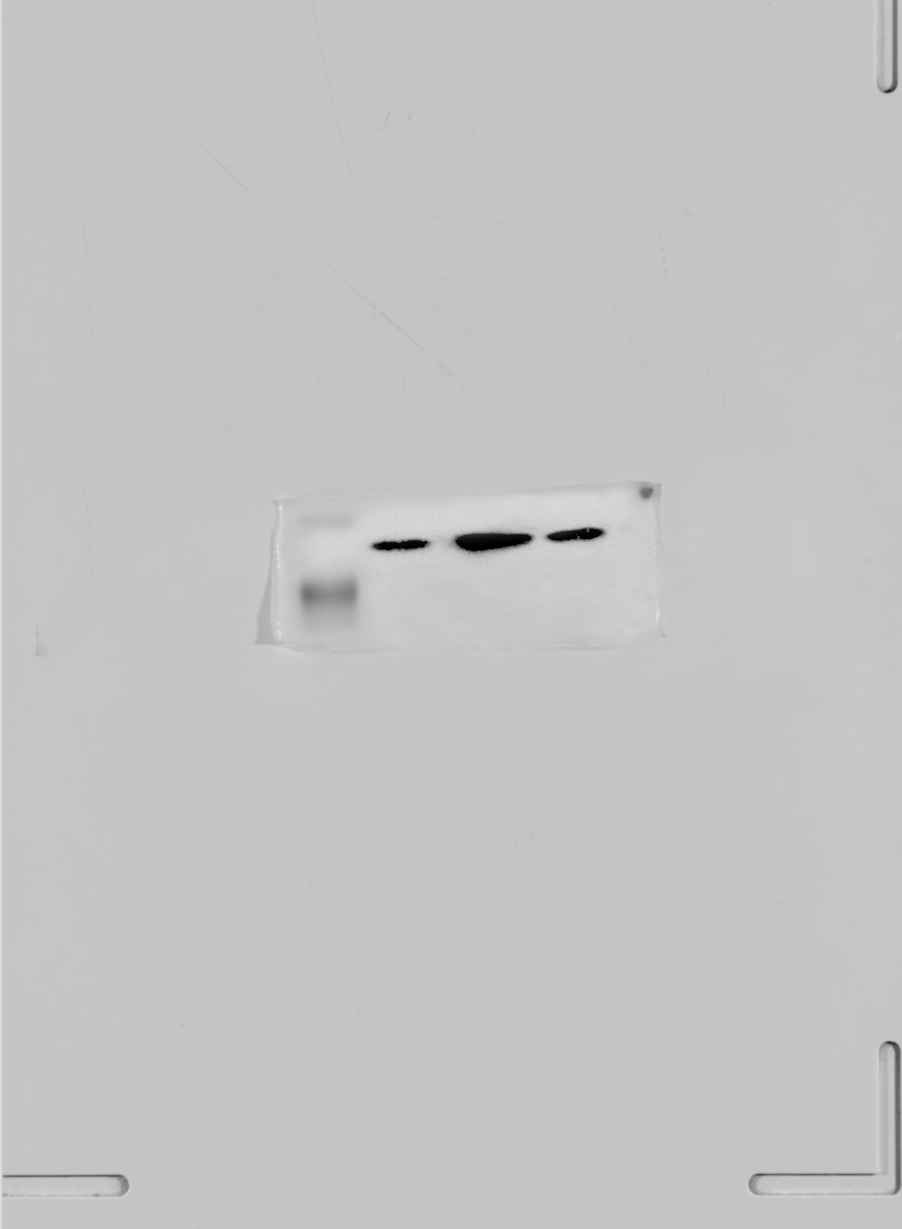

Supplement: Supplementary file 1 [file animals-15-00365-s001.zip › S1/WB original-241221/Fig7/Fig.7 BECN1-原图.png]

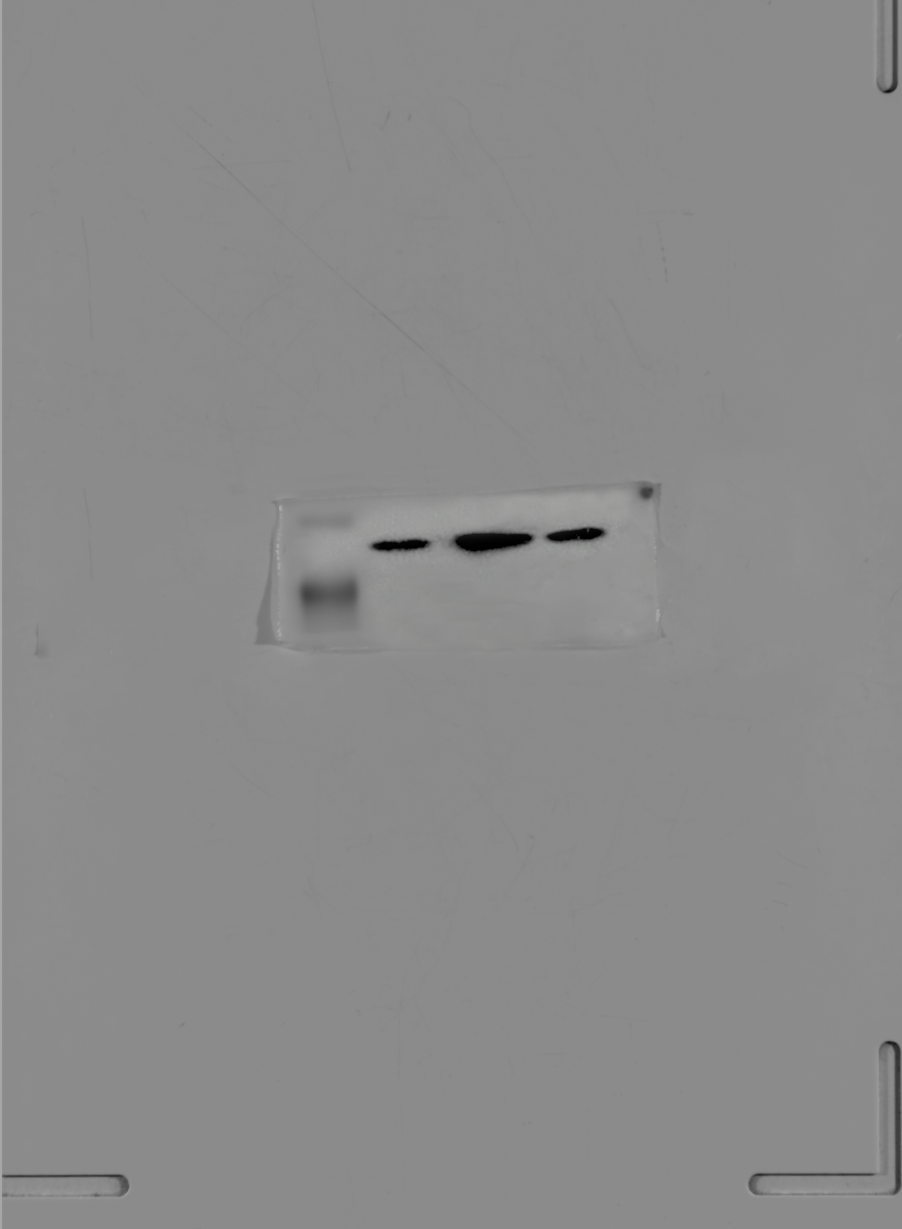

Supplement: Supplementary file 1 [file animals-15-00365-s001.zip › S1/WB original-241221/Fig7/Fig.7 BECN1-原图1.png]

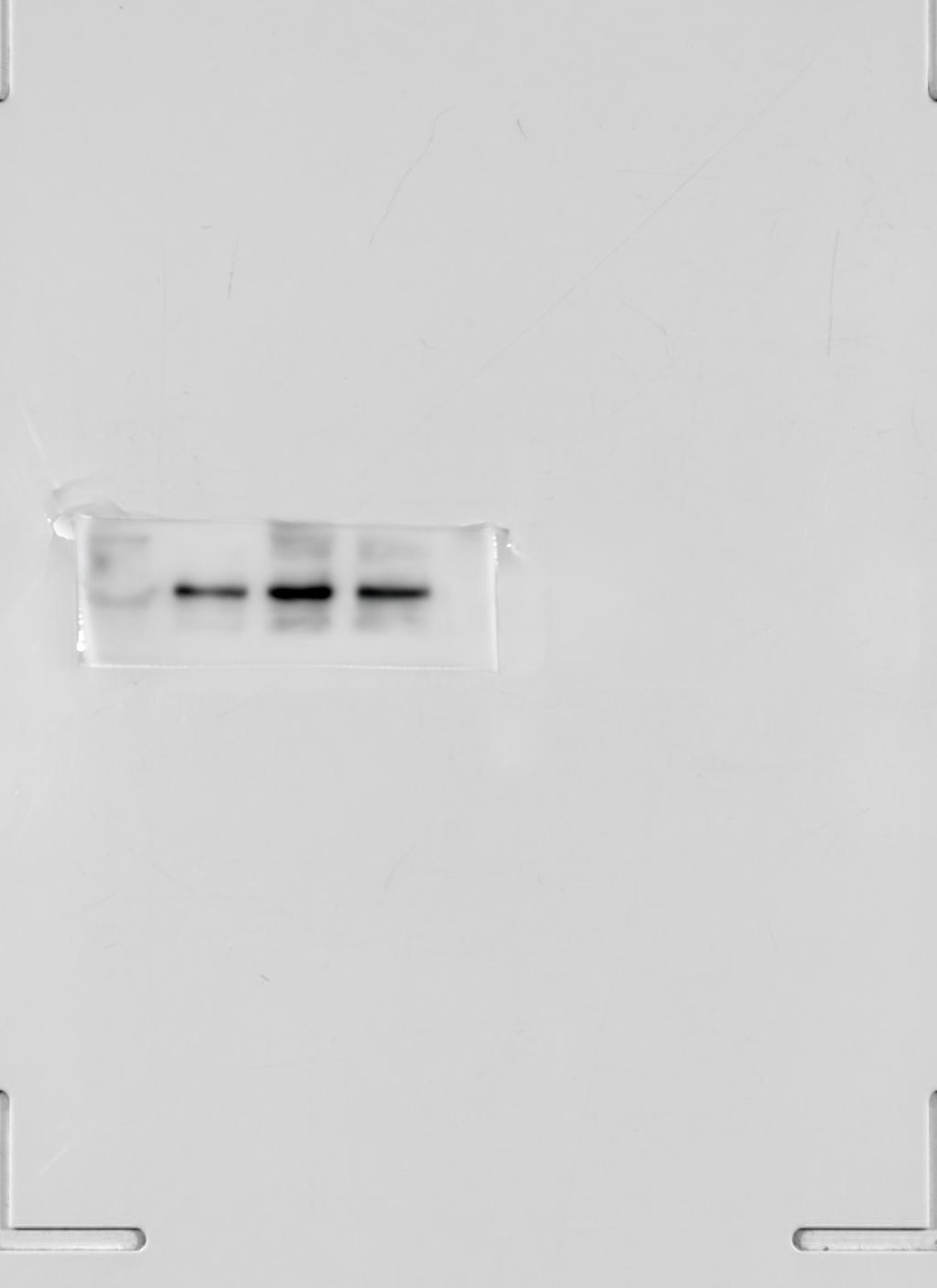

Supplement: Supplementary file 1 [file animals-15-00365-s001.zip › S1/WB original-241221/Fig7/Fig.7 BECN1-原图2.png]

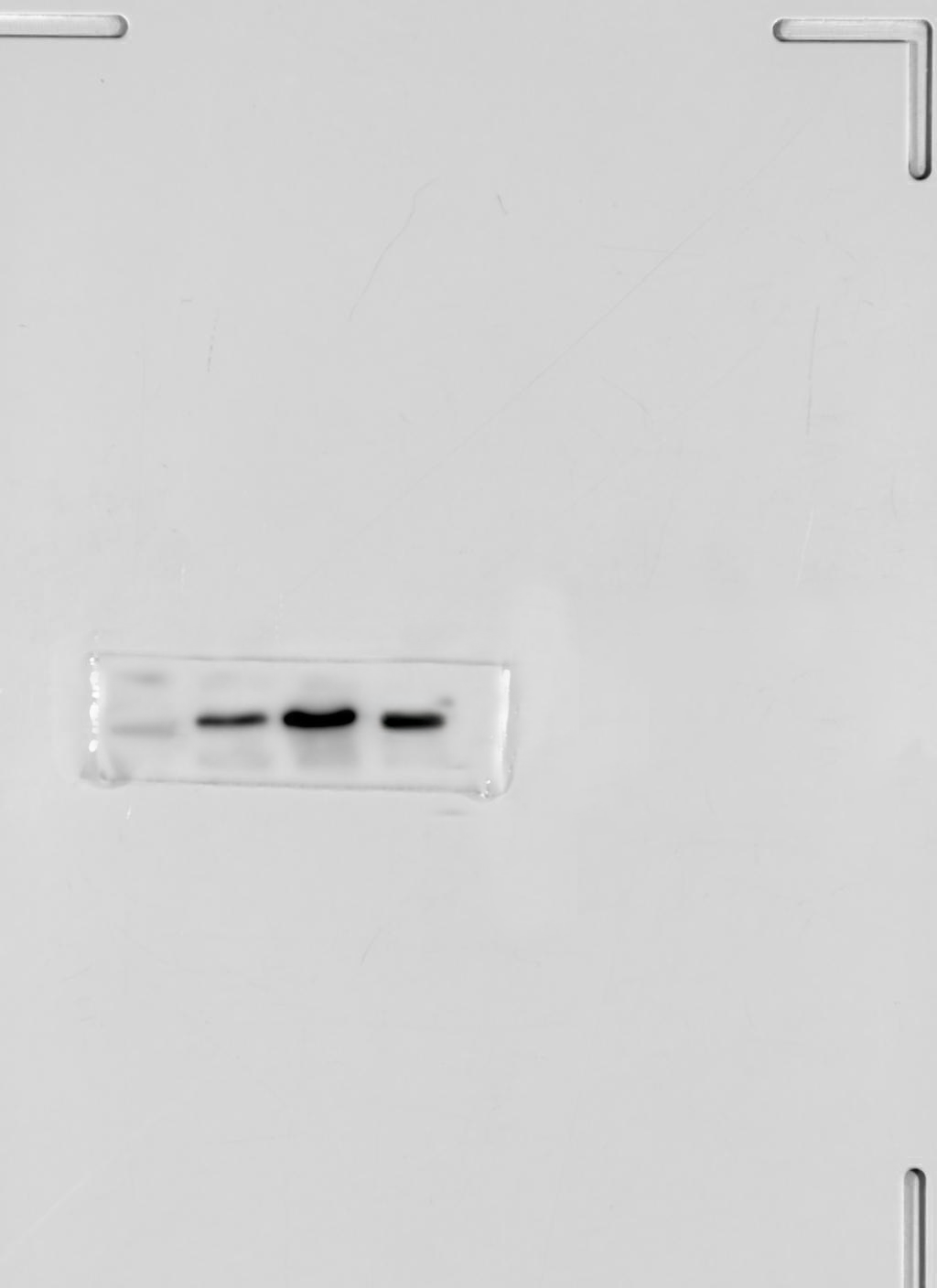

Supplement: Supplementary file 1 [file animals-15-00365-s001.zip › S1/WB original-241221/Fig7/Fig.7 BECN1-原图3.png]

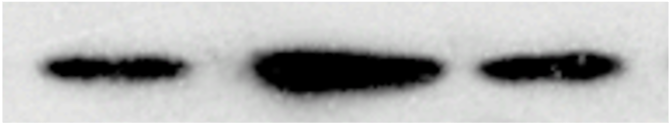

Supplement: Supplementary file 1 [file animals-15-00365-s001.zip › S1/WB original-241221/Fig7/Fig.7 BECN1.png]

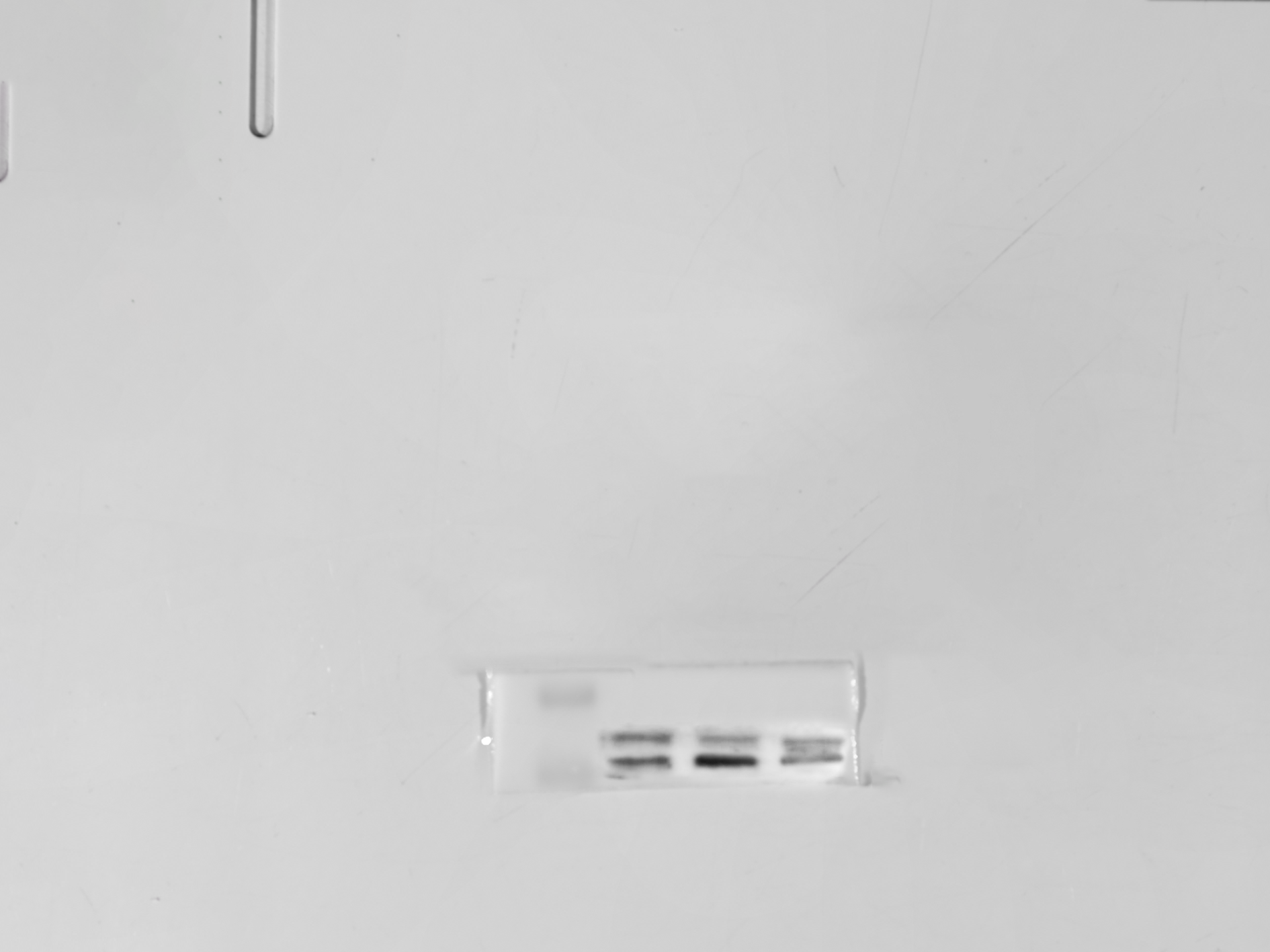

Supplement: Supplementary file 1 [file animals-15-00365-s001.zip › S1/WB original-241221/Fig7/Fig.7C LC3-I_II-原图.png]

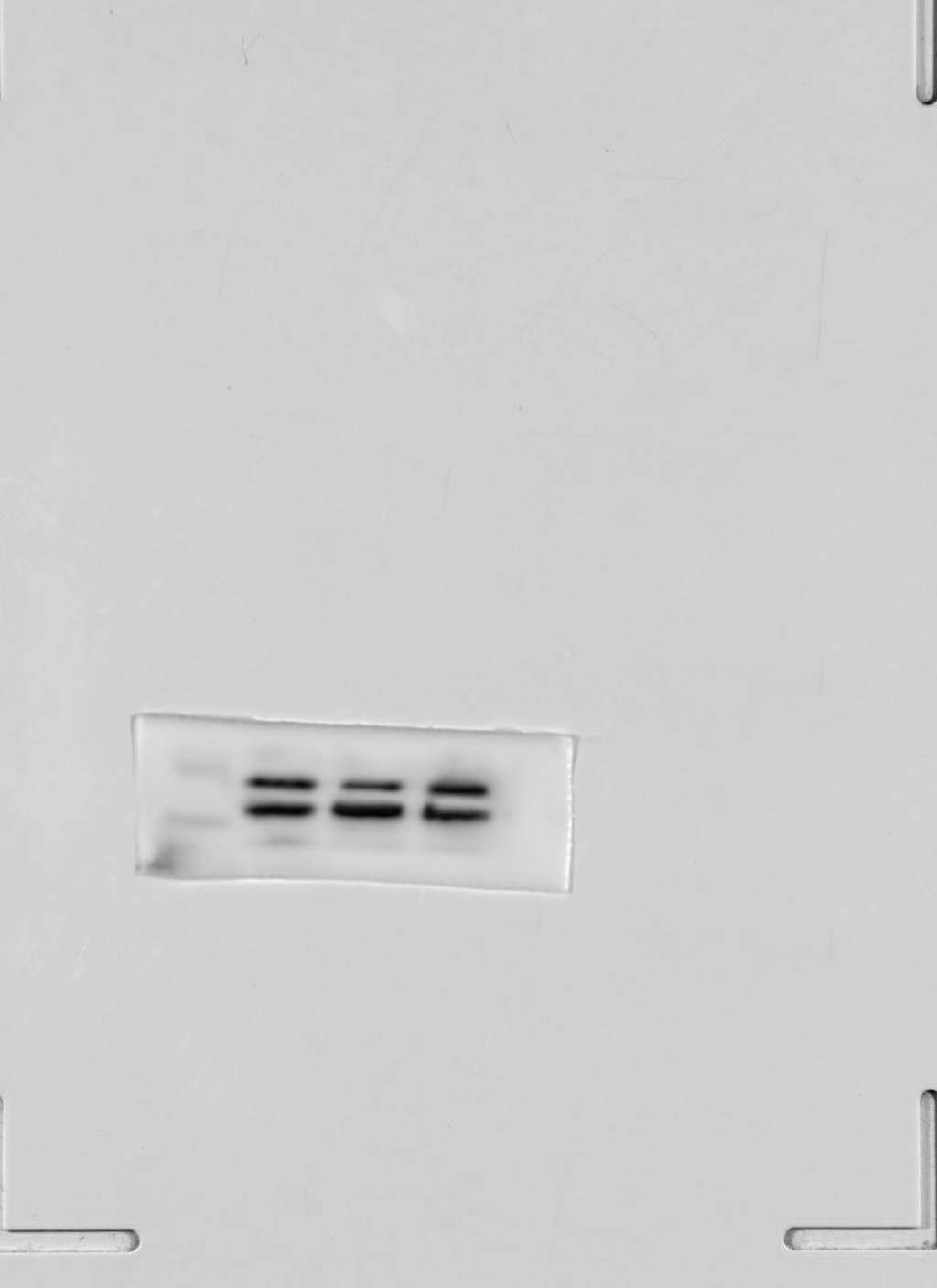

Supplement: Supplementary file 1 [file animals-15-00365-s001.zip › S1/WB original-241221/Fig7/Fig.7C LC3-I_II-原图2.png]

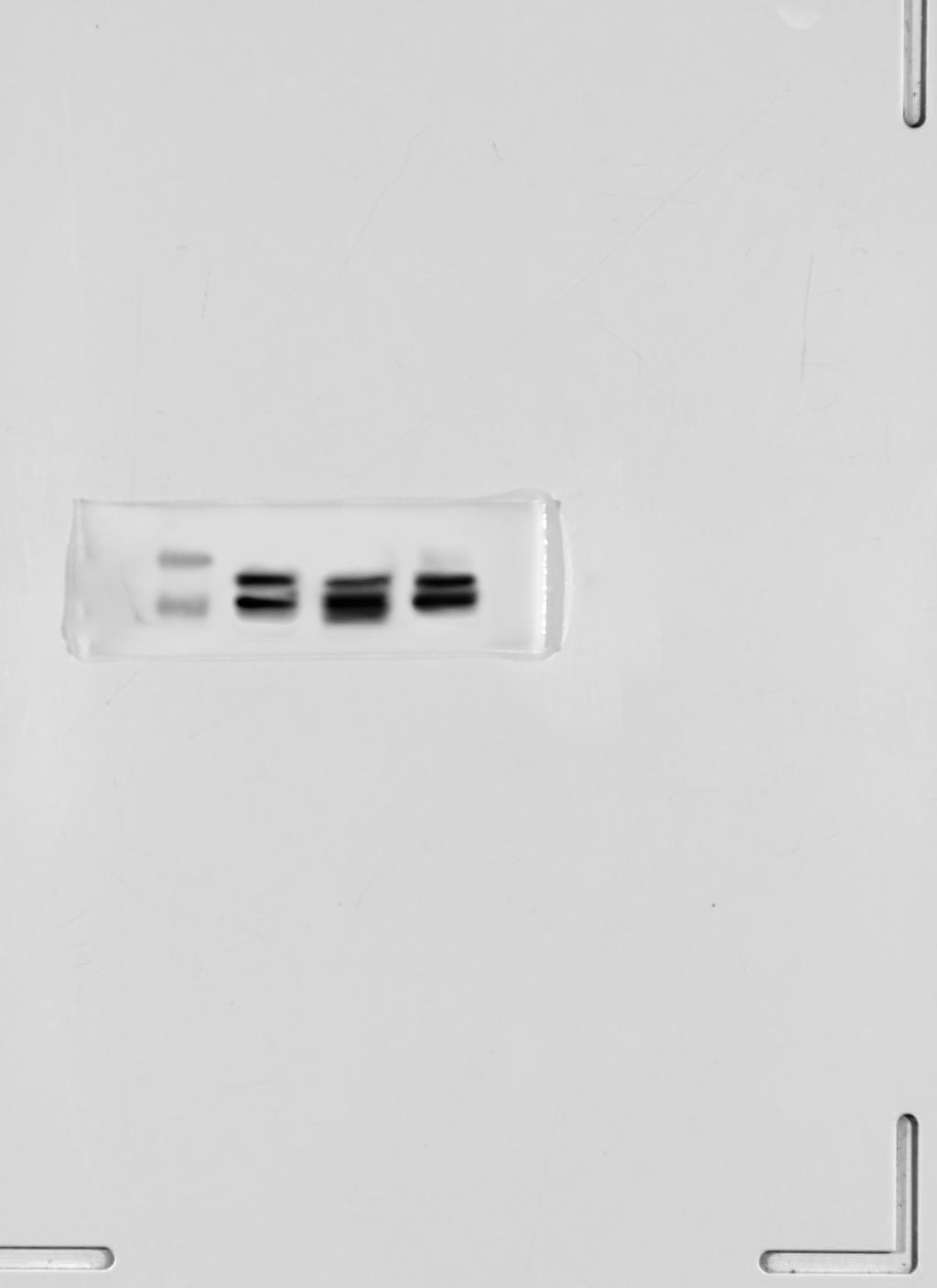

Supplement: Supplementary file 1 [file animals-15-00365-s001.zip › S1/WB original-241221/Fig7/Fig.7C LC3-I_II-原图3.png]

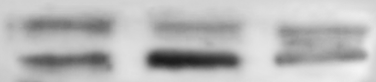

Supplement: Supplementary file 1 [file animals-15-00365-s001.zip › S1/WB original-241221/Fig7/Fig.7C LC3-I_II.png]

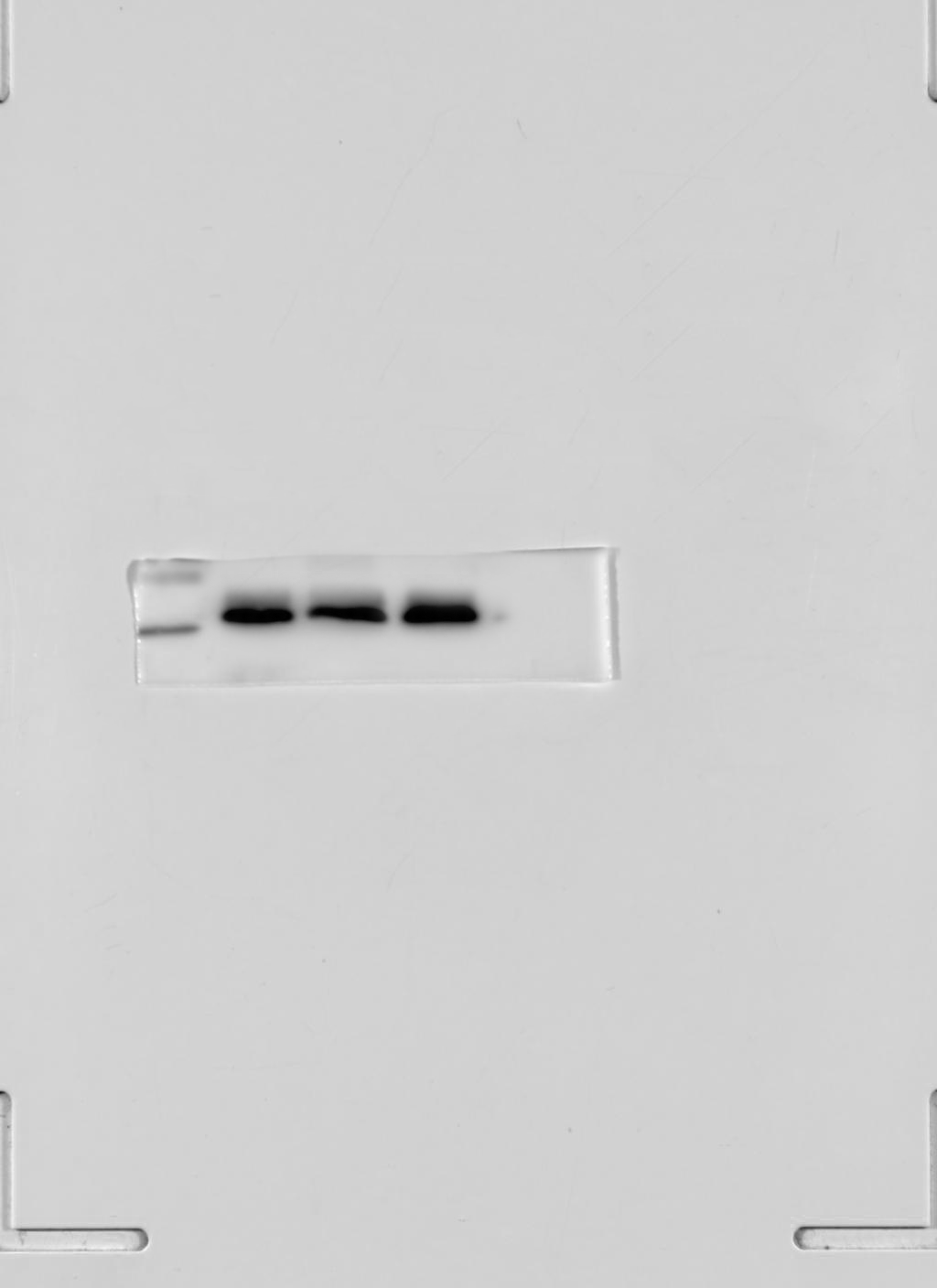

Supplement: Supplementary file 1 [file animals-15-00365-s001.zip › S1/WB original-241221/Fig8/Fig.8 actin-原图1.png]

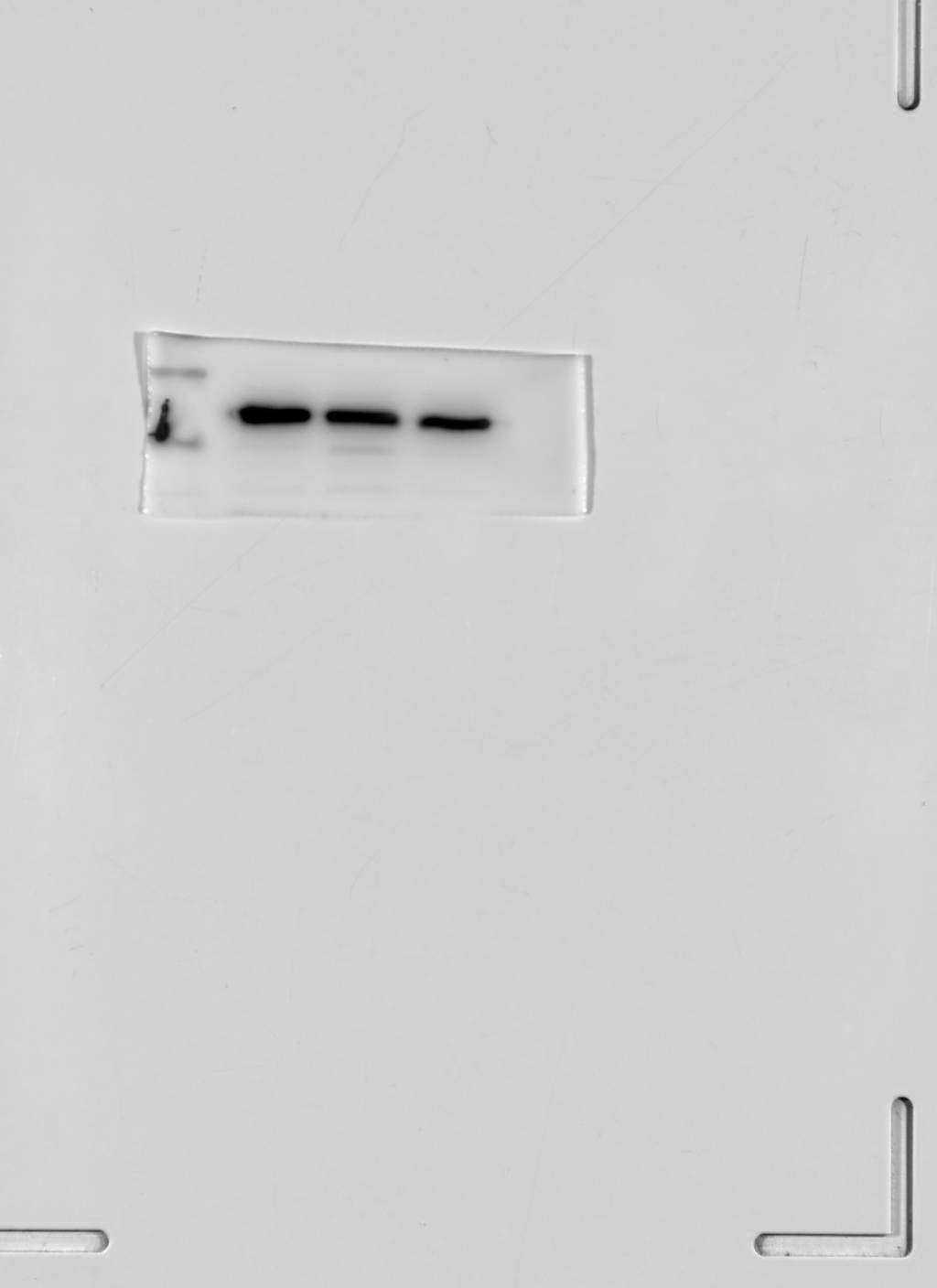

Supplement: Supplementary file 1 [file animals-15-00365-s001.zip › S1/WB original-241221/Fig8/Fig.8 actin-原图2.png]

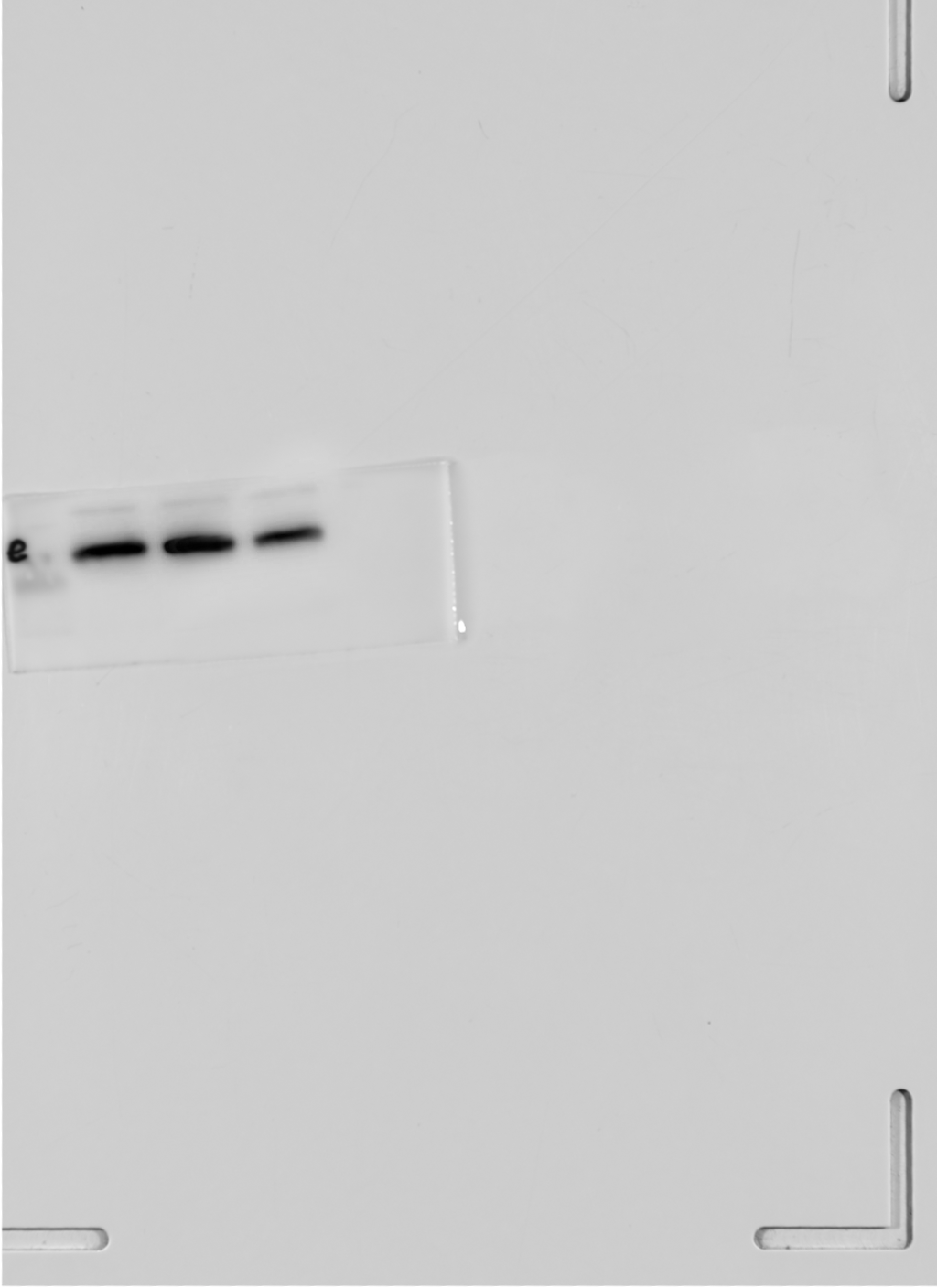

Supplement: Supplementary file 1 [file animals-15-00365-s001.zip › S1/WB original-241221/Fig8/Fig.8 actin-原图3.png]

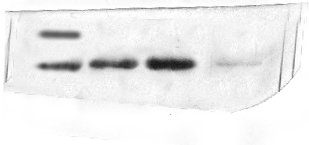

Supplement: Supplementary file 1 [file animals-15-00365-s001.zip › S1/WB original-241221/Fig8/Fig.8C CYP17A1-原图1.png]

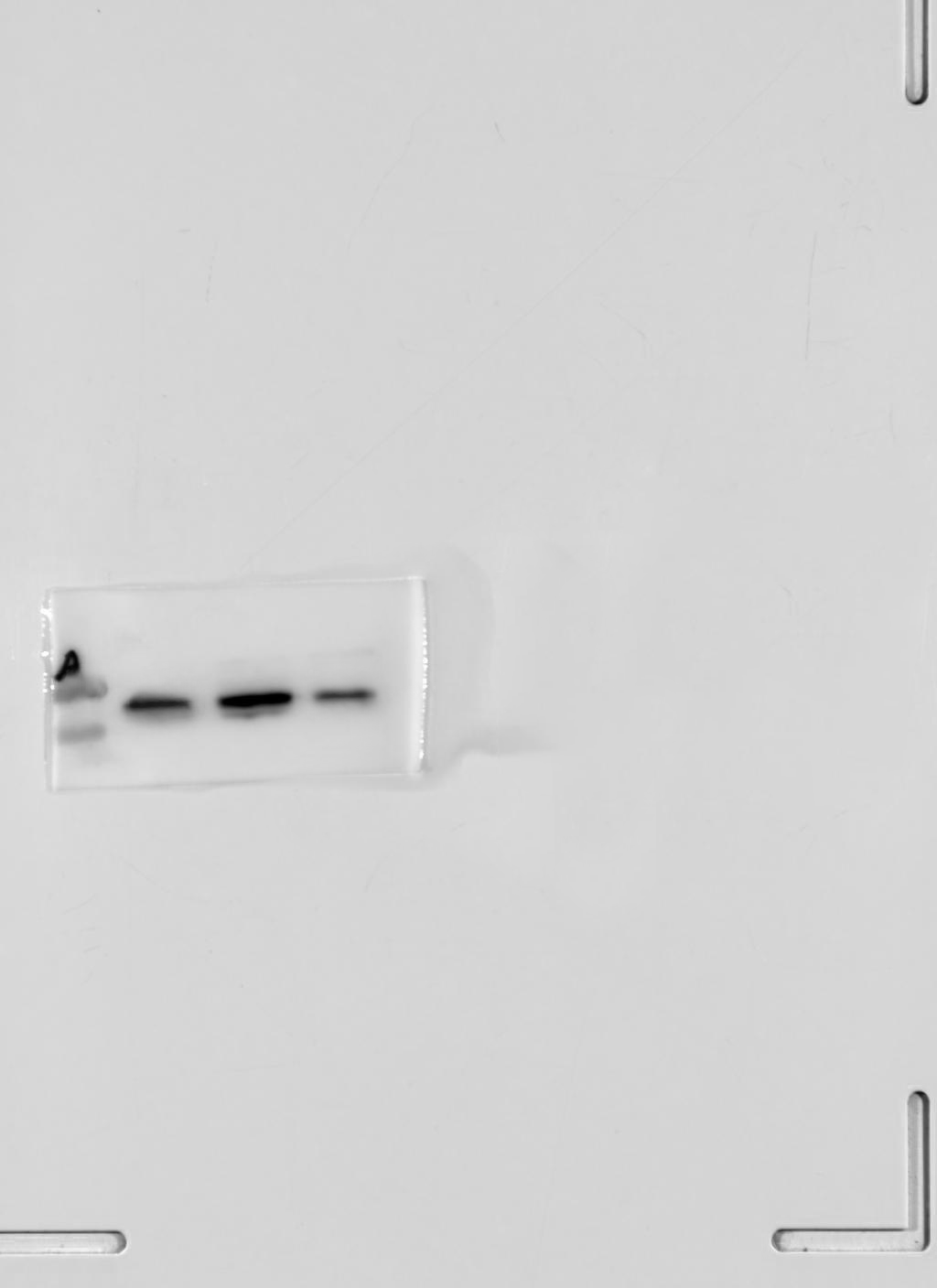

Supplement: Supplementary file 1 [file animals-15-00365-s001.zip › S1/WB original-241221/Fig8/Fig.8C CYP17A1-原图2.png]

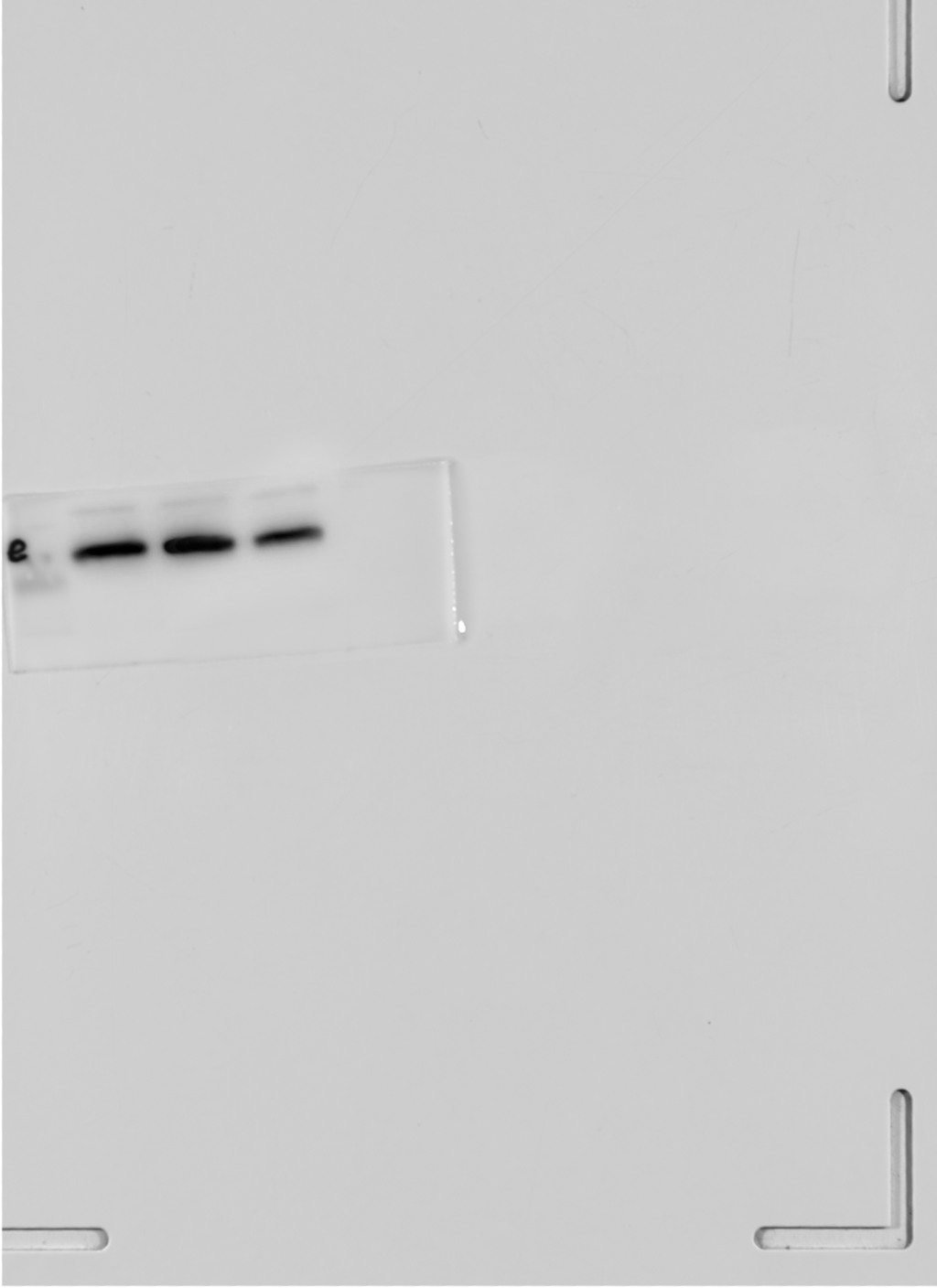

Supplement: Supplementary file 1 [file animals-15-00365-s001.zip › S1/WB original-241221/Fig8/Fig.8C CYP17A1-原图3.png]

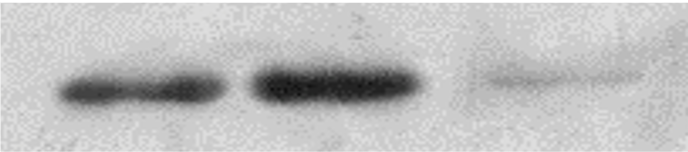

Supplement: Supplementary file 1 [file animals-15-00365-s001.zip › S1/WB original-241221/Fig8/Fig.8C CYP17A1.png]

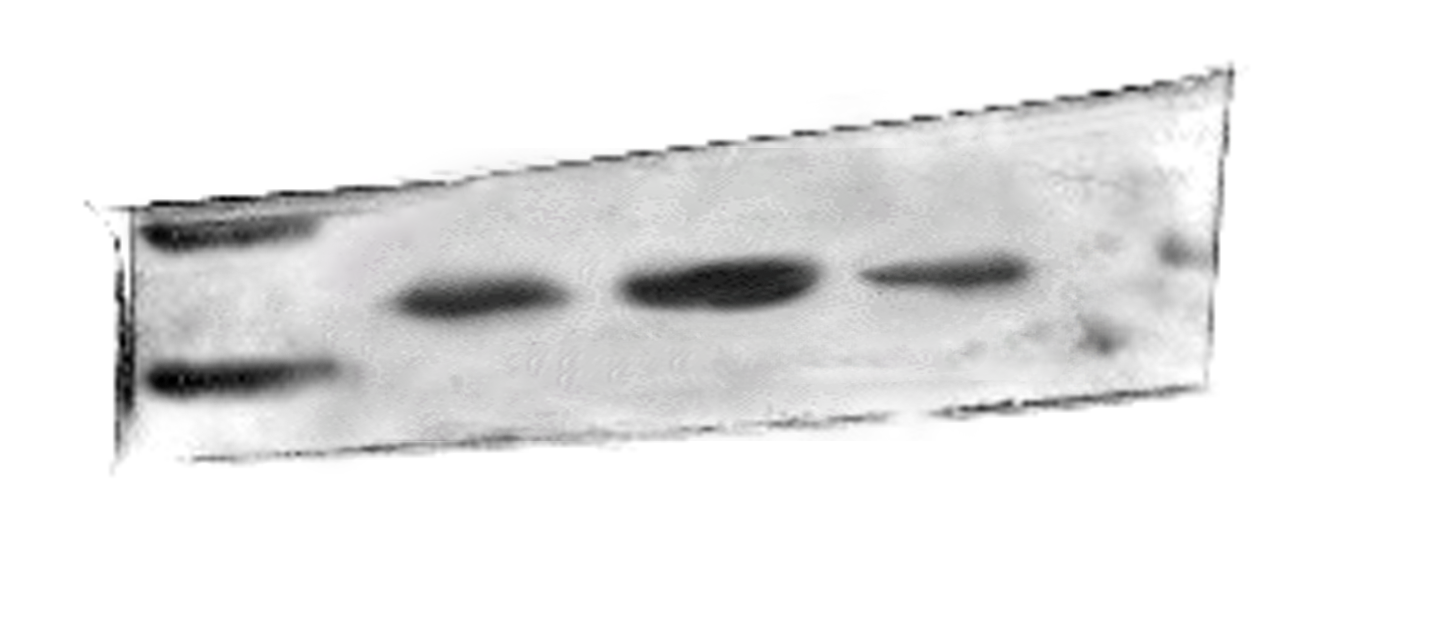

Supplement: Supplementary file 1 [file animals-15-00365-s001.zip › S1/WB original-241221/Fig8/Fig.8C CYP19A1-原图1.png]

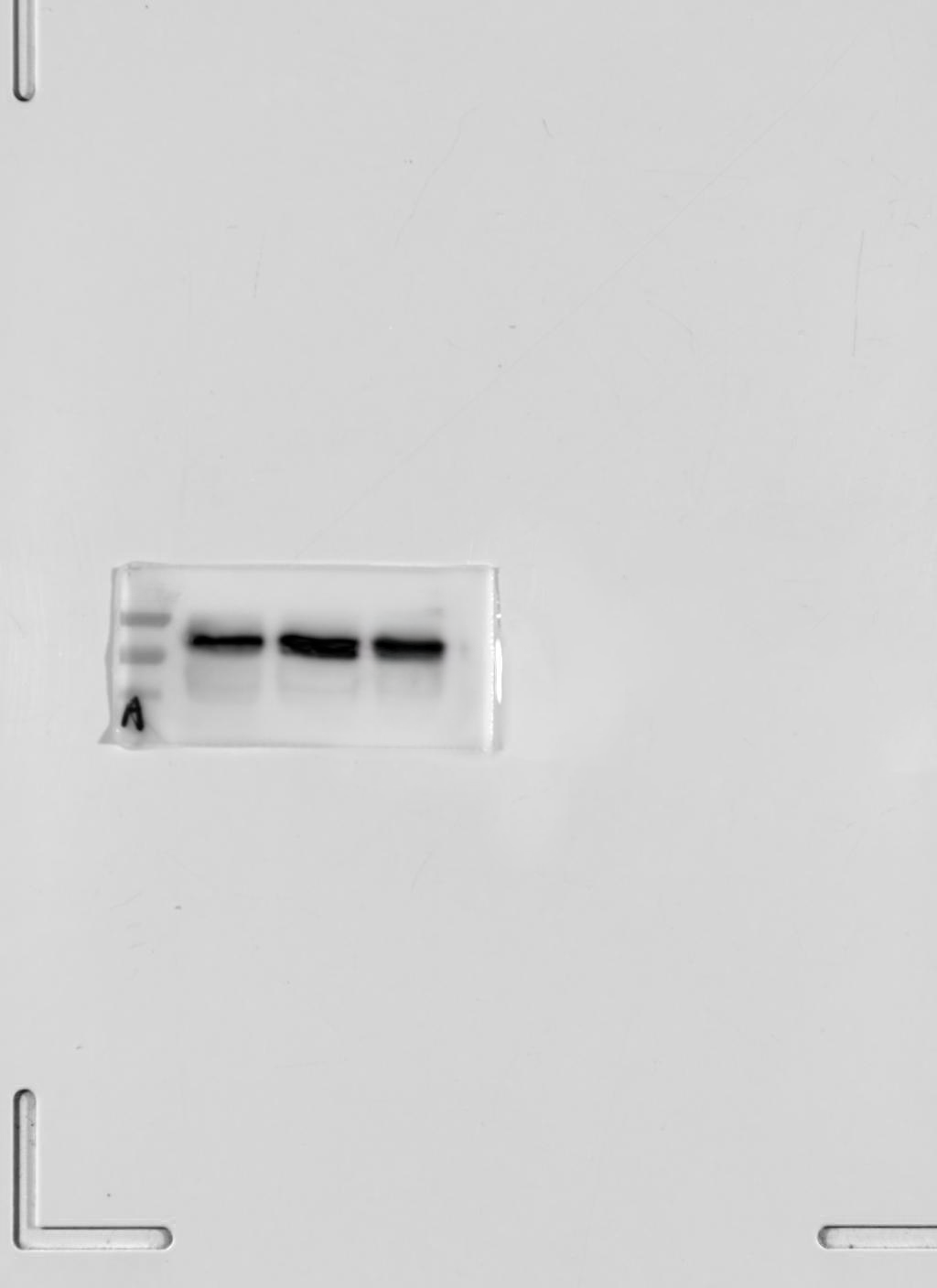

Supplement: Supplementary file 1 [file animals-15-00365-s001.zip › S1/WB original-241221/Fig8/Fig.8C CYP19A1-原图2.png]

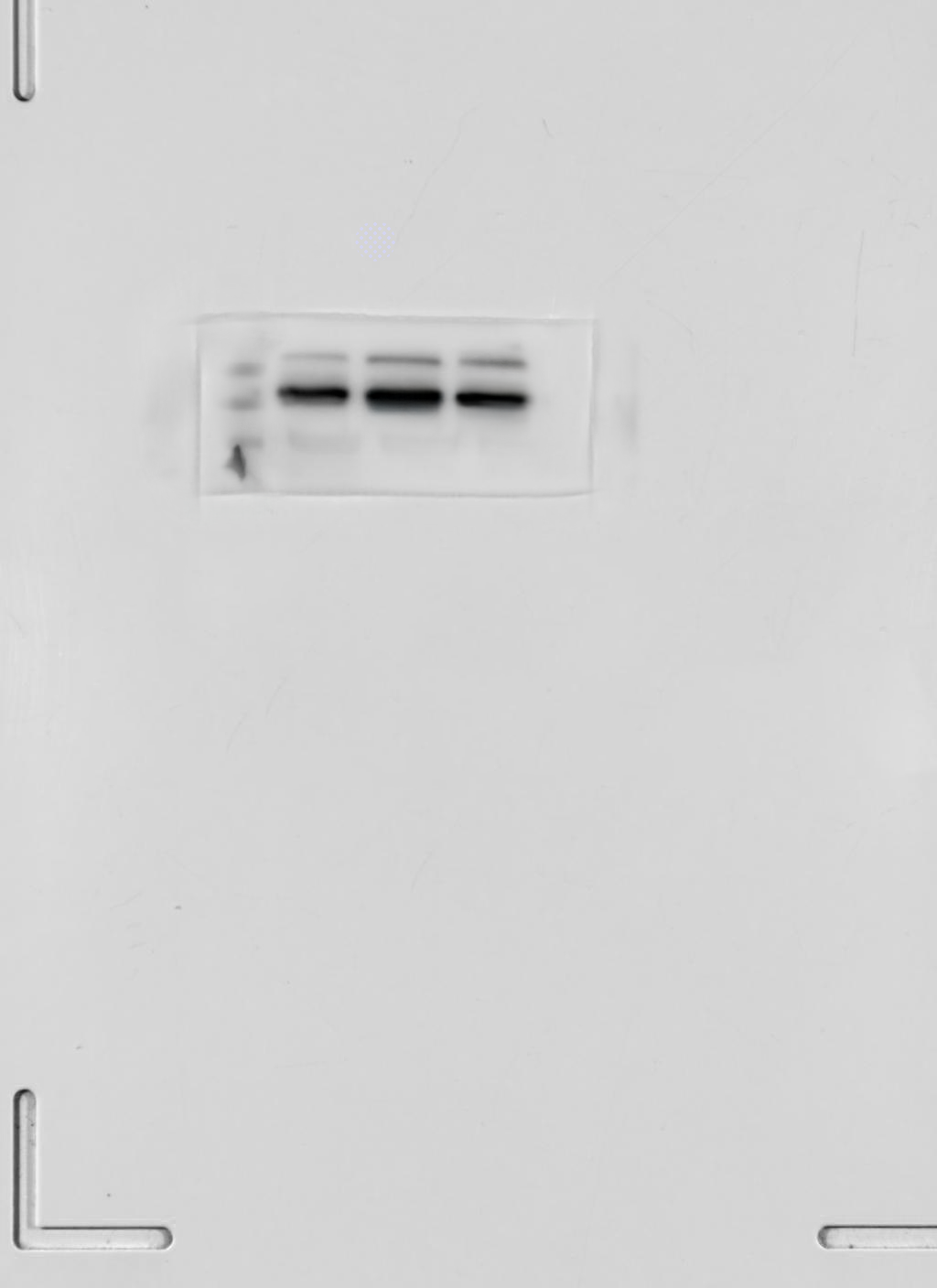

Supplement: Supplementary file 1 [file animals-15-00365-s001.zip › S1/WB original-241221/Fig8/Fig.8C CYP19A1-原图3.png]

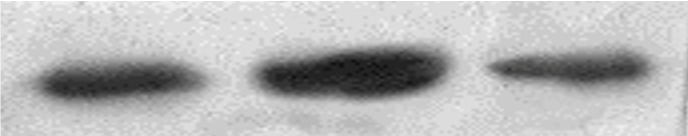

Supplement: Supplementary file 1 [file animals-15-00365-s001.zip › S1/WB original-241221/Fig8/Fig.8C CYP19A1.png]

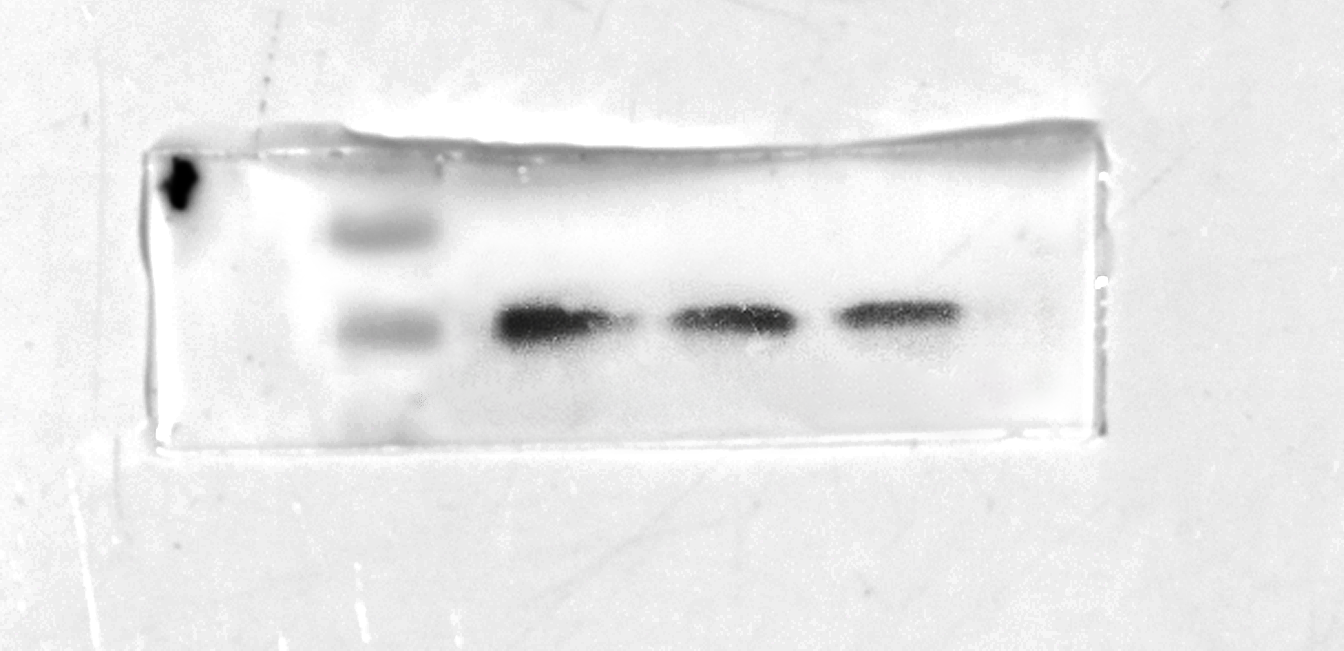

Supplement: Supplementary file 1 [file animals-15-00365-s001.zip › S1/WB original-241221/Fig9/Fig.9C actin-原图1.png]

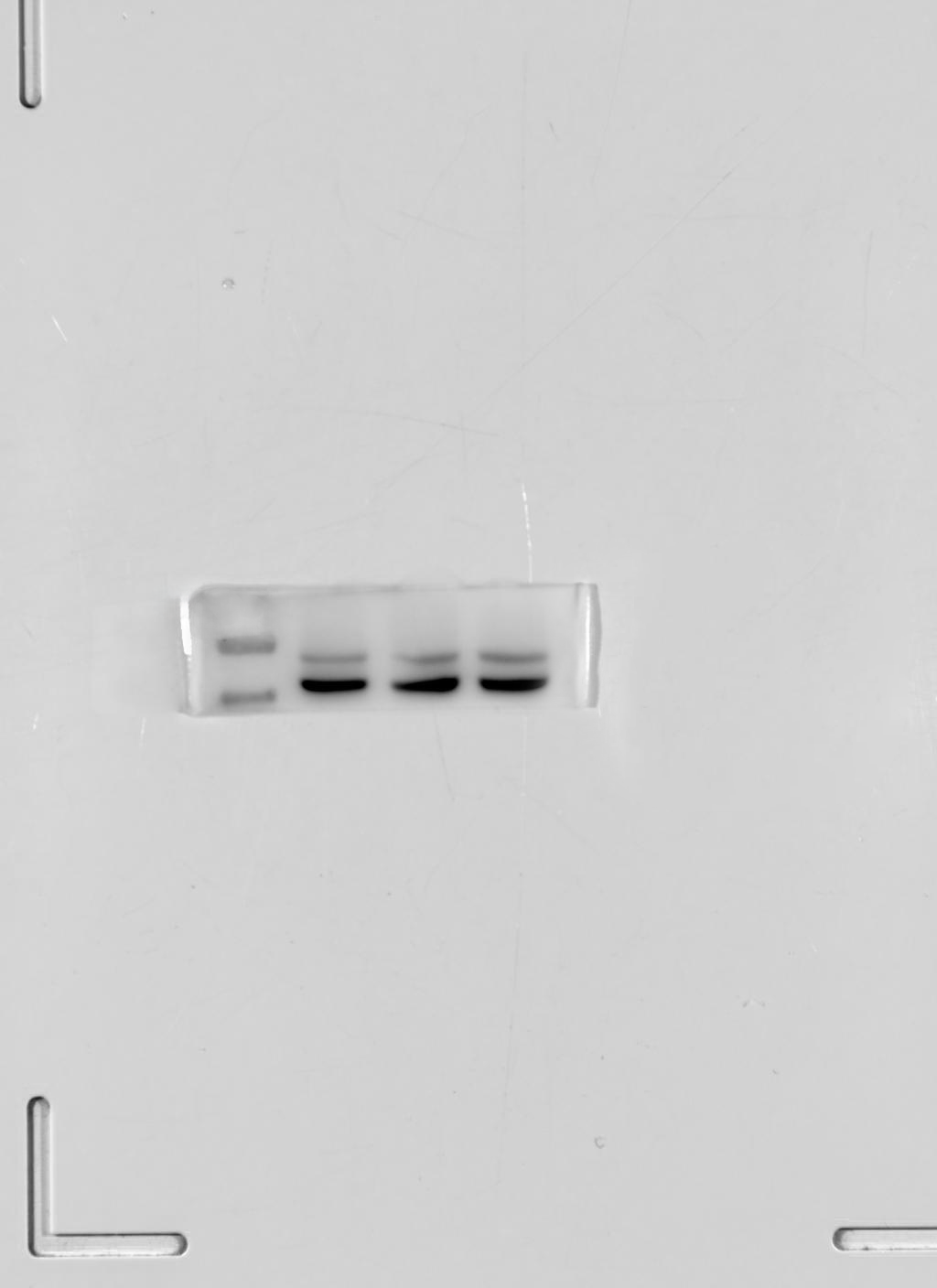

Supplement: Supplementary file 1 [file animals-15-00365-s001.zip › S1/WB original-241221/Fig9/Fig.9C actin-原图2.png]

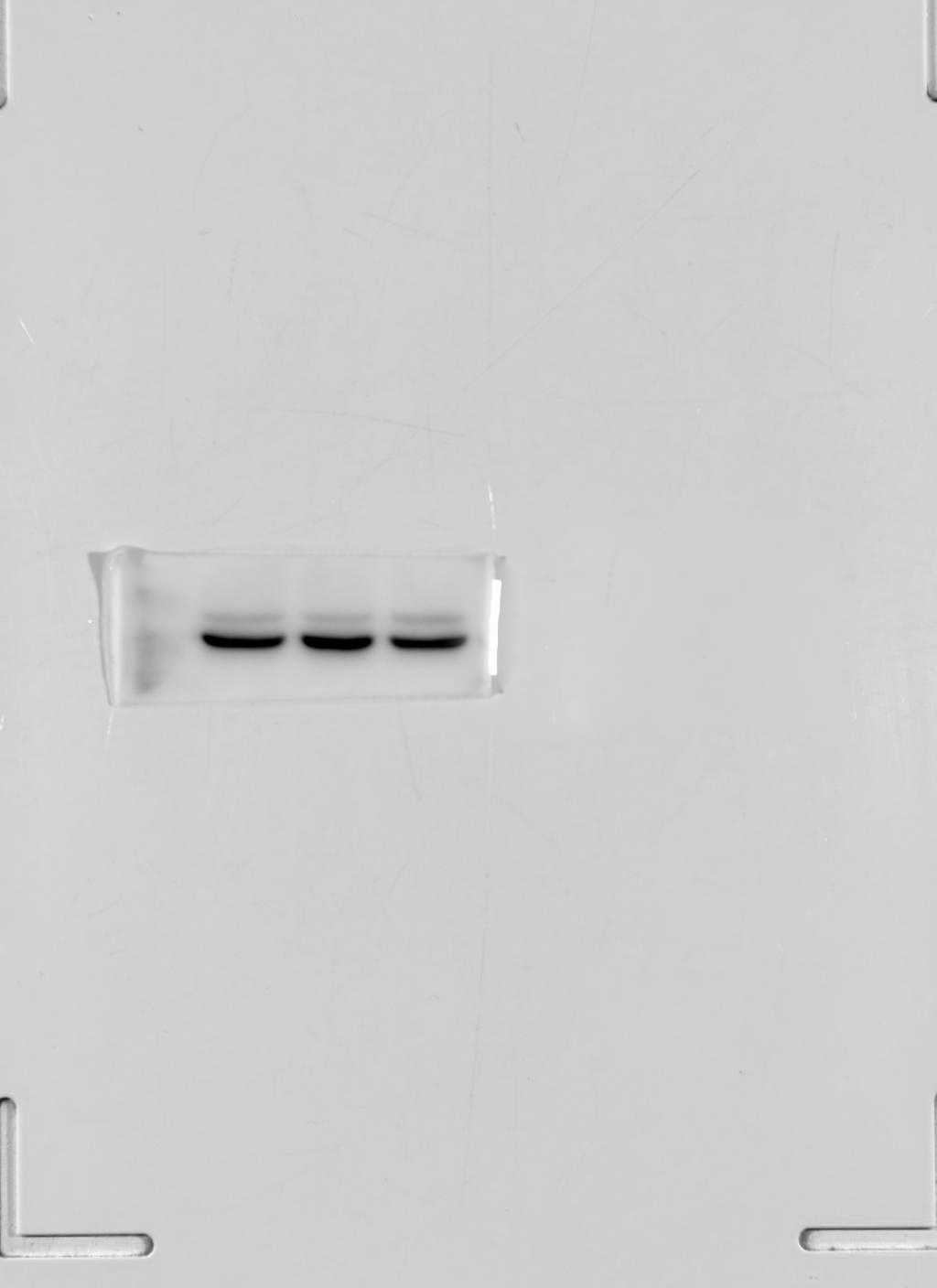

Supplement: Supplementary file 1 [file animals-15-00365-s001.zip › S1/WB original-241221/Fig9/Fig.9C actin-原图3.png]

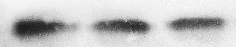

Supplement: Supplementary file 1 [file animals-15-00365-s001.zip › S1/WB original-241221/Fig9/Fig.9C actin.png]

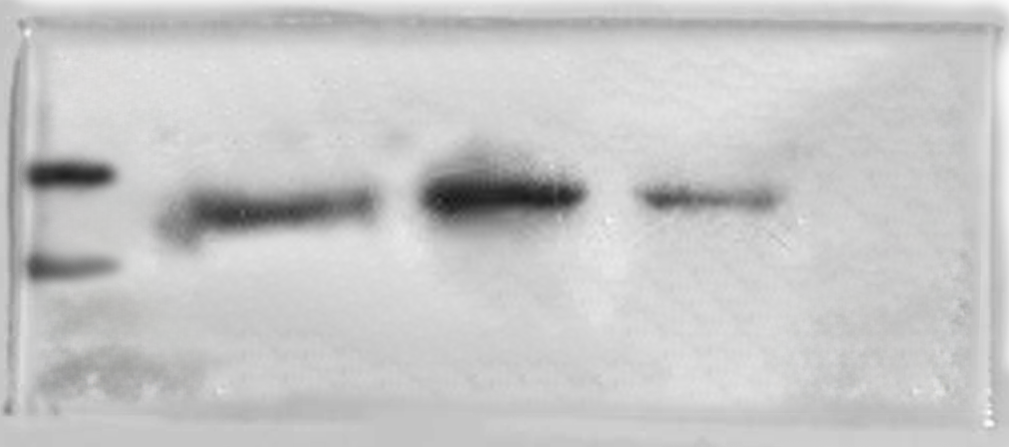

Supplement: Supplementary file 1 [file animals-15-00365-s001.zip › S1/WB original-241221/Fig9/Fig.9C ATG5-原图1.png]

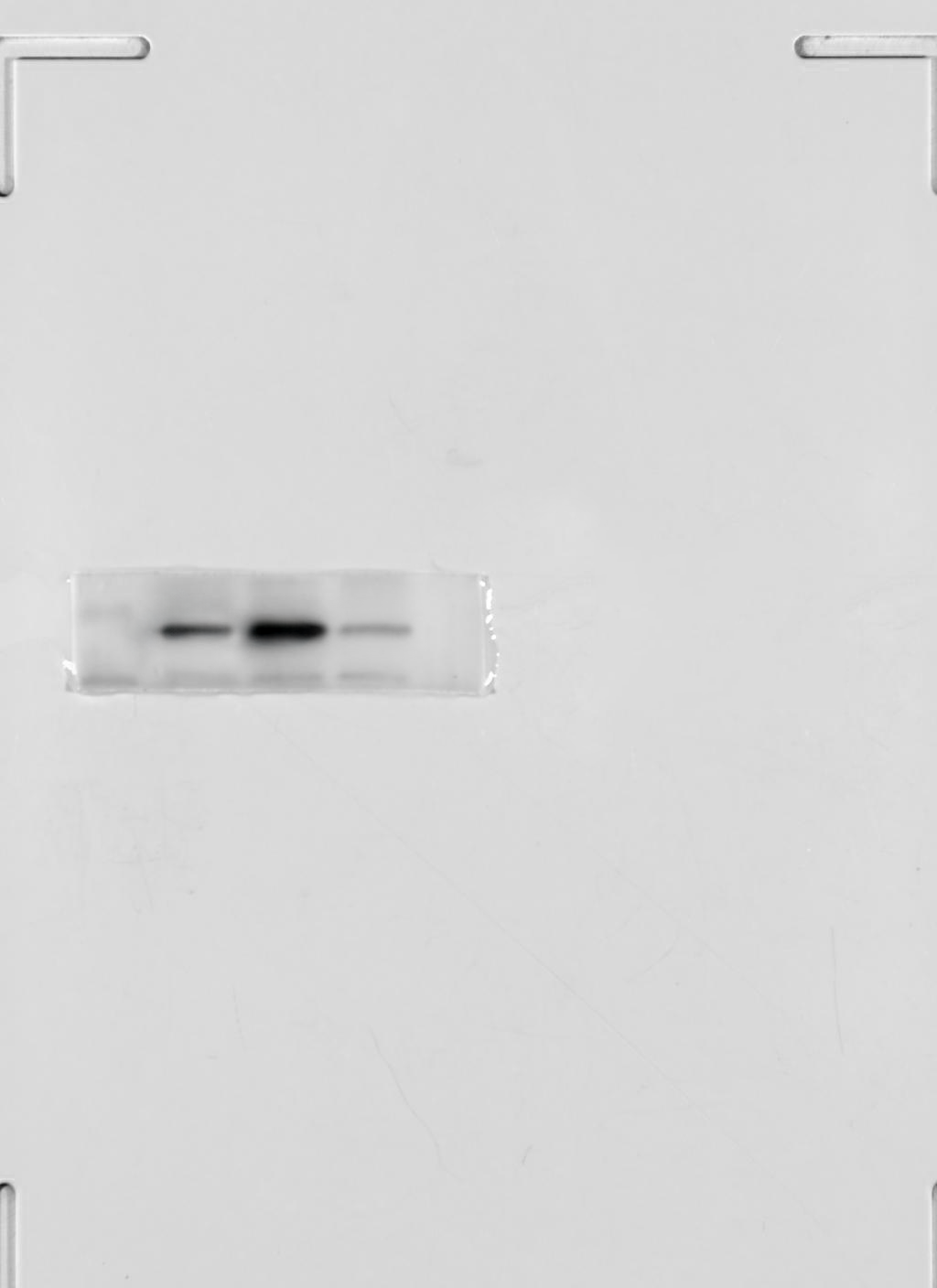

Supplement: Supplementary file 1 [file animals-15-00365-s001.zip › S1/WB original-241221/Fig9/Fig.9C ATG5-原图2.png]

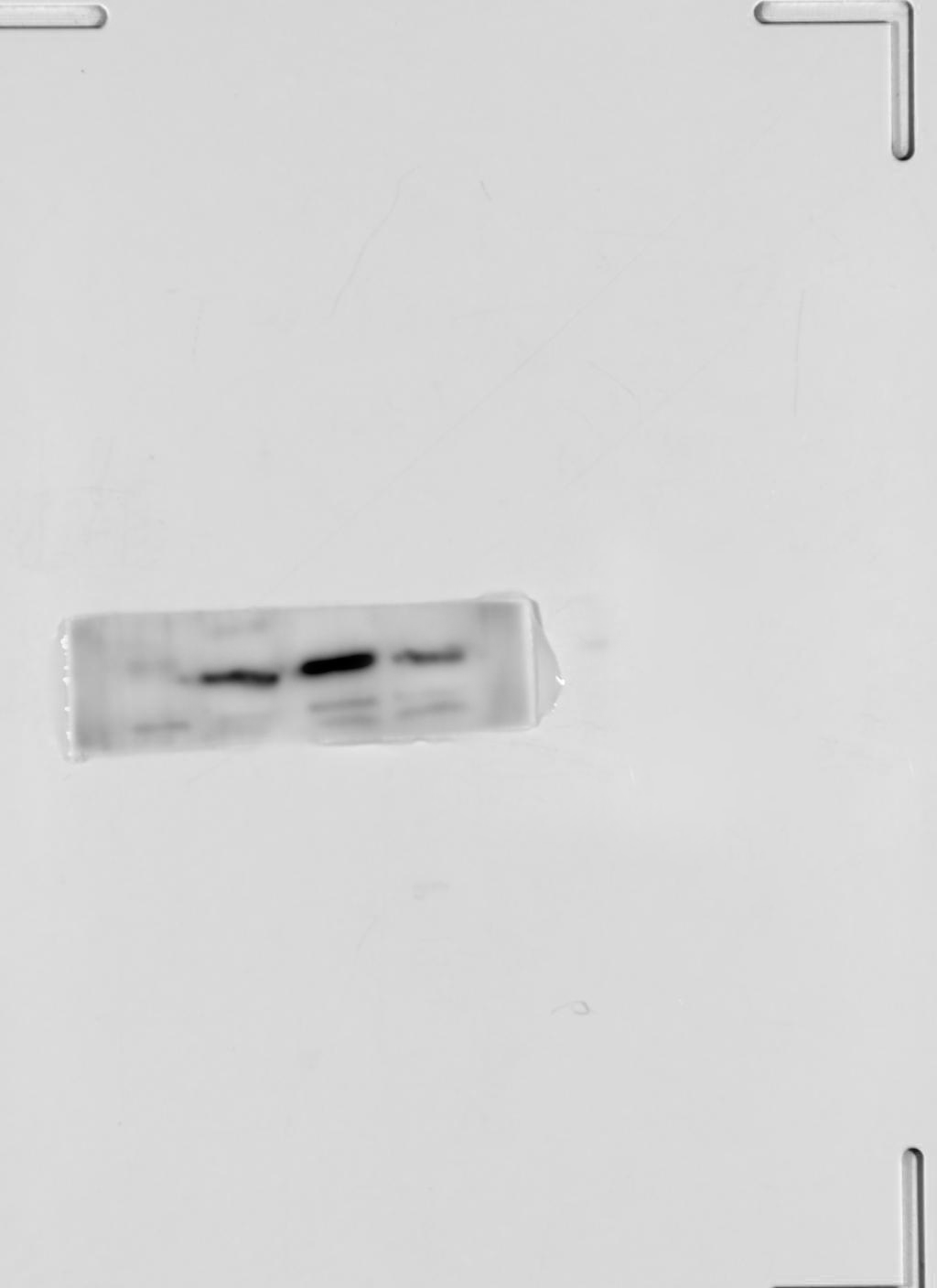

Supplement: Supplementary file 1 [file animals-15-00365-s001.zip › S1/WB original-241221/Fig9/Fig.9C ATG5-原图3.png]

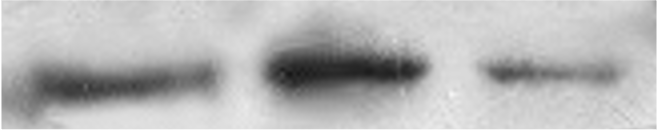

Supplement: Supplementary file 1 [file animals-15-00365-s001.zip › S1/WB original-241221/Fig9/Fig.9C ATG5.png]

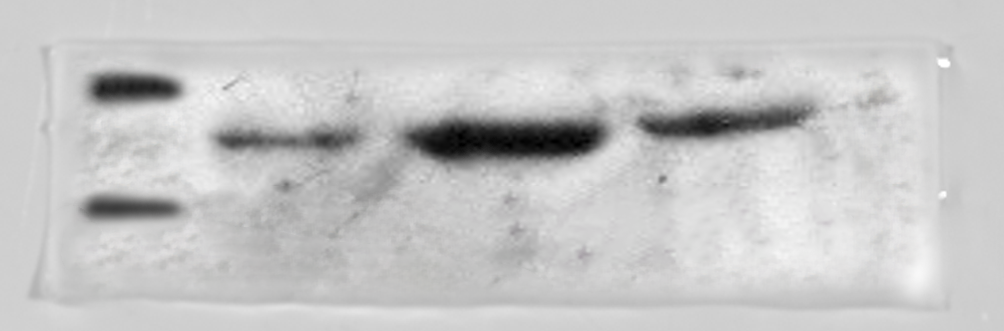

Supplement: Supplementary file 1 [file animals-15-00365-s001.zip › S1/WB original-241221/Fig9/Fig.9C BECN1-原图.png]

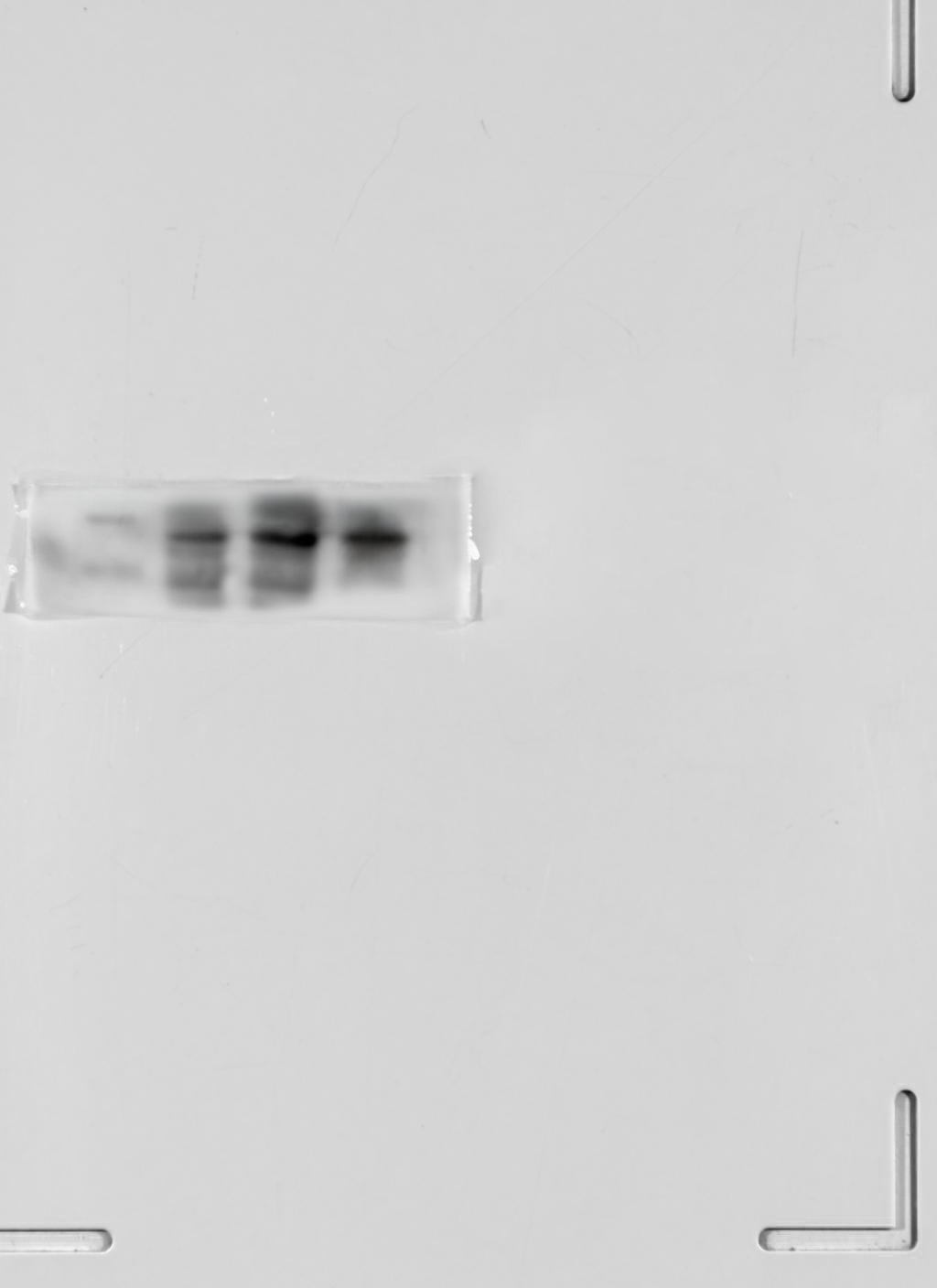

Supplement: Supplementary file 1 [file animals-15-00365-s001.zip › S1/WB original-241221/Fig9/Fig.9C BECN1-原图2.png]

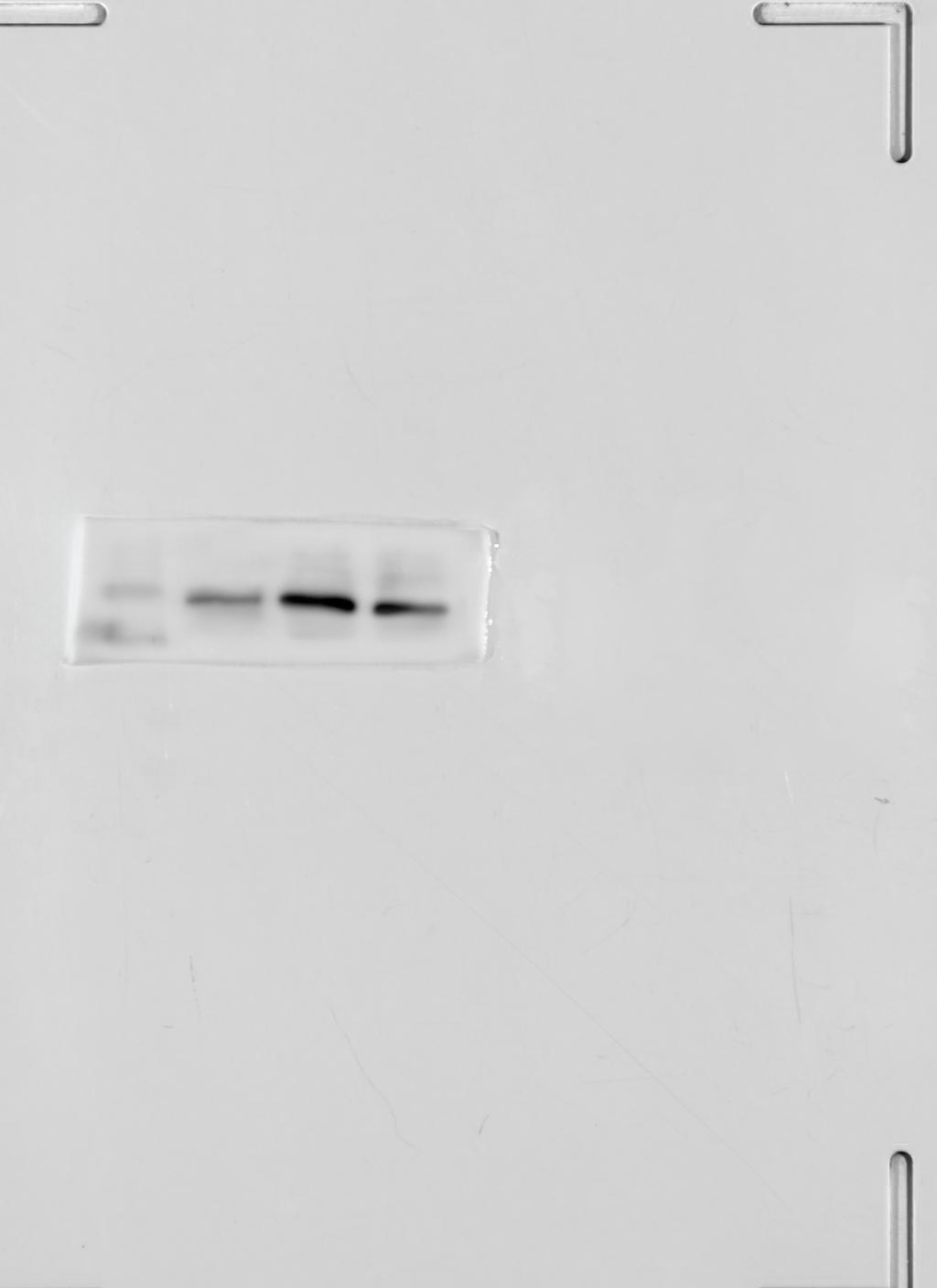

Supplement: Supplementary file 1 [file animals-15-00365-s001.zip › S1/WB original-241221/Fig9/Fig.9C BECN1-原图3.png]

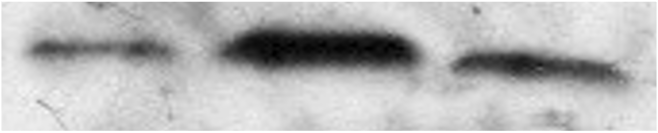

Supplement: Supplementary file 1 [file animals-15-00365-s001.zip › S1/WB original-241221/Fig9/Fig.9C BECN1.png]

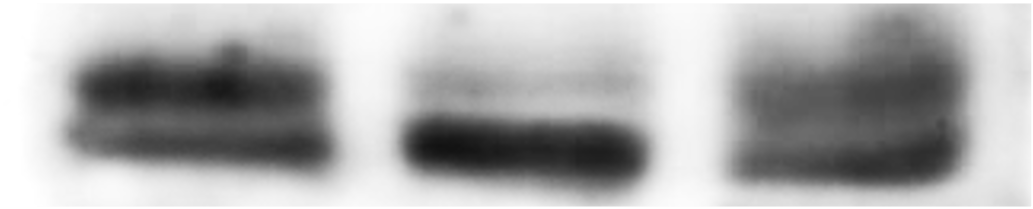

Supplement: Supplementary file 1 [file animals-15-00365-s001.zip › S1/WB original-241221/Fig9/Fig.9C LC3-I_-II.png]

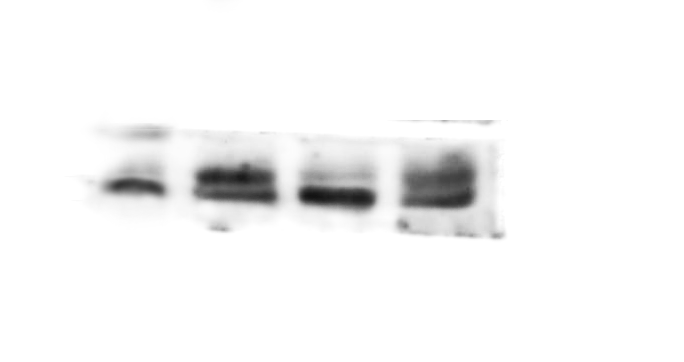

Supplement: Supplementary file 1 [file animals-15-00365-s001.zip › S1/WB original-241221/Fig9/Fig.9C LC3-I_-II原图.png]

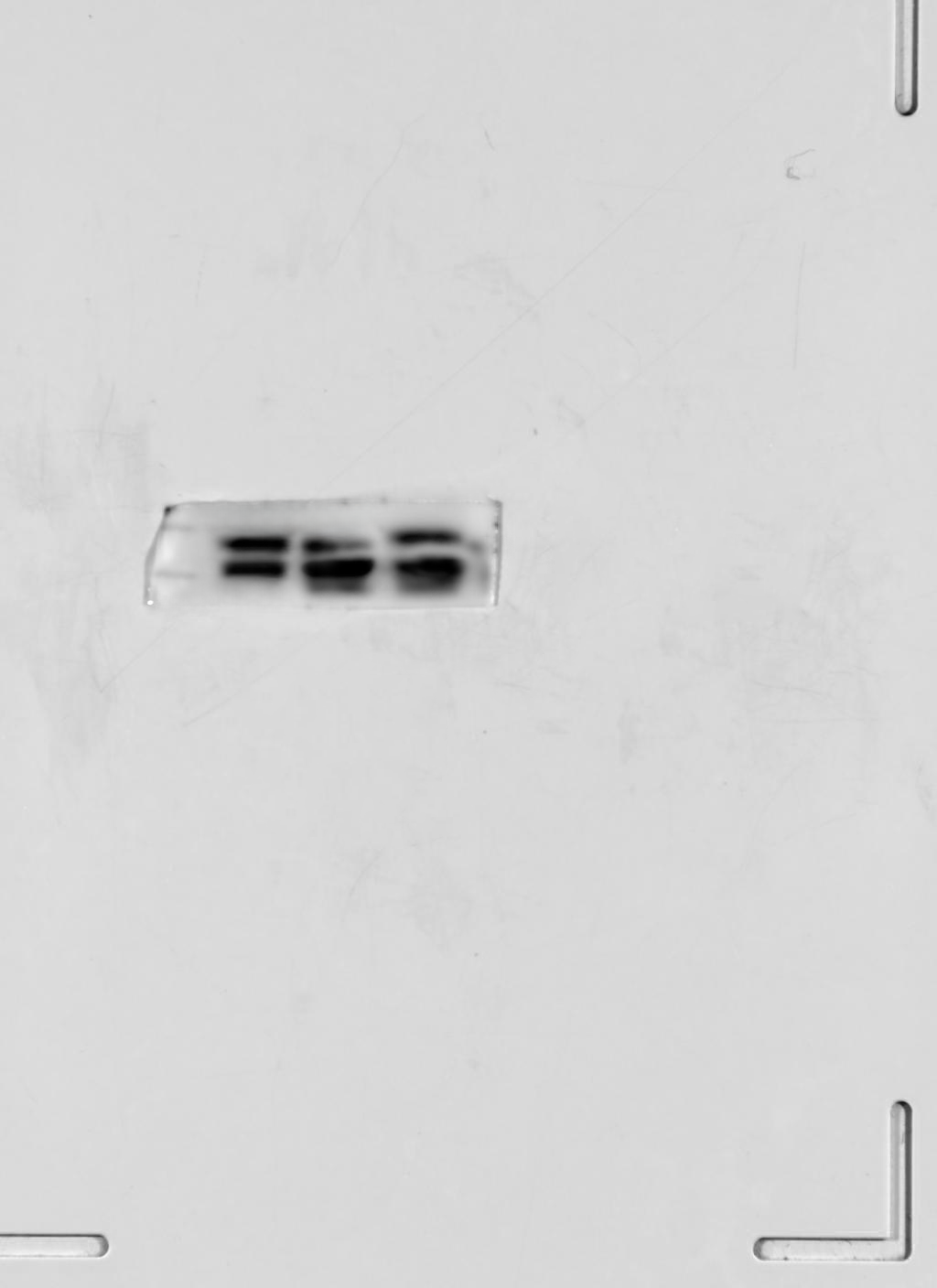

Supplement: Supplementary file 1 [file animals-15-00365-s001.zip › S1/WB original-241221/Fig9/Fig.9C LC3-I_-II原图2.png]

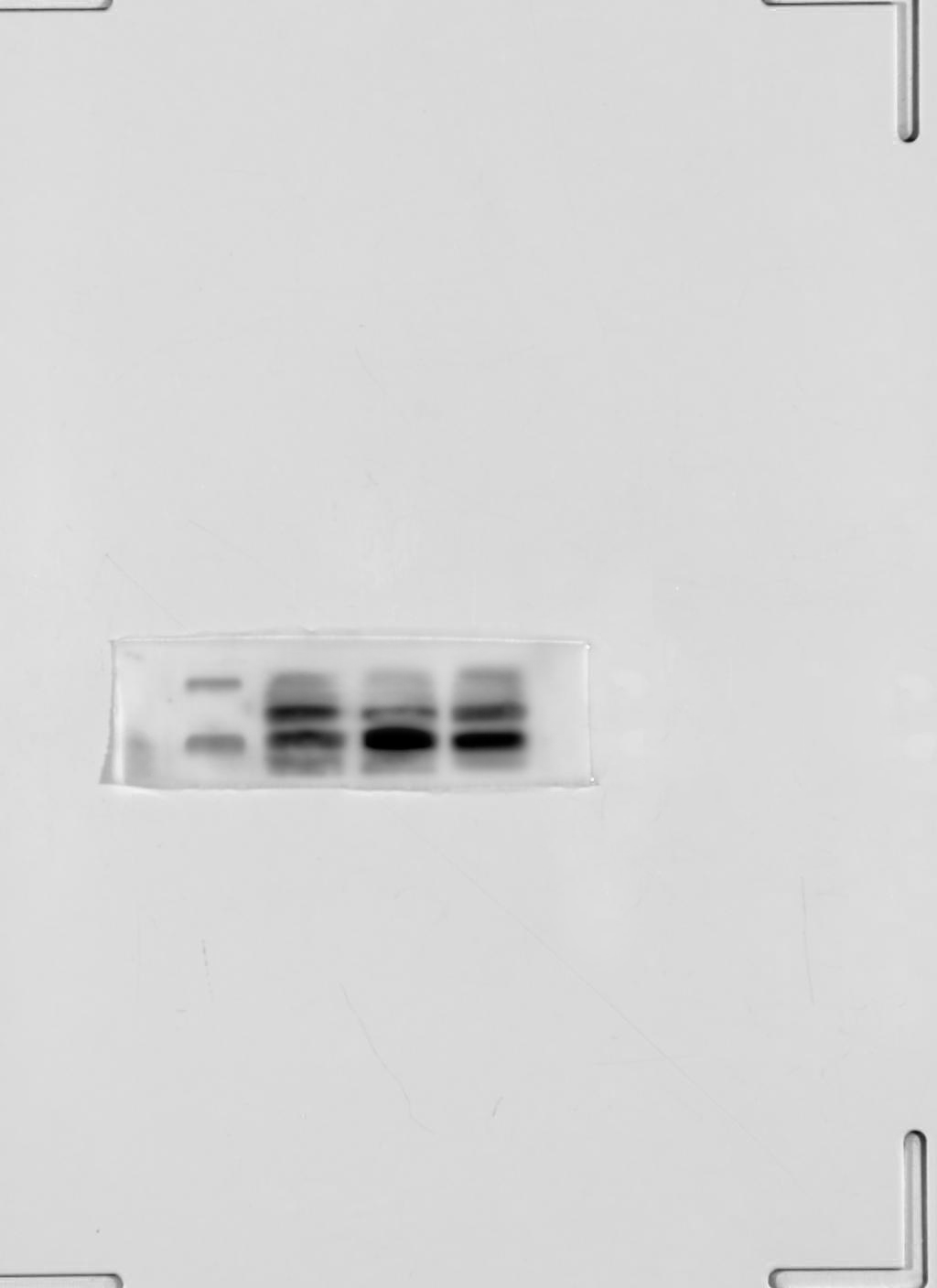

Supplement: Supplementary file 1 [file animals-15-00365-s001.zip › S1/WB original-241221/Fig9/Fig.9C LC3-I_-II原图3.png]
